# Effectiveness of m-health-based core strengthening exercise and health education for public safety workers with chronic non-specific low back pain: study protocol for a superiority randomized controlled trial (SAFEBACK)

**DOI:** 10.1186/s13063-023-07833-9

**Published:** 2023-12-01

**Authors:** Eduardo F. Marins, Eduardo L. Caputo, Vitor L. Krüger, Dirceu M. Junior, Fabrício G. Scaglioni, Fabricio B. Del Vecchio, Tiago T. Primo, Cristine L. Alberton

**Affiliations:** 1 Postgraduate Program in Physical Education, Universidade Federal de Pelotas, Physical Education School, Rua Luiz de Camões 625, Pelotas, Rio Grande Do Sul 96055-630 Brazil; 2 Postgraduate Program in Computing, Universidade Federal de Pelotas, Rua Gomes Carneiro, 1, Pelotas, Rio Grande Do Sul 96010-610 Brazil

**Keywords:** Low back pain, Mobile health, Physical exercise

## Abstract

**Background:**

Low back pain (LBP) is the leading cause of years lived with disability worldwide. Public safety workers are highly exposed to physically demanding activities and inappropriate postures, increasing the risk of experiencing LBP. Smartphone app-based self-managed interventions may be an alternative for chronic non-specific LBP (CNSLBP) treatment. This study aims to evaluate the effectiveness of a smartphone app-based self-managed exercise program plus health education, compared to a health education program alone, on neuromuscular and perceptual outcomes in police officers and firefighters with CNSLBP.

**Methods:**

This is a parallel, two-armed, blinded evaluator randomized clinical trial. Police officers and firefighters (from public safety institutions in the Rio Grande do Sul state, Brazil) will be randomly assigned to a m-health self-managed exercise program (twice a week) plus health education or health education alone. Self-management exercise program components are mobility and core resistance exercises, available on the app. Follow-ups will be conducted post-treatment (8 weeks) and 16 weeks after randomization. The co-primary outcomes will be pain intensity and disability post-treatment (8 weeks). Secondary outcomes will be biopsychosocial factors related to CNSLBP.

**Discussion:**

We hypothesize that the effects of a smartphone app-based self-managed exercise program on co-primary and secondary outcomes will be superior, compared to the health education only in public safety workers with CNSLBP.

**Trial registration:**

The study was prospectively registered at ClinicalTrials.gov (NCT05481996. Registered on August 01, 2022).

**Supplementary Information:**

The online version contains supplementary material available at 10.1186/s13063-023-07833-9.

## Background

Low back pain (LBP) is the leading cause of years lived with disability [1, 2], resulting in high rates of productivity loss worldwide [2]. One out of four adults experience chronic LBP [3] (CLBP; pain persisting for more than 12 weeks) [4] worldwide. This prevalence is 2.5 times higher in working populations [5]. The CLBP prevalence in public safety workers (e.g., police officers and firefighters) ranges from 28.7 to 76.2% [6–8], and in Brazil, LBP is the main reason for sick leave in this population [9–11].

Several factors can increase the risk of experiencing LBP, including physical (e.g., regular lifting), psychological (e.g., depression), and poor general health (e.g., sleep problems) [12]. Notably, the daily activities of police officers and firefighters inherently involve these factors. These professionals often adopt poor postures, such as prolonged standing while fighting fires or while patrolling in police cars [13], frequently engage in heavy lifting (e.g., carrying body armor, weapons, tools, and oxygen cylinders) [14, 15], experience mentally stressful situations (e.g., armed confrontations and accidents with fatalities) [13, 16, 17], and face challenges affecting their sleep quality (e.g., long work shifts) [18, 19].

The association between subjective (i.e., pain) and objective measures (i.e., endurance and strength of the trunk muscles) in individuals with CLBP [20] plays a crucial role in the understanding and management of CLBP among public safety workers (i.e., police officers and firefighters). Current evidence supports the effectiveness of interventions involving physical exercises, particularly core strengthening, in comparison to usual treatments for reducing pain and improving functional disability in CLBP patients [21, 22]. In addition, internet-based self-management programs (e.g., websites, video conferencing, smartphone apps) have gained widespread recognition for CLBP rehabilitation [23–25]. Telerehabilitation, specifically using a mobile-based (m-health) exercise program, helps overcome several potential barriers to access traditional in-person healthcare services in some police and firefighter’s institutions, including travel-related issues (distance, transit, transportation, and time consumption) and lack of time [26, 27]. This approach enables users to access healthcare services remotely, which may not be available in person within their local areas. Previous meta-analyses have shown that self-managed m-health-based programs may effectively reduce pain and disability in patients with CLBP [28, 29]. However, the effectiveness of exercise-based telerehabilitation strategies on biopsychosocial outcomes in police officers and firefighters with CLBP is not entirely established. Previous systematic review of non-conservative interventions in tactical populations (i.e., police, firefighters, and military personnel) showed that interventions using telerehabilitation approach in this population are scanty [30].

Therefore, this clinical trial will test the effectiveness of an m-health-based self-managed program based on core strengthening exercises plus health education, compared to an m-health education only, on physical and perceptual outcomes. Considering that several studies demonstrate the effectiveness of health education in the clinical outcomes of patients with CLBP, we choose for an active comparator group intending to reach the principle of equipoise. We hypothesize that the effects of an m-health self-managed program based on core strengthening exercises plus health education will be superior on co-primary and secondary outcomes, compared to education health program.

## Methods

### Protocol elaboration and registration

This study protocol follows the recommendations of the SPIRIT (Standard Protocol Items: Recommendations for Interventional Trials) 2013 Statement [31] (see checklist in Additional file 1). The SAFEBACK study is a randomized controlled trial registered on Clinical Trials (NCT05481996) on August 1, 2022, before the first participant enrolment.

### Study design

**Fig. 1:**
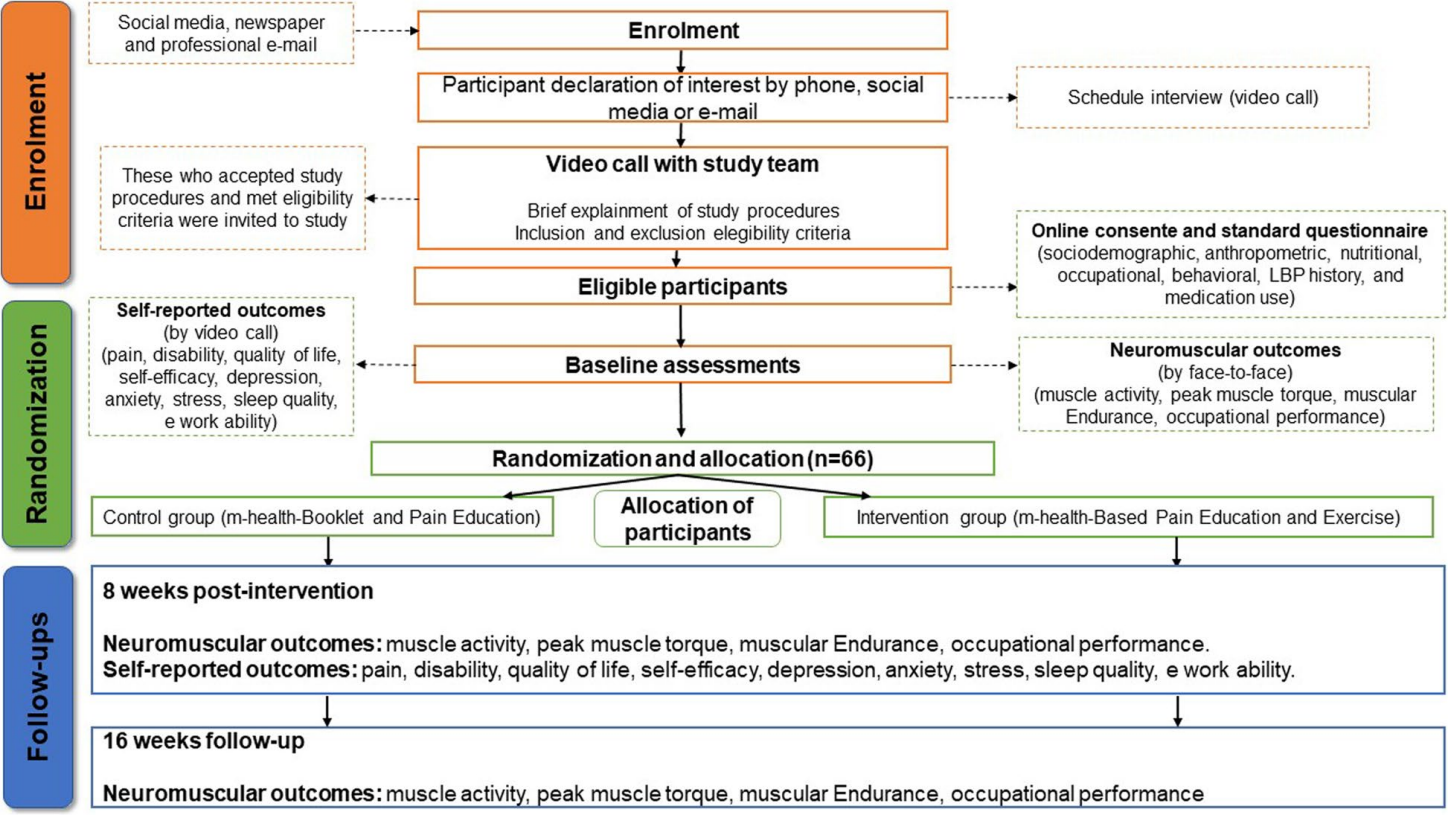
Study design

This study is a prospectively registered, parallel, two-armed, assessor-blinded, superiority randomized controlled trial. Three assessment points were performed (baseline, 8 weeks post-intervention, and 16 weeks follow-up) with probability sampling with randomization in an allocation ratio 1:1. The planned flow diagram of this trial is presented in Fig. 1.

### Study setting and eligibility criteria

This trial was conducted until August 2023 at the *Universidade Federal de Pelotas*, Pelotas, Brazil. The participants were recruited from the Rio Grande do Sul State (Brazil) public safety organizations through advertising in social media, local newspapers, and through professional e-mail of public security organizations involved in the study. Police officers and firefighters were defined as those that belong to the following public security organizations: Federal Police, Federal Highway Police, Civil Police, Military Police, and Military Firefighters. They shared exposure to a range of physical, behavioral, and mental factors known to be associated with LBP experience [13–15]. The recruitment period occurred from October 2022 to May 2023. During recruitment, we determined study eligibility based on inclusion and exclusion criteria defined as follows:

Inclusion criteria:

- Aged between 18 and 60 years
- CLBP (defined as pain lasting more than 12 weeks);
- CLBP of at least three points in a 0 to 10 Pain Numerical Rating Scale (NRS) [32];
- Having a smartphone with internet access and an e-mail account;
- Police officer (federal, federal highway, civil or military) or military firefighter working in the State of Rio Grande do Sul.

CLBP was defined as pain or discomfort between the 12th ribs and the lower gluteal folds, with or without symptoms referred to the lower limbs, lasting more than 12 weeks (chronicity), not attributed to a specific diagnosis [33].

Exclusion criteria:

- Neurological symptoms (nerve root compromise or sensation deficits);
- Spinal severe diseases (e.g., fracture, tumor, inflammatory, autoimmune, and infectious diseases);
- Severe cardiovascular and metabolic diseases (e.g., coronary heart disease, cardiac insufficiency, decompensated diabetes);
- Recent spine surgery (over the last 12 months) or scheduled to undergo surgery in the next 6 months;
- Pregnancy;
- History of physical therapy treatment for LBP or physical exercise (strength training for core muscles, Pilates, yoga) current or within the last 3 months;
- Retired;
- Having any contraindications to exercise.

We pre-screened physical activity participation at baseline using the Physical Activity Readiness Questionnaire (PAR-Q) Portuguese version [34]. The PAR-Q is a physical activity readiness questionnaire consisting of seven closed questions (yes or no). If a “YES” answer was given, the individual was advised to seek medical referral for engaging in physical activity, following the PAR-Q protocol.

### Data collection and management procedures

#### Pre-intervention assessment

After initial contact with researchers, volunteers were scheduled an interview (through a video call) with one of the assessors where the eligibility criteria for participation in the study was checked. Eligible participants received the online consent form via email (see Additional file 2). After online signature, they completed a standardized questionnaire regarding sociodemographic, anthropometric, nutritional, occupational, behavioral, LBP history, and medication use. A second video call was pre-scheduled in the eligibility assessment for data collection related to the self-reported outcomes. Afterward, a face-to-face assessment of the neuromuscular outcomes was conducted. Weight, height, and waist, hip, and abdominal circumferences were recorded in the baseline face to face assessment. Blinded assessors performed all data collection related to the co-primary and secondary outcomes at all time points. All outcomes were assessed 8 weeks after the intervention, while only the online self-reported outcomes were collected at 16 weeks.

**Fig. 2:**
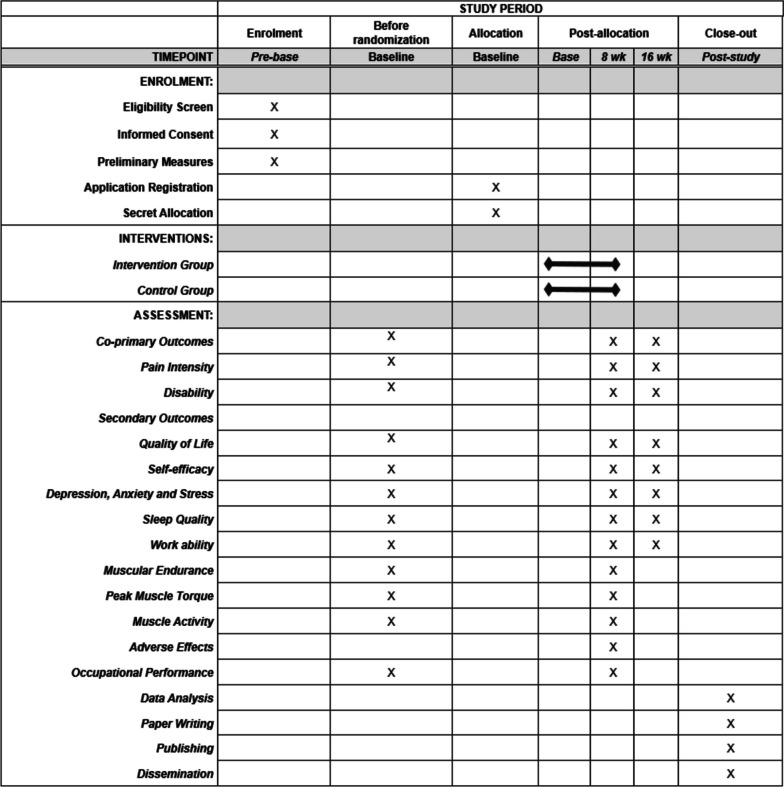
Time scheme for study conduction

The baseline, 8-week, and 16-week online assessments was conducted via video call using a personalized link on the Google Meet platform, and the self-reported data were assessed using the Google Forms platform. All data entries collected by the questionnaire were converted and coded to an Excel spreadsheet (Microsoft Corporation, Redmond, Washington) and double-checked before analysis. The time scheme for study conduction is presented in Fig. 2.

### Outcome measures

The co-primary outcomes will be pain intensity and disability at 8 weeks (post-treatment). Pain intensity was measured using the 11-point Pain NRS, a numerical scale where 0 indicates no pain and 10 indicates maximum pain intensity, presenting good levels of test–retest reliability intraclass correlation coefficient (ICC = 0.94) [35]. Disability will be measured using the 24-item Roland Morris Disability Questionnaire [36], a questionnaire that assesses normal activities of daily living, where a higher score indicates a higher level of disability and having excellent test–retest reliability (ICC = 0.94) [37].

The secondary outcomes will be as follows:

- Pain intensity at 16 weeks follow-up;
- Disability at 16 weeks follow-up;
- Health-related quality of life at all time points: measured with the WHOQOL-Pain [34], a self-reported questionnaire with 16 questions and classification of 4 facets related to the experience of chronic physical pain. Higher scores reflect better quality of life;
- Self-efficacy at all time points: measured with the Chronic Pain Self-Efficacy Scale [38], a questionnaire with 22 questions classified into three domains and have internal consistency values ranging from satisfactory to excellent, both for the domains (*α* Cronbach = 0.76–0.92) and for the total scale (*α* Cronbach = 0.94) [39]. Each domain score ranges from 10 to 100. Higher scores reflect a greater sense of self-efficacy;
- Depression, Anxiety, and Stress at all time points: measured by the Depression Anxiety Stress Scale [40], a self-reported scale with 21 questions, 7 for depression, 7 for anxiety, and 7 for stress with a construct validity and internal consistency that ranges from strong to excellent (*r* = 0.74–0.86; *α* Cronbach = 0.86 0.0.92, respectively) [40]. Each question ranges from 0 to 3 according to the patient’s response. Higher scores indicate worst results;
- Sleep quality at all time points: measured by the Pittsburgh Sleep Quality Index [41], a self-reported scale with 19 questions that assess seven components of sleep and has a high degree of internal consistency (*α* Cronbach = 0.82) [41]. Each question ranges from 0 to 3 according to the patient’s response;
- Work ability at all time points: measured by a single-item question, “Are you working at a physically less demanding job now because of your back and/or leg pain?” [42, 43];
- Isometric muscular endurance of the trunk extensor at 8 weeks (post-treatment): measured by the modified Biering-Sorensen test [44]. This test indicates the time the participant can remain with the trunk in a horizontal and straight position. This test has moderate test–retest reliability (ICC = 0.87) among individuals with CLBP [44]. Longer time indicates better isometric resistance scores;
- Isometric muscular endurance of the trunk flexors at 8 weeks (post-treatment): measured by a test that consists of remaining in isometry as long as possible in a sitting position with 45° hip flexion [45]. The longer the time indicates better isometric resistance scores;
- Maximum isometric strength of the trunk extensor and flexor muscles at 8 weeks (post-treatment): maximum voluntary isometric contraction (MVIC) was measured by a calibrated load cell (Miotec®, Porto Alegre, Brasil) of 200 kgf. For the MVIC test of the trunk flexors muscles, the participants were positioned in a sitting position on top of the stretcher, with knees extended, legs fixed to the stretcher with two traction straps, hips flexed and resting on the backrest of the stretcher adjusted at 30° the surface of the stretcher, and arms crossed in front of the torso. For the MVIC test of the trunk extensor muscles, the participants were positioned in ventral decubitus, with knees extended and legs fixed to the stretcher with traction straps and elbows flexed with hands superimposed on the head. For both tests, the load cell was attached to an adapted stretcher, aligned perpendicularly to the torso, passing through the stretcher’s aperture. Chains connected the load cell to a traction belt positioned at rib height (xiphoid process). Participants performed three 5-s attempts for each position, with a 2-min interval between each attempt. During the tests, participants were verbally encouraged to apply maximum force as quickly as possible. The cell load values (kgf) were transformed into Newton (N) and multiplied by the distance (m) between the hip joint (greater trochanter of the femur) and the point of attachment of the traction belt to calculate peak torque in N.m;
- Muscular activation of spine flexor and extensor muscles at 8 weeks (post-treatment): simultaneously with the MVIC collection, the neuromuscular activation of the trunk flexor (rectus abdominis) and extensors (erector spinae—longissimus) muscles was measured using the surface electromyography technique (sEMG). A Miotool device (Miotec®, Porto Alegre, Brazil) was used with 1 cm Ag/AgCl electrodes (Meditrace®, Mansfield, Canada) and an interelectrode distance of 30 mm. The sEMG signal was collected from the right side of the participant’s body. During the assessments, the recommendations of skin preparation, asepsis, and fixation of the SENIAM electrodes were observed [46]. The electrodes’ location on the erector spinae was carried out according to SENIAM guidelines (i.e., two finger widths laterally from the spinal process of L1). In the rectus abdominis muscle, the electrodes were positioned 3 cm laterally to the body midline on the muscle belly between the sternum and the umbilicus [47]. A reference electrode was placed on the participants right clavicle. Maps were made to help reposition the electrodes after the intervention (8 weeks) by marking anatomical points and signs on the skin in transparent plastic.
- Occupational performance at 8 weeks (post-treatment): measured by a task that simulates a victim rescue. The participant will drag a dummy (weight = 60 kg) away from the start line, around a cone placed at the 13 m mark, and return to the start line. The longer the time indicates worse occupational performance.

Eligible participants were randomly allocated to the intervention or control group with a 1:1 simple allocation using a computer-generated randomization sequence created by a blinded investigator via a random function in Excel software for Windows. After randomization, an independent researcher emailed each participant the login and password to access the specific app version to which they were allocated (intervention or control). Afterward, an assistant researcher inserted them into numbered, sealed, and opaque envelopes.

### Blinding

Due to the intervention’s characteristics, blinding of participants and intervention providers was not possible. The outcome assessors were blinded to the treatment groups. Participants are asked to omit their assigned group and not discuss their interventions during the outcome assessments. In unblinding cases, the principal investigator was notified with the participant ID, date, and reasons for unblinding.

### Interventions

Intervention descriptions followed the TIDieR (Template for the Intervention Description and Replication) checklist recommendations for telehealth interventions used in clinical trials [48].

#### Intervention group: m-health-based pain education and exercise

Patients allocated to the experimental group received a login and password for individual access to the smartphone app (www.mysafebackapp.web.app/). Before the intervention begins, participants received a yoga mat, an elastic band, and a link to a tutorial video showing how to use the app functions. The app content for this group included three components: (1) a physical exercise program of 8 weeks, (2) weekly messages, and (3) an online booklet.

The exercise component (app “training” function) included 8 weeks of training, with two sessions per week (non-consecutive days) of core strengthening exercises. The exercise program was developed by exercise professionals with at least 5 years of clinical experience, specialists in chronic pain treatment, and who used exercise-based treatment. The training periodization followed the progression of pre-established protocols for CLBP treatment reported in the literature (see training periodization in Additional file 3) [49–52]. The exercises performed in each stage are presented in Additional file 4.

The app provided animated Graphics Interchange Format (GIFs), descriptions, and audio of how to perform each exercise. The number of repetitions or time under tension for each exercise were prescribed with a range of target zones (e.g., 10–12 repetitions, 20–30 s). For those who cannot reach these zones, they were asked to indicate (on the app) that they could not perform the complete exercise. Exercise sessions were carried out remotely and self-managed by the participant (i.e., the participants were responsible for carrying out the exercises). The content of the training sessions was made available (visible in the app version) each week of intervention training.

The message component from the app provided 8 messages (one per week), with the contents taken from the online booklet. Messages included information about the benefits of exercise, motivation, and positive messages about coping with pain.

The online booklet contained general information about self-management of chronic pain, including pain education, advice on healthy lifestyle and sleeping habits, and promotion of exercises. The “Contact” function from the app provided participants a direct communication with the researcher to answer questions about the app, its features, and information about possible app errors. In addition, to improve training adherence, the app provided a count and progression bar (%) of the number of completed training days. Adherence was defined as the absolute and relative frequency of the number of sessions performed.

#### Control group: m-health-booklet and pain education

Patients allocated to the control group received a login and password for individual access to the smartphone app designed for the study. The app content for this group included two components: (1) an online booklet and (2) weekly messages. The online booklet contained general information about self-management of CLBP, including pain education, advice on healthy lifestyle and sleeping habits, and promotion of physical activities based on the “Back Book” (see Additional file 5).

The message component provided 8 messages (one per week), with their contents taken from the online booklet. Messages included information about the benefits of exercise, motivation, and positive messages about coping with pain. Before intervention begins, participants received a yoga mat, an elastic band, and a link to a video tutorial on how to use the app. To increase adherence to the intervention, after the study is concluded, the training function available in the app will be offered to the control group participants if the intervention group proved to be more effective than the control group.

### Strategies for trial retention

Participants allocated to both groups received, in week 4, WhatsApp messages to reinforce the use of the app. There are prohibited concomitant interventions including any systematic or supervised physical exercise to strengthen core (e. g., Pilates, Yoga). Any exercise should be reported by the participants and documented for future discussions.

### Statistical methods

#### Sample size calculation

Sample size estimation was performed a priori through G-Power’s statistical software (version 3) [53]. A sample size calculation determined that a minimum of 66 individuals would be required in the present study. Data used included 80% power to detect a between-group difference of 1.5 points in a 0 to 10 Pain NRS [54, 55], with an estimated standard deviation (SD) of 2.0 points [56], and a between-group difference of 4.0 points in a 1 to 24 points RMDQ [57], with an estimated SD of 4.9 points [58] and alpha error (two-tailed) of 5%. The estimated sample size would also allow for a loss to follow-up rate of up to 15% observed in studies involving similar populations (firefighters and military) and forms of intervention provision (i.e., app) [56, 59].

#### Data analysis

The baseline characteristics of the participants were calculated using descriptive statistics. The between-group differences and 95% confidence interval (95%IC) for the post-treatment outcomes at 8 weeks and 16 weeks follow-up were calculated using linear mixed models using interaction terms of treatment group versus time. An intention-to-treat approach will be used in all statistical analyses. Per-protocol analyzes will also be performed, including only participants from the experimental group arm who adhere to the intervention (i.e., having performed at least 12 of the 16 training sessions). For the intention-to-treat analyses, the incomplete data (i.e., missing values) were estimated using multiple data imputation techniques through statistical software. All statistical analyzes will be performed using the SPSS statistical software (Statistical Package Social Science), version 20.0 for Windows (IBM corporation, Somers, New York, USA).

#### Monitoring

##### Data monitoring and auditing

The study does not have a data monitoring committee either planned auditing trial conduct due to limited resources. We believe this committee would not be mandatory due to the characteristics of interventions and outcomes despite its high value for the overall trial quality. Interim analyses will not be conducted in this trial.

##### Harms, ancillary, and post-trial care

The adverse events were collected by weekly recording the number of adverse events throughout the intervention period. A closed-ended question with two options (yes or no) sent by the app at the end of each week: “During the last week of the study, did you present any different symptoms or worsening of the initial condition?”. Also, in case of a positive answer, a second closed question was asked: “Do you think this symptom or worsening of the initial condition was generated by the treatment provided in this study?”.

At least two researchers discuss all adverse events: the main researcher (EFM), the study manager (CLA), and medical consultants and experts if necessary. If the treatment is causing the participant to deteriorate, a video call is offered with one of the study physiotherapists to evaluate the issue or suggest discontinuation of the treatment for the participant.

In the case of the proven effectiveness of the exercises provided by the app in managing CLBP, access to the effective app version for participants in the control group after the end of the study period was provided.

##### Dissemination policy

After completing the study, we aim to disseminate the results to as many stakeholders as possible. First, participants will receive their reports with their measurements and interpretations in language adapted to the lay public’s understanding. The main findings, whether positive, negative, or inconclusive, will be disclosed directly or at an event aimed at managers of public security organizations through the exhibition/presentation of a structured summary (infographic) and submitted to a peer-reviewed international journal for possible publication. All information regarding the full protocol, participant-level dataset, and statistical code will be available from the corresponding author upon reasonable request.

## Discussion

This randomized control trial will investigate a new model of care to treat a highly prevalent and disabling problem in public safety workers, such as police and firefighters. We hypothesize that the m-health-based telerehabilitation program will benefit public safety workers with CLBP by reducing pain intensity and improving function and psychological, behavioral, and neuromuscular outcomes compared to a mobile minimal intervention approach (booklet).

The app is based on relatively simple interventions (i.e., remote-based exercise and pain education). This app was designed to be simple and have easy implementation in the worker health settings of public safety institutions. Some public safety institutions do not have, or only provide, in-person hospital care in a single location. Thus, the app developed will be able to expand care and potentially change the lives of millions of police officers and firefighters with CLBP that have limited access to face-to-face care.

In case of our hypothesis being confirmed, we can contribute to the discussion of a new treatment modality (i.e., m-health-based telerehabilitation program) for this population. The results may have practical applications to public security organizations that implement this therapeutic modality in CLBP treatment and may imply the reduction of absenteeism and presenteeism of these professionals, with a positive impact on the service provided to society.

## Trial status

The recruitment period for this study occurred from October 2022 to May 2023. This is the first version of the manuscript and is accompanied by a description of existing amendments (see Additional file 6). Any modifications to the protocol that may impact the conduct of the study, the potential benefit of the patient, or patient safety, including study procedures or significant administrative aspects, will require a formal amendment to the protocol. Such amendments will be notified to the research ethics committee and amended in the study registry. Furthermore, it is worth noting that any potential protocol bias resulting from these amendments will be transparently addressed in the original article that will report the study’s results.

### Supplementary Information

**Additional file 1. **Reporting checklist for protocol of a clinical trial.

**Additional file 2. **Consent form.

**Additional file 3. **Training periodization for the experimental group.

**Additional file 4. **Illustrations of exercises for the experimental group.

**Additional file 5. **The back book.

**Additional file 6. **Amendment’s chronology.

## Data Availability

All data will be used only for analysis of the present study and will be protected from unnecessary exposure. According to the Research Ethics Committee of Physical Education School, the authors and patients will sign the consent form. The paper information will be kept in binders and handled only by the researchers responsible for the study in locations accessible only to researchers responsible for data analysis. The online information will be in a database maintained on a server, to which only the researcher responsible for the project will have access. All information will be published confidentially without the name of the participants exposed. All data will be available for review and confirmation of data analysis when requested by a review process for publication of the article in indexed scientific journals or presentations at scientific events. The data will be available from the corresponding author upon reasonable request.

## Abbreviations

App: Application
CLBP: Chronic low back pain
m-health: Mobile health
TIDieR: Template for the Intervention Description and Replication
SPIRIT: Standard Protocol Items: Recommendations for Interventional Trials
