# Supplementary material for: Effectiveness of m-health-based core strengthening exercise and health education for public safety workers with chronic non-specific low back pain: study protocol for a superiority randomized controlled trial (SAFEBACK)
Source: Trials. 2023 Dec 1;24:780. doi: 10.1186/s13063-023-07833-9 (PMC10693081; doi:10.1186/s13063-023-07833-9)
Supplement: Supplementary file 2 — Additional file 2. Consent form. [file 13063_2023_7833_MOESM2_ESM.docx]

**Consent form**

I agree to participate in the study “Effectiveness of an exercise-based telerehabilitation program for police officers and firefighters with chronic non-specific low back pain: a randomized clinical trial”. I am aware that I am being invited to participate voluntarily.

**PROCEDURES:**

I have been informed that the overall objective will be to develop and test the effectiveness of a self-managed physical exercise program delivered via a smartphone application in police officers and firefighters with chronic non-specific low back pain through a randomized clinical trial on pain, disability, self-efficacy, depression, anxiety, stress, quality of life, and sleep and how pain impacts work; neuromuscular variables of muscular strength and endurance, muscular electromyographic activation and adverse events. The study will take eight weeks and after finished there will be a follow-up of eight weeks. The results will be kept confidential and will only be used for research purposes.

I am aware that my participation will involve an initial screening, in order to determine whether I attend the eligibility criteria to participate in the study. After confirming the eligibility, an evaluation will be conducted via videoconference, using scales and questionnaires that measure: low back pain intensity, pain-related disability, self-efficacy for chronic pain, depression, anxiety and stress, quality of life and quality of sleep (duration of 30 minutes). Additionally, in person measures will be performed, such as isometric muscular endurance and maximum strength tests on an adapted stretcher. Furthermore, during these tests (maximum strength) the muscular electrical activity of the trunk muscles will also be measured using the surface electromyography technique (total duration of the in-person session will be 30 minutes). For the neuromuscular measures the surface of the skin at the site will be shaved, followed by cleaning with cotton soaked in alcohol to position the electrodes. A scraping will be done with individual disposable blades and the procedure will be performed by an experienced appraiser. The researcher will use procedural gloves during all collections and materials will be discarded in the appropriate trash for each type of material.

After the assessments, you will receive (via email) a login and password to access the application (link sent via email) in which you will be randomly assigned to receive one of the interventions: 1) Exercises via the application; or 2) Control. Regardless of the group you are selected in, you will receive a basic treatment, consisting on guidance from an online booklet, accessed via the app, made specifically for people who suffer from low back pain and you can clarify all your doubts on a weekly basis, with a researcher (there are several studies that demonstrate the effectiveness of this booklet in patients with low back pain), through an application function (contact). The treatment will last 16 sessions (two weekly sessions lasting ~25-30 minutes). After the end of treatment (eight weeks) and after four months of follow-up after randomization, you will be re-evaluated by the same researcher who initially evaluated you. Furthermore, at the end of the 8 weeks of study, the group that only receives the online booklet will be granted access to the application's exercise function, if observed benefit.

**RISKS AND POSSIBLE REACTIONS:**

You may feel embarrassed in to answer any question asked in the virtual environment (videoconference). You can stop participating in the research, without suffering any harm as a result. Still, it is possible that you feel some discomforts during and after face-to-face assessments. The researchers involved in this study will take all necessary precautions in order that these possible discomforts are kept to a minimum. Additionally, if you are allocated to the exercises via app, there may be some exercises that will challenge your strength, fitness, balance and coordination. You may experience muscle pain within one or even two days of participating in a session of exercise, however, this is completely normal and expected. The risk of injury is inherent in any exercise program; however, an exercise science professional will help you aiming to minimize this risk, solving doubts about the exercises you will perform, in order to increase the safety. If necessary, during the in-person assessment, you will be attended by the research team, considering that some team members are trained for Basic Life Support, as well as being staffed by physiotherapists, exercise science professionals and occupational therapists, in order to provide full time assistance. If necessary, you will be taken to the Emergency Room at the local hospital. If any discomfort is reported, during and after the research, the assigned professionals will take all necessary measures for the situation. If you suffer harm as a result of your participation in the study, you have the right to compensation at any time.

**BENEFITS:**

Among the possible benefits of your participation in the study, we highlight the fact that your participation will contribute to the possible identification of the effectiveness of interventions that reduce burden of chronic low back pain in police officers and firefighters. As a consequence, the results of this research may serve as possible evidence for the treatment of chronic low back pain within of public security corporations, since, if the intervention proven to be effective, the number of sick leave for treatment and the costs of managing chronic low back pain, within the scope of these public security organizations, may be reduced.

**VOLUNTARY PARTICIPATION:**

As I have already been told, my participation in this study will be voluntary and I can stop it at any time.

**EXPENSES:**

I will not have to pay for any of the procedures, nor will I receive financial compensations.

**CONFIDENTIALITY:**

I understand that my identity will remain confidential during all stages of the study.

**CONSENT:**

I received clear explanations about the study, all recorded in this consent form. The study investigators have responded and will respond, in any stage of the study, to all my questions. Therefore, I agree to participate in the study. By signing this consent form, which means checking the “YES” option in the question below, a copy of it will be automatically sent to my email address provided in the online questionnaire. This Consent Form, together with the data collected, will be archived at the institution responsible for the research for a minimum period of five years.

**DECLARATION OF RESPONSIBILITY OF THE INVESTIGATOR:**

I explained the nature, objectives, risks and benefits of this study. I made myself available for questions and answered them in its entirety. The participant understood my explanation and accepted, without impositions, sign this consent. I am committed to use the data and material collected for the publication of reports and scientific articles referring to this research. If the participant has any concerns or questions about the ethics of the research, he can contact the ESEF/UFPel Research Ethics Committee – 625 Luís de Camões, 96055-630 - Pelotas/RS; Phone: (53) 3284-4332 or by contacting researchers, either for a fee or via WhatsApp messages.
