# Supplementary material for: Effectiveness of m-health-based core strengthening exercise and health education for public safety workers with chronic non-specific low back pain: study protocol for a superiority randomized controlled trial (SAFEBACK)
Source: Trials. 2023 Dec 1;24:780. doi: 10.1186/s13063-023-07833-9 (PMC10693081; doi:10.1186/s13063-023-07833-9)
Supplement: Supplementary file 3 — Additional file 3. Training periodization for the experimental group. [file 13063_2023_7833_MOESM3_ESM.docx]

**Additional file 3 –** Training periodization for the experimental group.

| **Part** | | **Week** | **Exercise** | | **Set** | **Number of repetitions (rep)/Time under tension (s)** | **Rest between exercises** |
| --- | --- | --- | --- | --- | --- | --- | --- |
| **WARM-UP**  **(mobilities)** | | **1-8** | 1. Single-leg hip flexion | | 1 | 10 rep per leg | 30 s |
|  |  |  | 2. Unilateral hip internal rotation | | 1 | 10 rep per leg | 30 s |
|  |  |  | 3. Trunk extension | | 1 | 10 rep | 30 s |
|  |  |  | 4. Cat-camel | | 1 | 10 rep | 30 s |
|  |  |  | 5. Unilateral trunk internal rotation | | 1 | 10 rep per arm | - |
|  |  | Total time = 5 min | | | | | |
| **Stage** | | **Week** | **Exercise** | | **Set** | **Number of repetitions (rep)/Time under tension (s)** | **Rest between exercises** |
| **INITIAL** | **MAIN** | **1ª** | Isometric front plank with hand support | | 2 | 20-30 s | 45 s |
|  |  |  | Dynamic bridge | | 2 | 12-15 rep | 45 s |
|  |  |  | Isometric side plank with knee support | | 2 | 10-15 s for each side | 45 s |
|  |  |  | Superman (isometric) | | 2 | 20-30 s | 45 s |
|  |  |  | *Bird-dog* | | 2 | 12-15 rep for each side | 45 s |
|  |  | Total time = ~13 min | | | | | |
|  |  | **2ª** | Isometric front plank with forearm support | | 2 | 20-30 s | 45 s |
|  |  |  | Dynamic bridge | | 2 | 12-15 rep | 45 s |
|  |  |  | Isometric side plank with knee support | | 2 | 10-15 s for each side | 45 s |
|  |  |  | Superman (isometric) | | 2 | 20-30 s | 45 s |
|  |  |  | *Bird-dog* | | 2 | 12-15 rep. for each side | 45 s |
|  |  | Total time = ~13 min | | | | | |
| **INTERMEDIATE** | **MAIN** | **3ª** | Single leg isometric front plank | | 3 | 15-20 s per leg | 45 s |
|  |  |  | Single leg dynamic bridge | | 3 | 10-12 rep per leg | 45 s |
|  |  |  | Isometric side plank with foot support | | 3 | 15-20 s for each side | 45 s |
|  |  |  | Superman with upper limb movement | | 3 | 10-12 rep | 45 s |
|  |  |  | *Bird-dog* with isometric peak in extension (2 s) | | 3 | 10-12 rep for each side | 45 s |
|  |  | Total time = ~24 min | | | | | |
|  |  | **4ª** | Single leg isometric front plank | | 3 | 15-20 s per leg | 45 s |
|  |  |  | Single leg dynamic bridge | | 3 | 10-12 rep per leg | 45 s |
|  |  |  | Isometric side plank with foot support | | 3 | 15-20 s for each side | 45 s |
|  |  |  | Superman with upper limb movement | | 3 | 10-12 rep | 45 s |
|  |  |  | *Bird-dog* with isometric peak in extension (2 s) | | 3 | 10-12 rep for each side | 45 s |
|  |  | Total time = ~24 min | | | | | |
|  |  | **5ª** | Single leg isometric front plank | | 3 | 15-20 s per leg | 45 s |
|  |  |  | Single leg dynamic bridge with heel support | | 3 | 10-12 rep per leg | 45 s |
|  |  |  | Isometric side plank with foot support | | 3 | 15-20 s for each side | 45 s |
|  |  |  | Superman with upper limb movement | | 3 | 10-12 rep | 45 s |
|  |  |  | Bird-dog with isometric peak in extension (2 s) | | 3 | 12-15 rep. for each side | 45 s |
|  |  | Total time = ~24 min | | | | | |
| **ADVANCED** | **MAIN** | **6ª** | Isometric front plank with diagonal support | | 3 | 15-20 s for each side | 45 s |
|  |  |  | Single leg dynamic bridge with heel support | | 3 | 10-12 rep per leg | 45 s |
|  |  |  | Side plank with one foot support | | 3 | 15-20 s for each side | 45 s |
|  |  |  | Superman (dynamic) | | 3 | 10-12 rep | 45 s |
|  |  |  | *Bird-dog* with elastic band resistance | | 3 | 10-12 rep. for each side | 45 s |
|  |  | Total time = ~24 min | | | | | |
|  |  | **7ª** | Isometric front plank with diagonal support | | 3 | 20-30 s for each side | 45 s |
|  |  |  | Single leg dynamic bridge with heel support | | 3 | 10-12 rep per leg | 45 s |
|  |  |  | Dynamic trunk rotation with elastic band | | 3 | 10-12 rep for each side | 45 s |
|  |  |  | Superman (dynamic) | | 3 | 10-12 rep | 45 s |
|  |  |  | Bird-dog with elastic band resistance | | 3 | 10-12 rep for each side | 45 s |
|  |  | Total time = ~24 min | | | | | |
|  |  | **8ª** | Isometric front plank with diagonal support | | 3 | 20-30 s for each side | 45 s |
|  |  |  | Single leg dynamic bridge with heel support | | 3 | 10-12 rep per leg | 45 s |
|  |  |  | Dynamic trunk rotation with elastic band | | 3 | 10-12 rep for each side | 45 s |
|  |  |  | Superman (dynamic) | | 3 | 10-12 rep | 45 s |
|  |  |  | Bird-dog with elastic band resistance | | 3 | 10-12 rep for each side | 45 s |
|  |  | Total time = ~24 min | | | | | |
| **Part** | | **Week** | **Exercise** | | **Set** | **Number of repetitions (rep)/Time under tension (s)** | **Rest between exercises** |
| **CALM DOWN (stretching)** | | **1-8** | | 1. Sit on the heel | 2 | 20 s | - |
|  |  |  |  | 2. Knee flexors | 2 | 12 rep per leg | - |
|  |  |  |  | 3. Glutes | 2 | 20 s per leg | - |
|  |  | Total time = 3 min | | | | | |
