# Supplementary material for: Effectiveness of m-health-based core strengthening exercise and health education for public safety workers with chronic non-specific low back pain: study protocol for a superiority randomized controlled trial (SAFEBACK)
Source: Trials. 2023 Dec 1;24:780. doi: 10.1186/s13063-023-07833-9 (PMC10693081; doi:10.1186/s13063-023-07833-9)
Supplement: Supplementary file 4 — Additional file 4. Illustrations of exercises for the experimental group. [file 13063_2023_7833_MOESM4_ESM.docx]

**Additional file 4 -** Illustrations of exercises for the experimental group.

| **Warm-up (Mobilities)** | | | | |
| --- | --- | --- | --- | --- |
| **Single-leg flexion** | **Initial position:**  **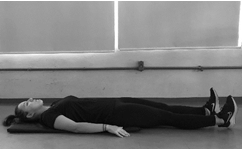** 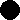 | **Final position:**  **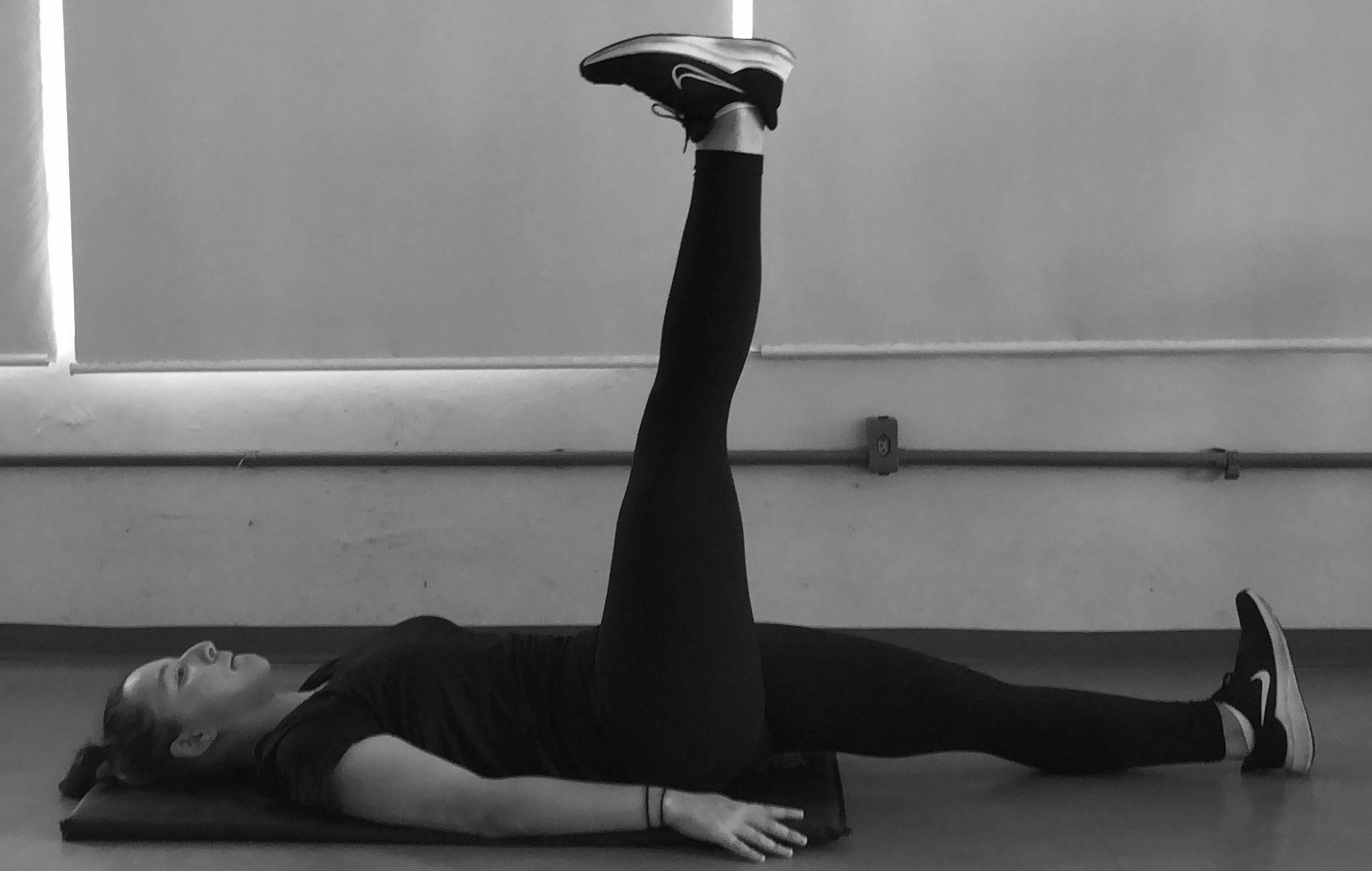** 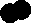 | | |
| **Unilateral hip internal rotation** | **Initial position:**  **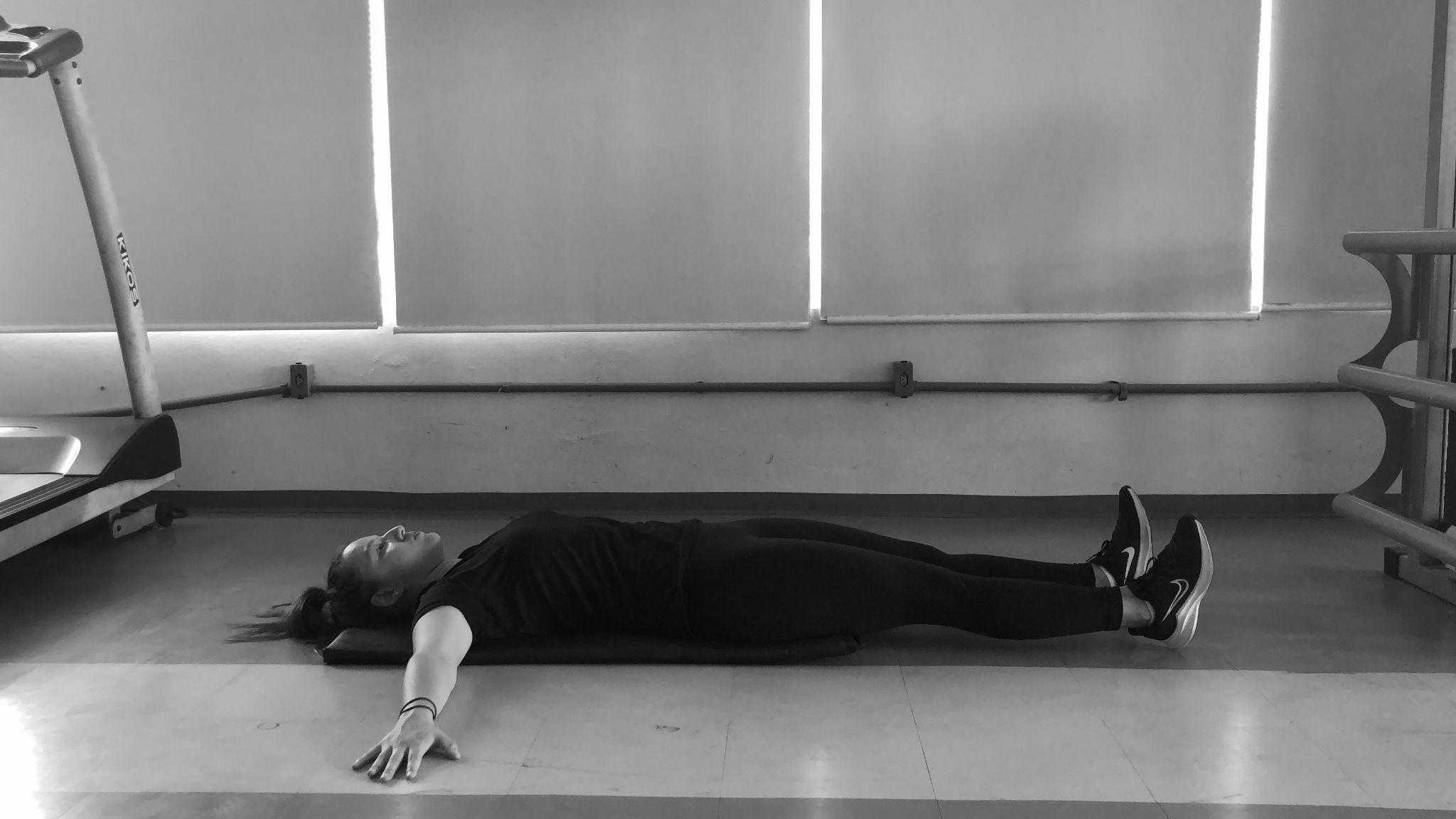** 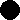 | **Transition position:**  **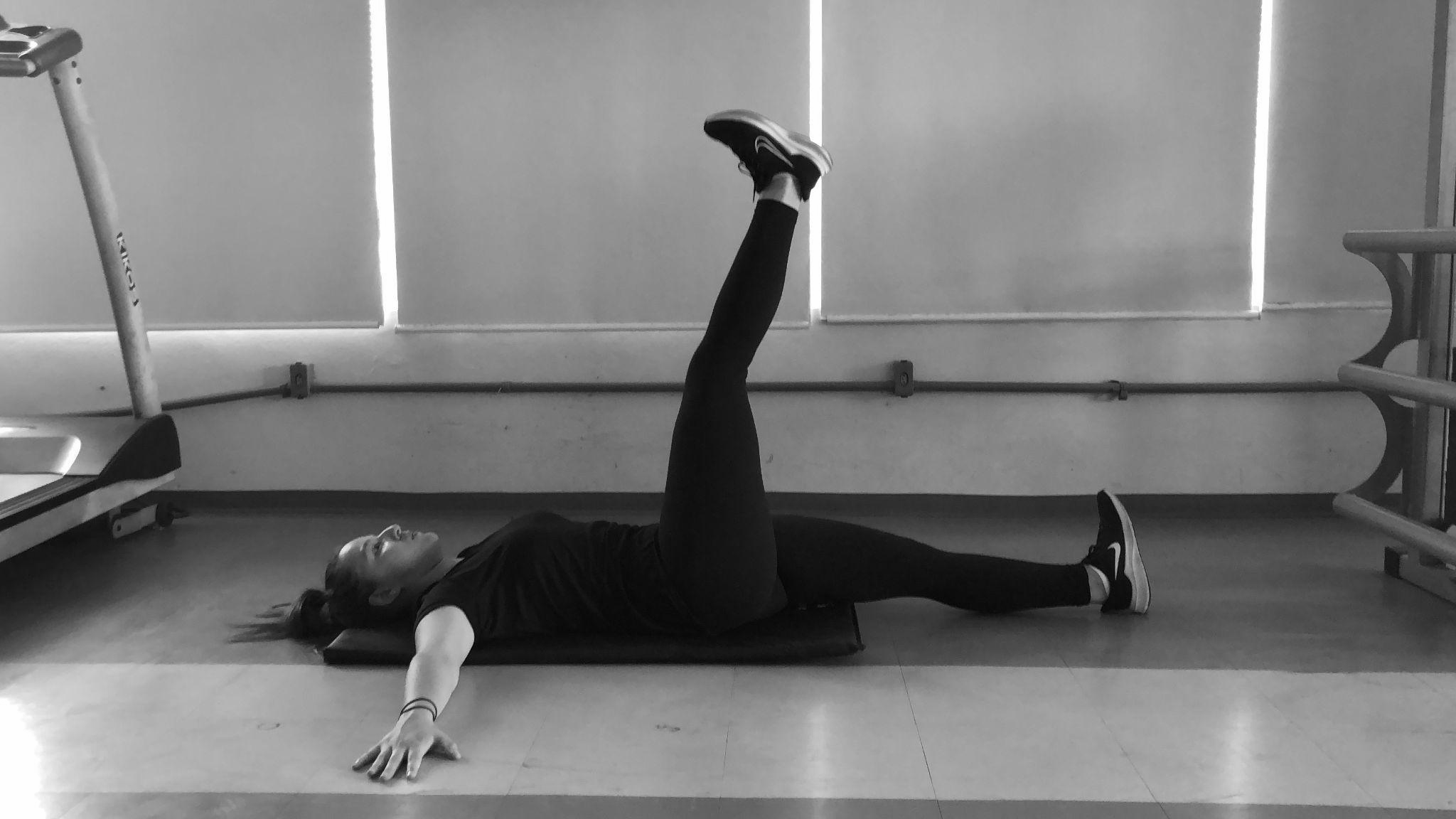** 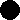 | | **Final position:**  **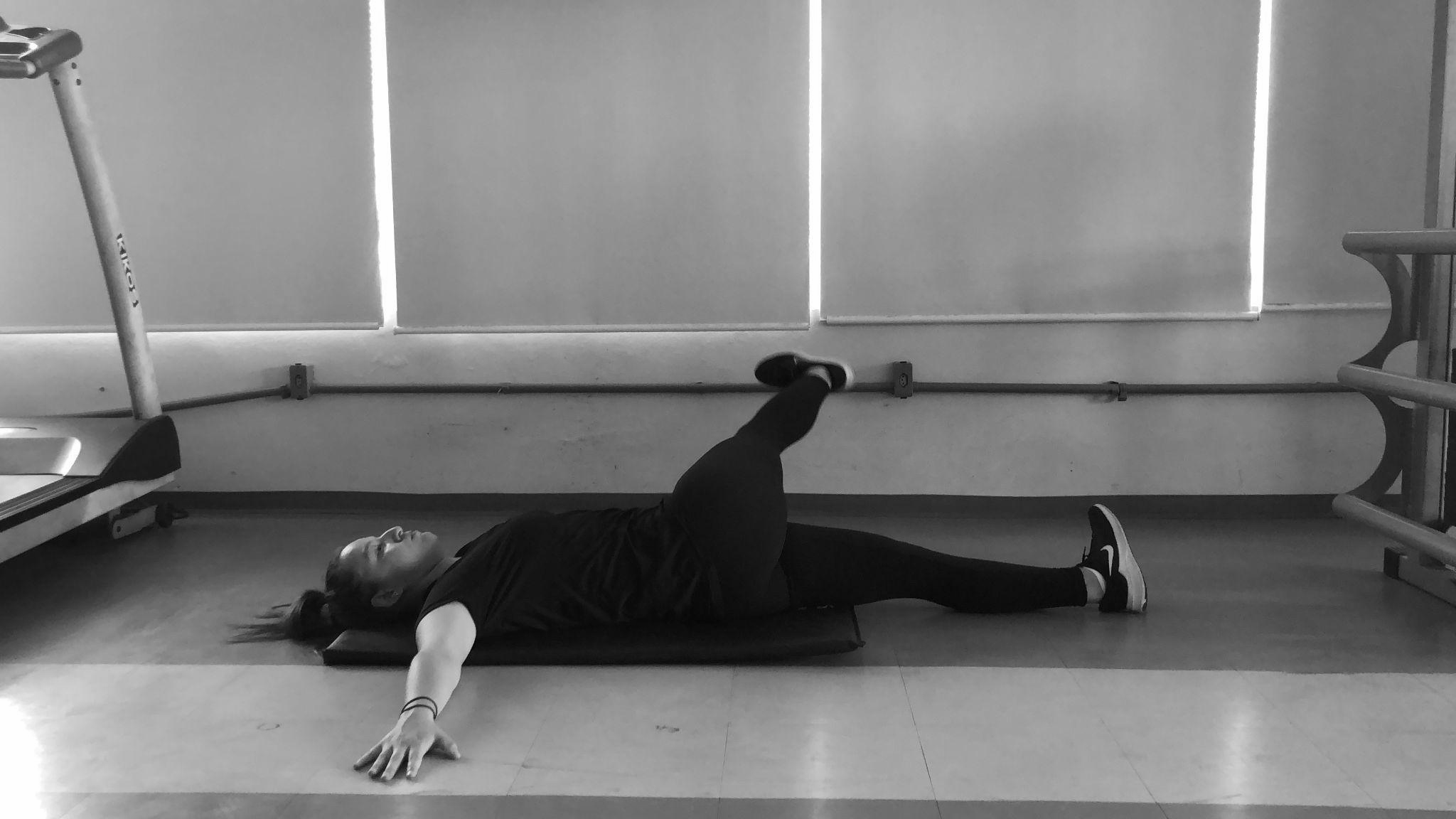** 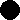 |
| **Trunk extension** | **Initial position:**  **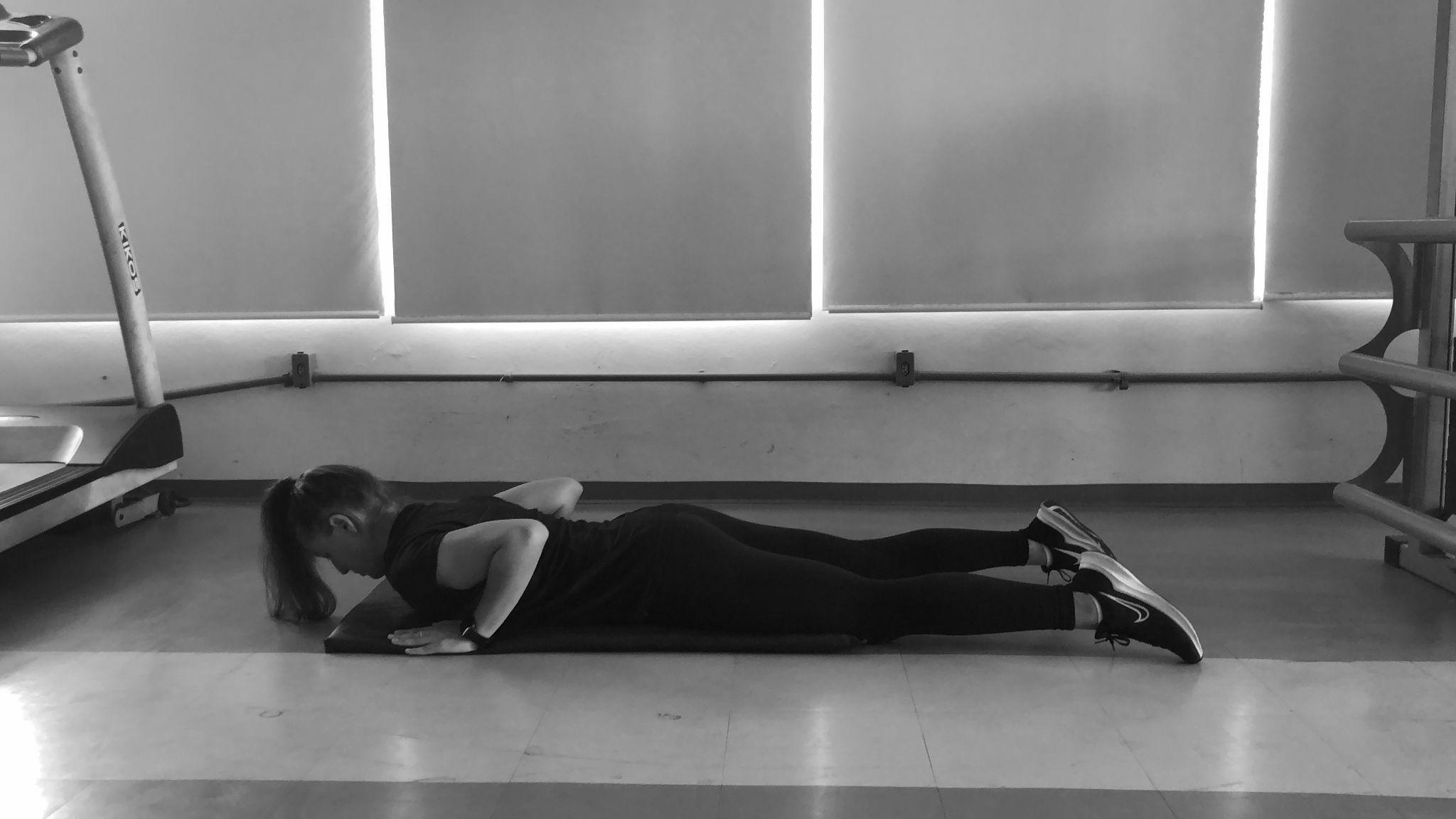** 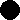 | **Final position:**  **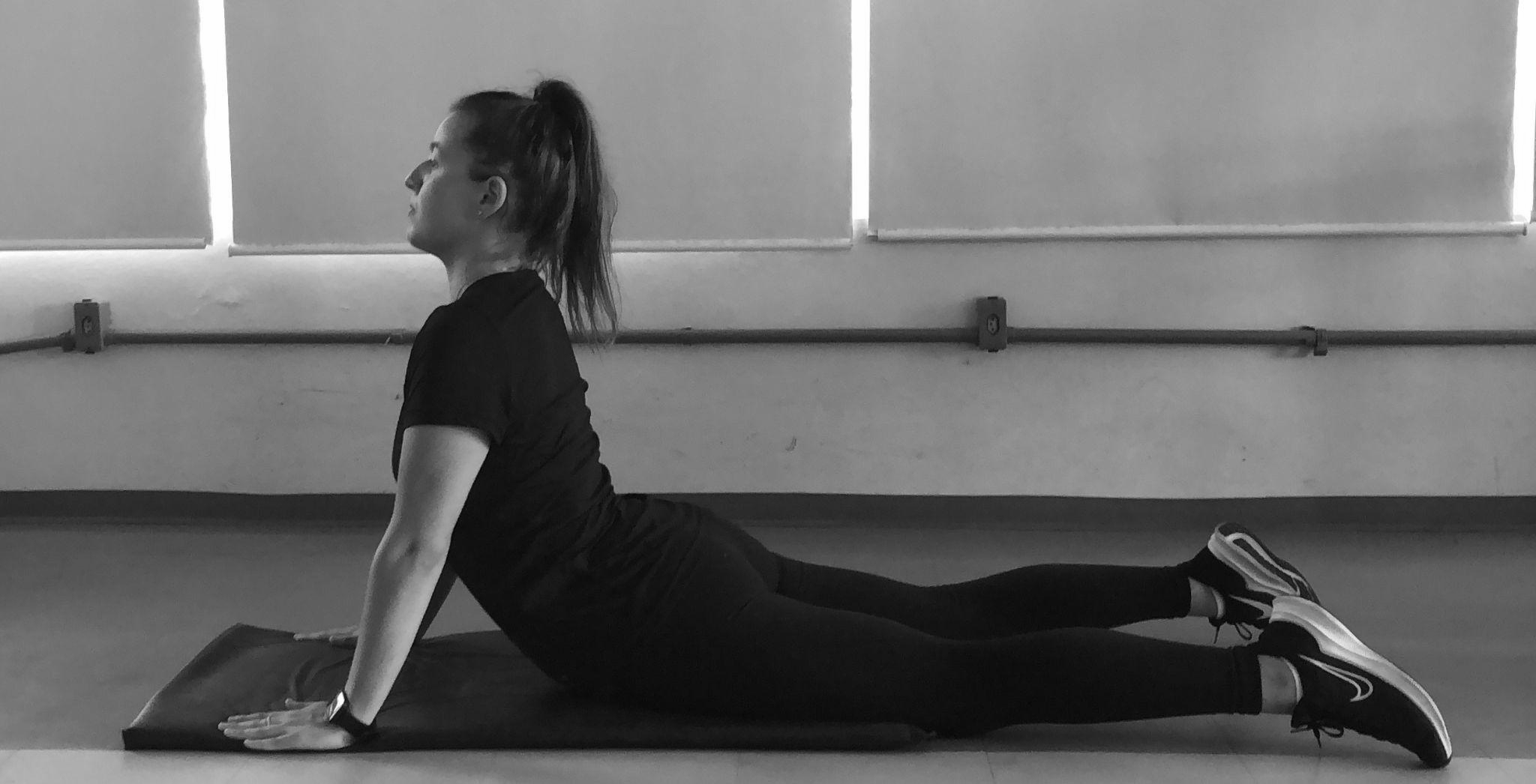** 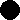 | | |
| **Cat-camel** | **Initial position:**  **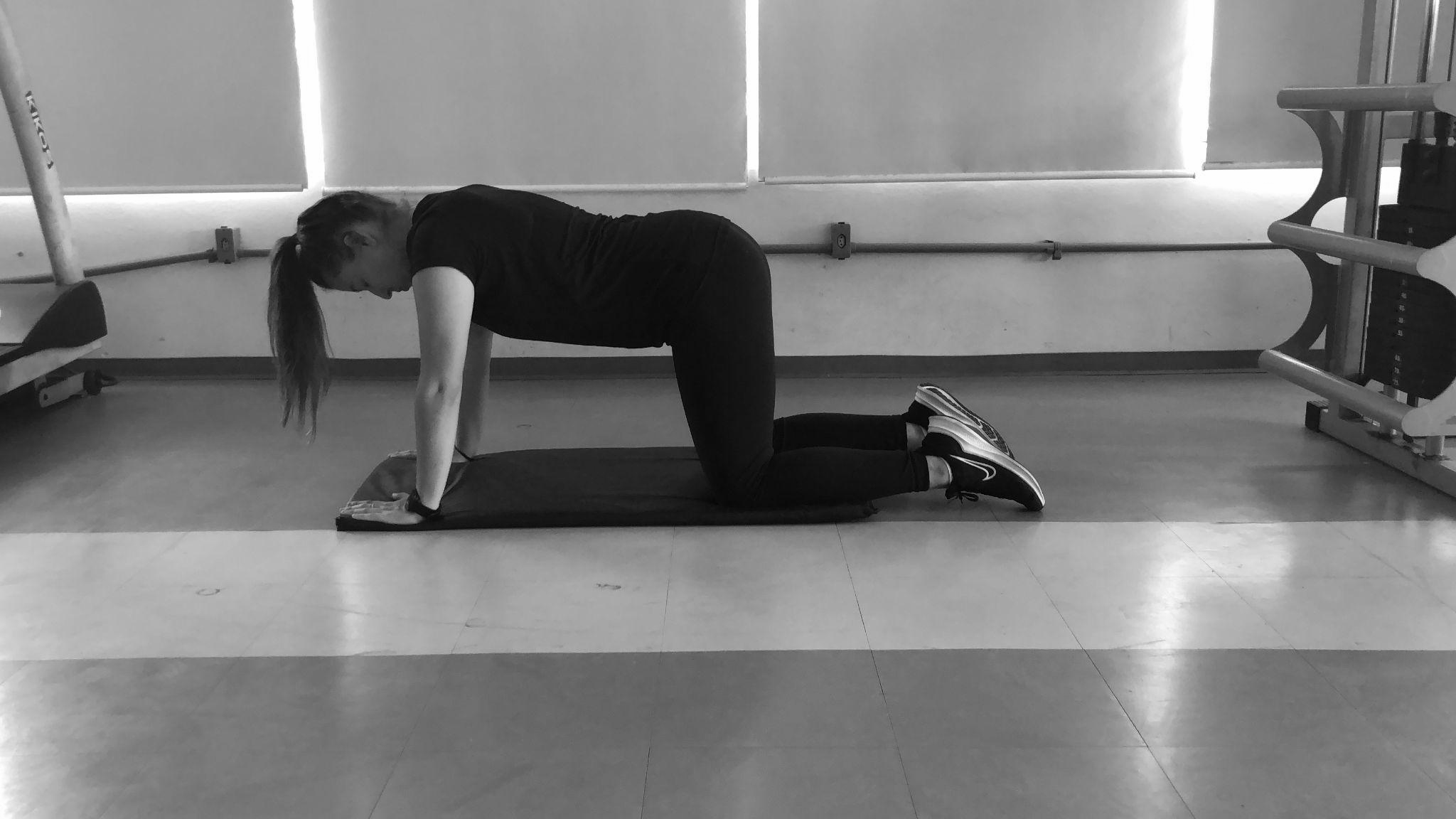** 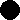 | **Final position:**  **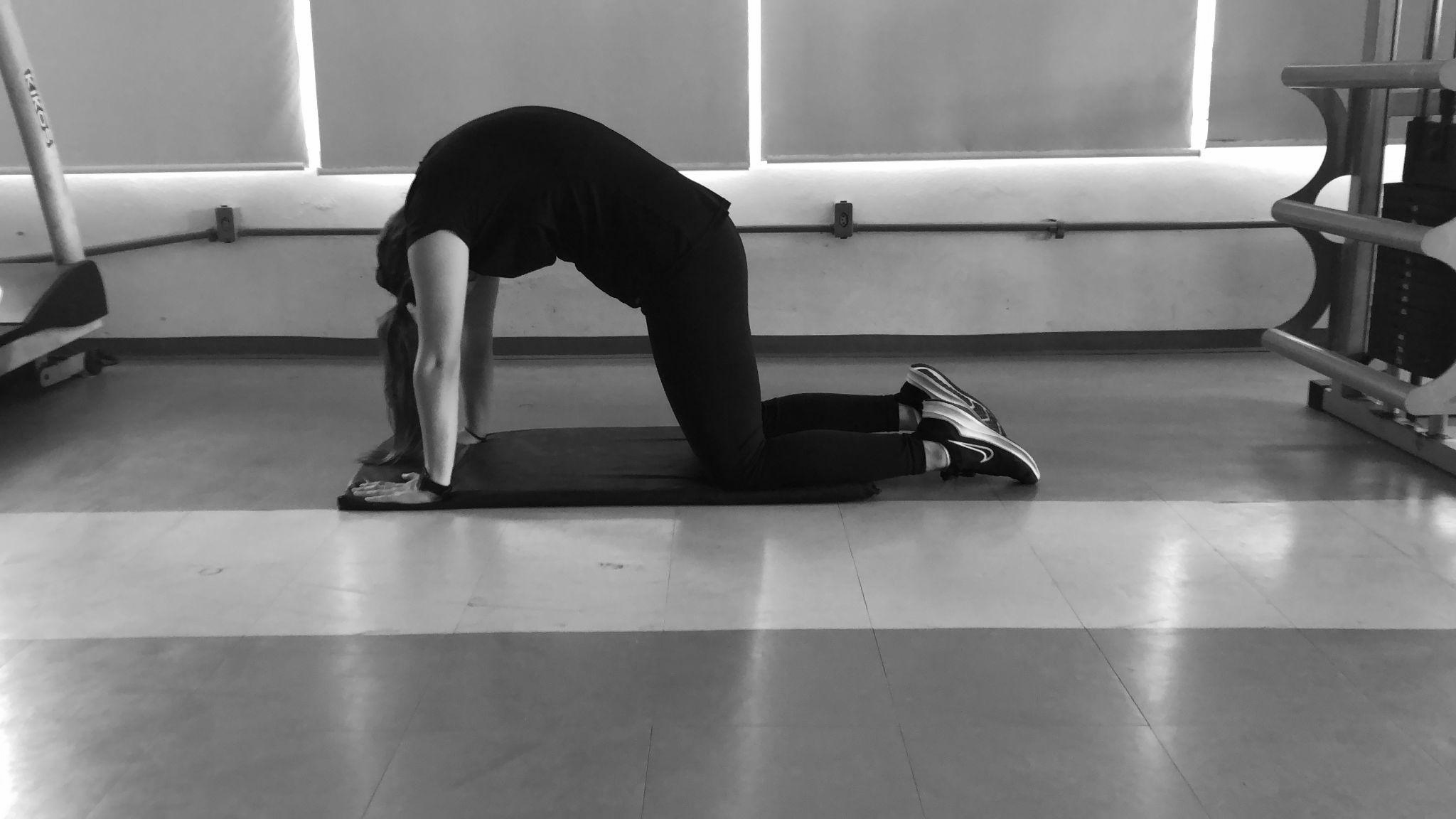** | | |
| **Unilateral trunk internal rotation** | **Initial position:**  **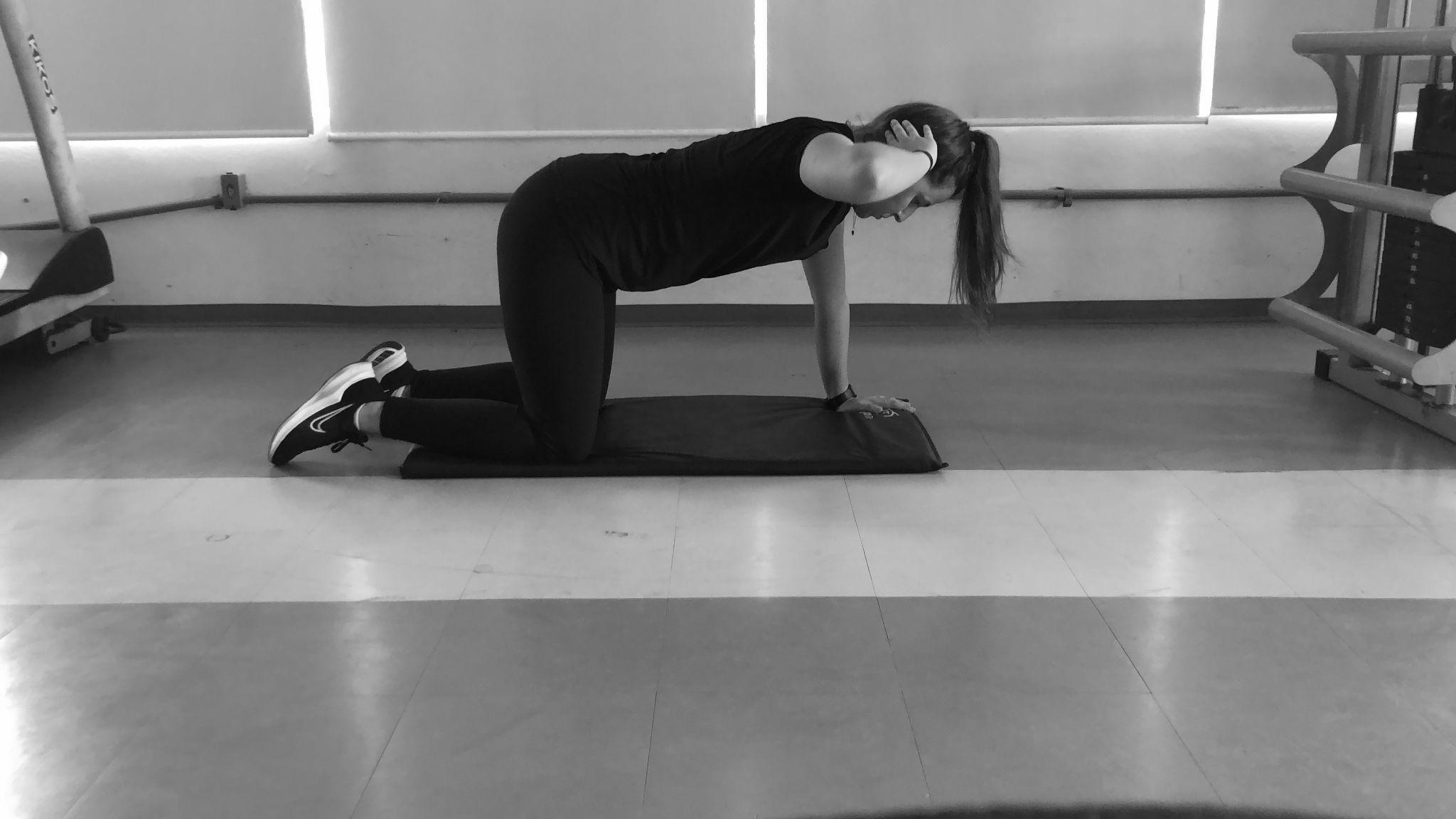** 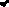 | **Transition position:**  **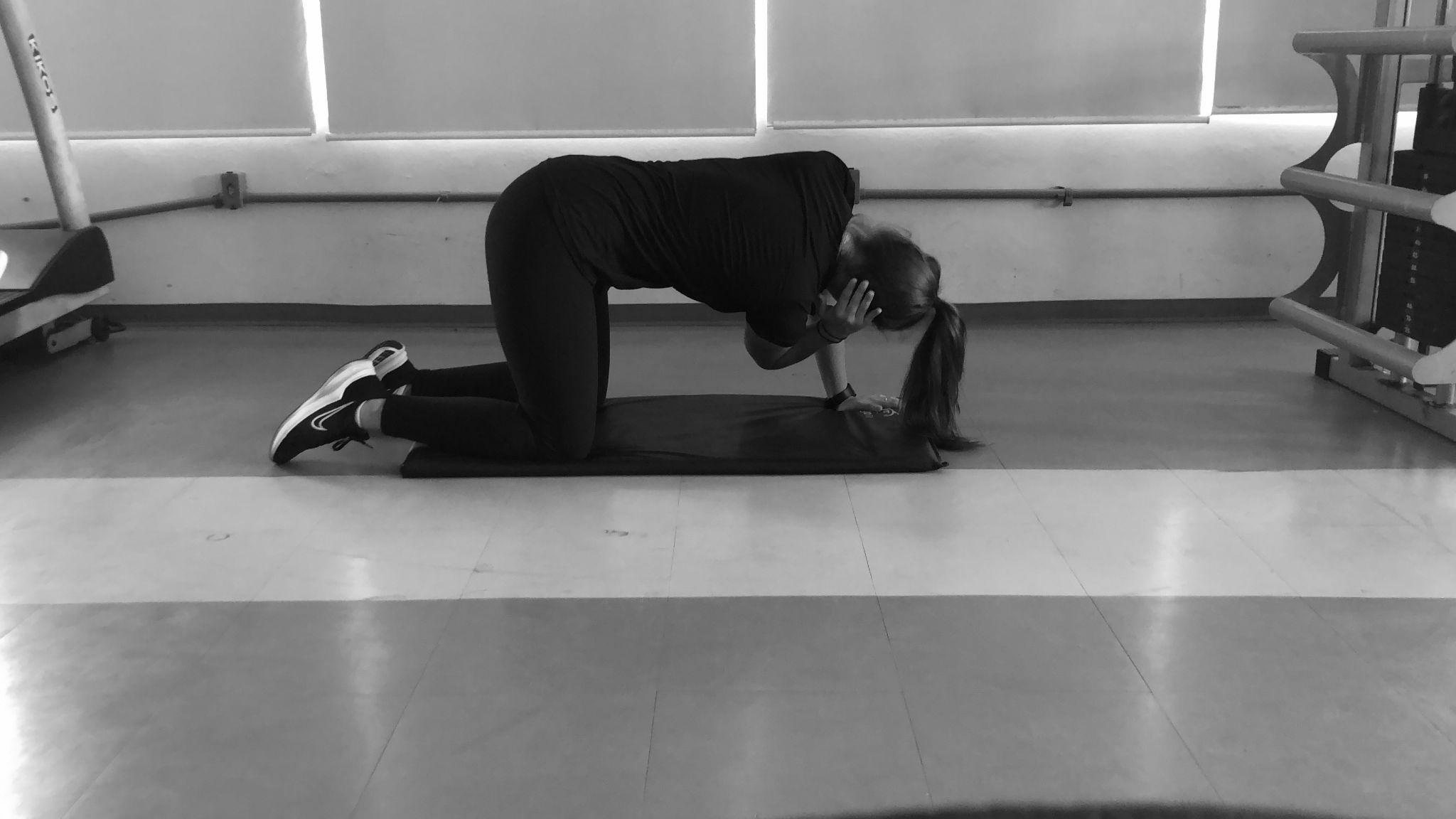** | **Final position:**  **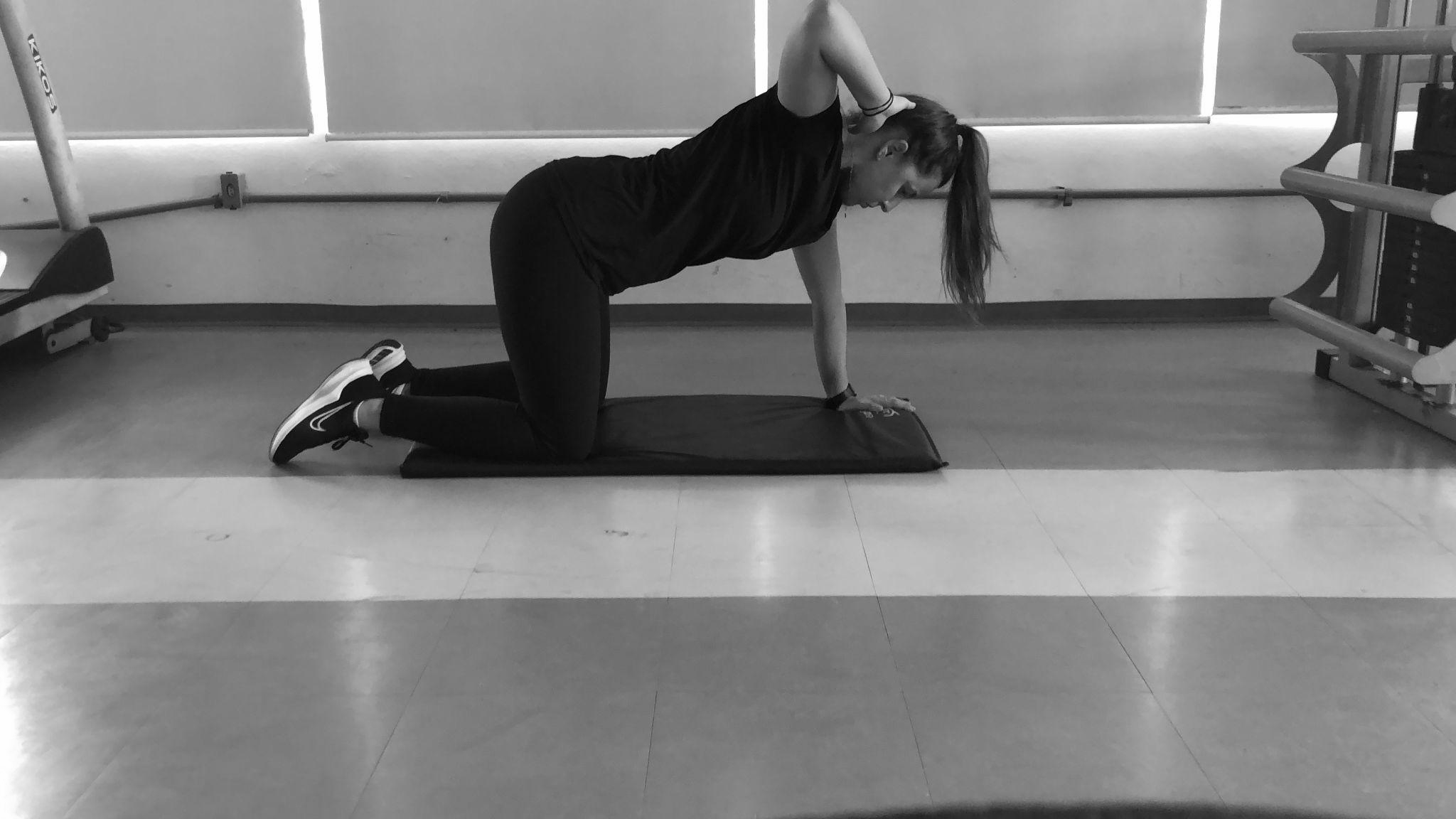** 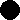 | |
| **Main part (Initial stage)**  **Weeks 1 and 2** | | | | |
| **Isometric front plank with hand support** | **Initial position:**  **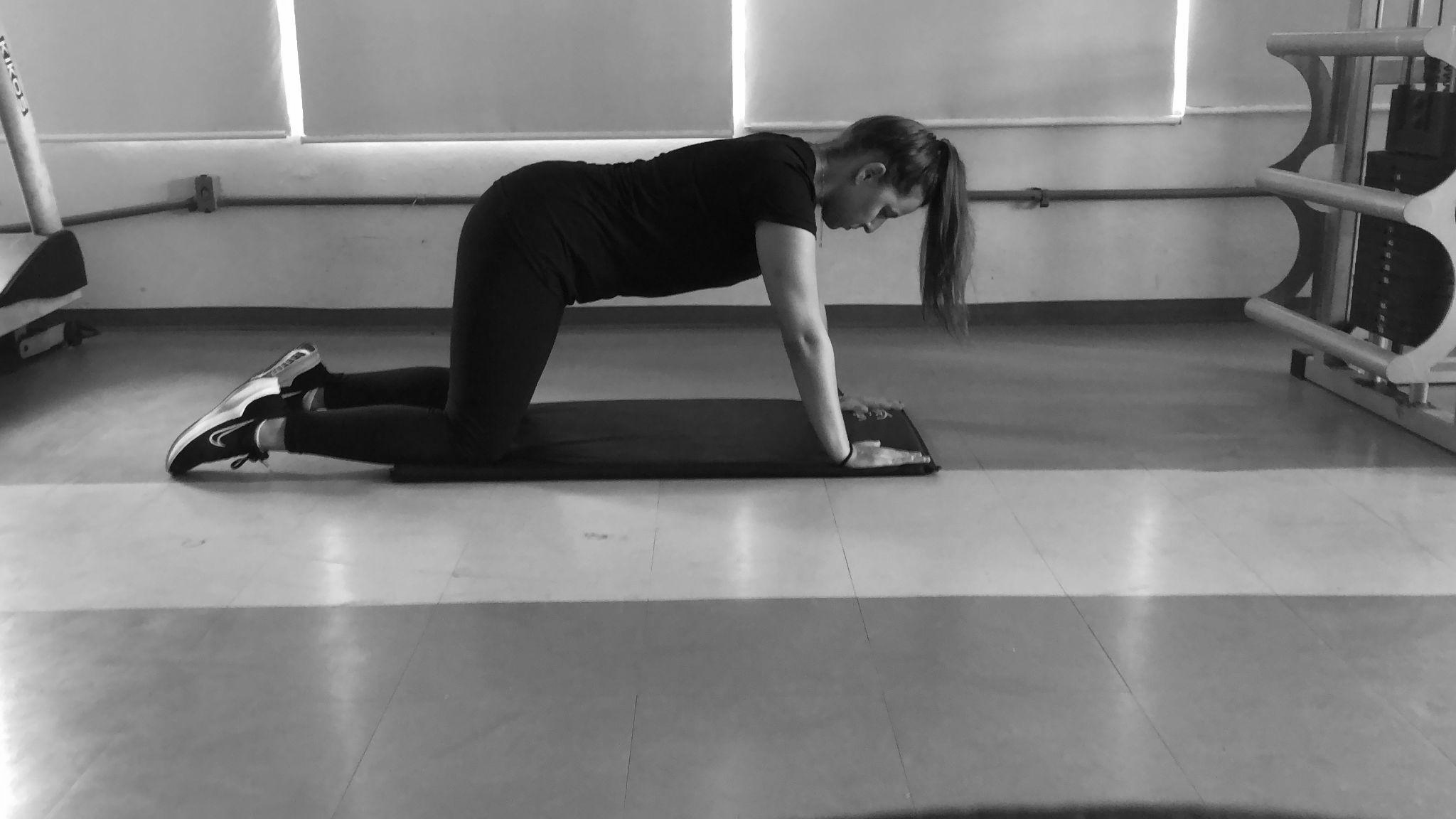** 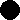 | **Final position:**  **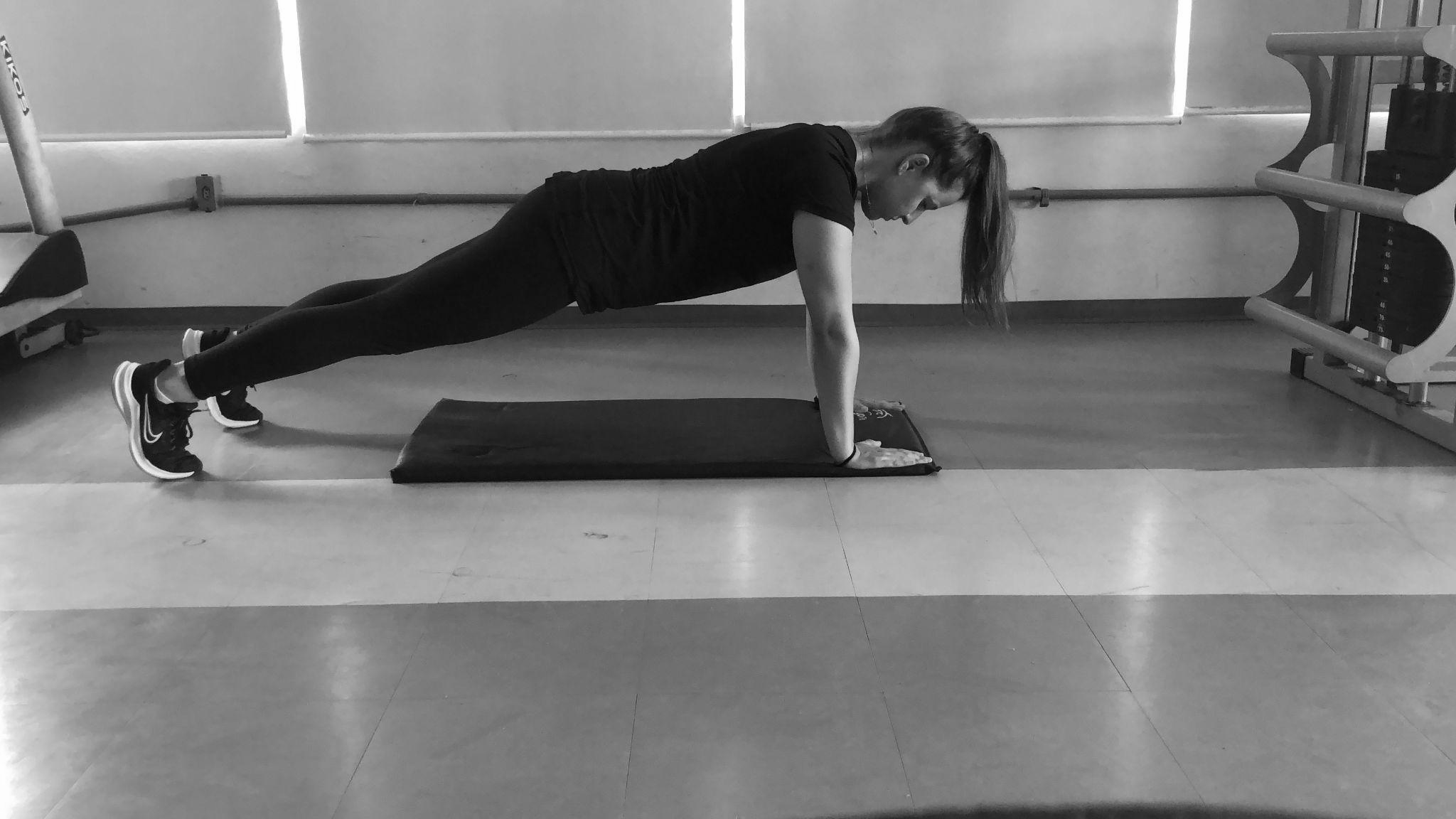** 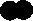 | | |
| **Dynamic bridge** | **Initial position:**  **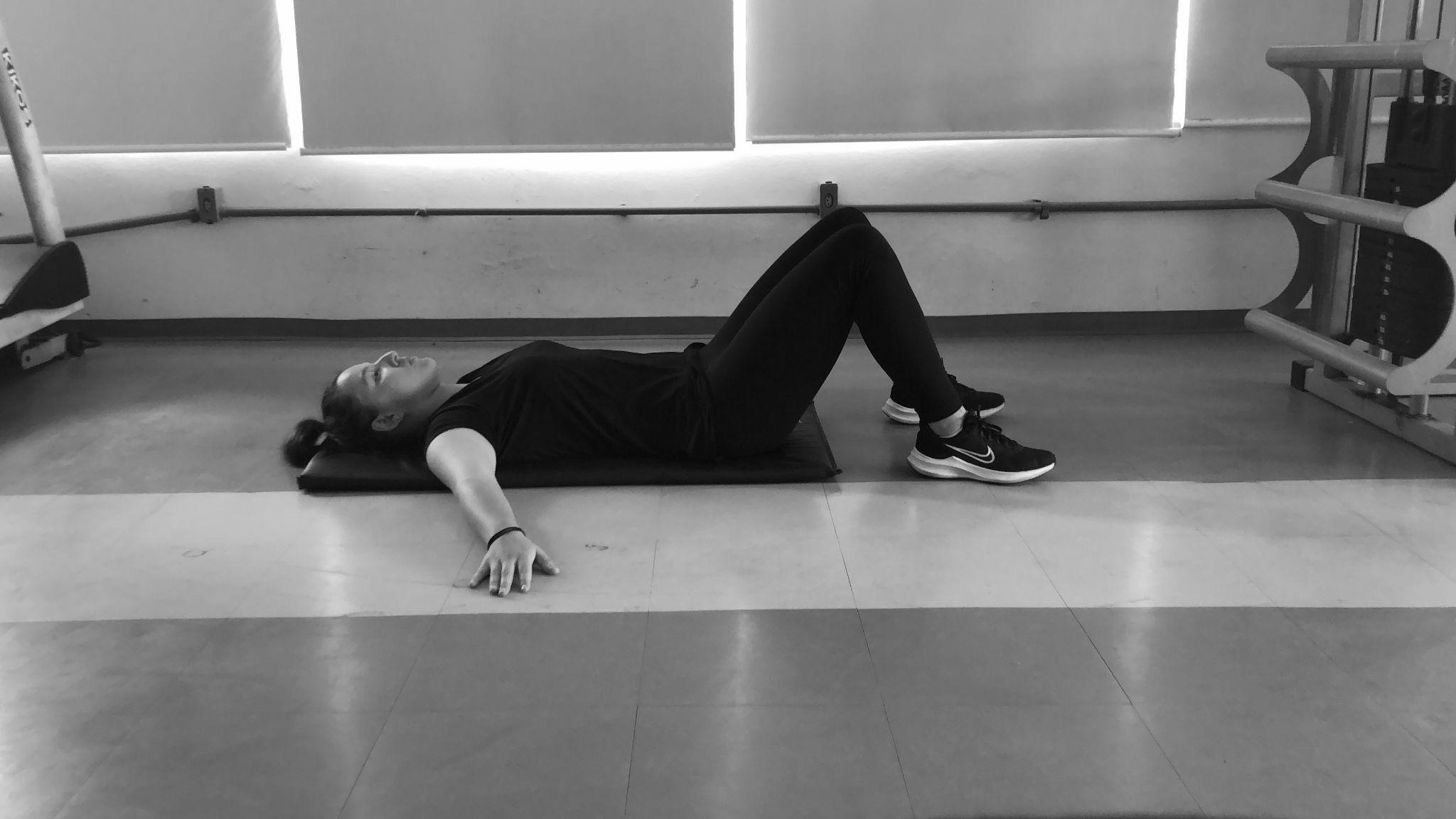** 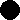 | **Final position:**  **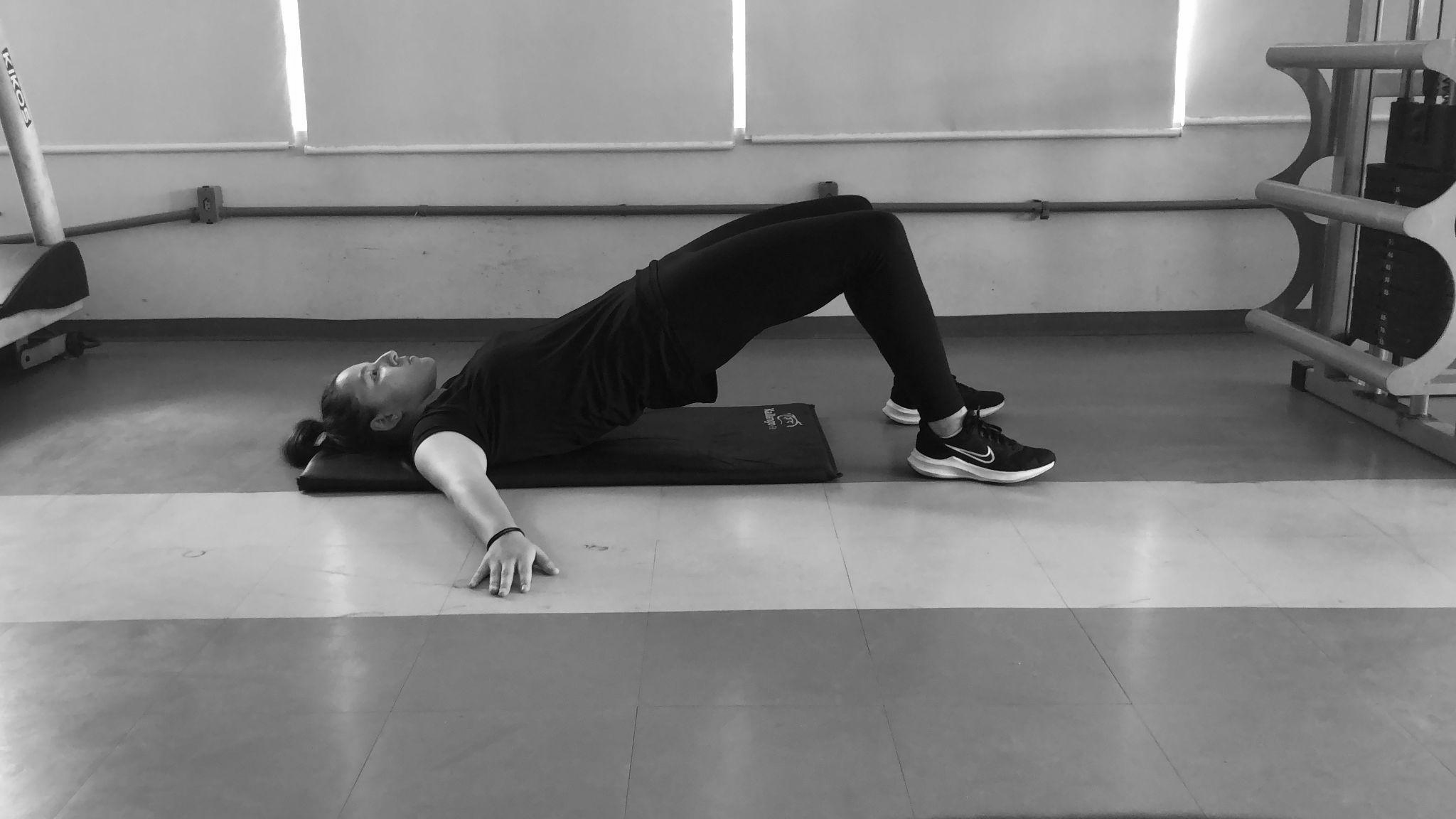** 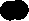 | | |
| **Isometric side plank with knee support** | **Initial position:**  **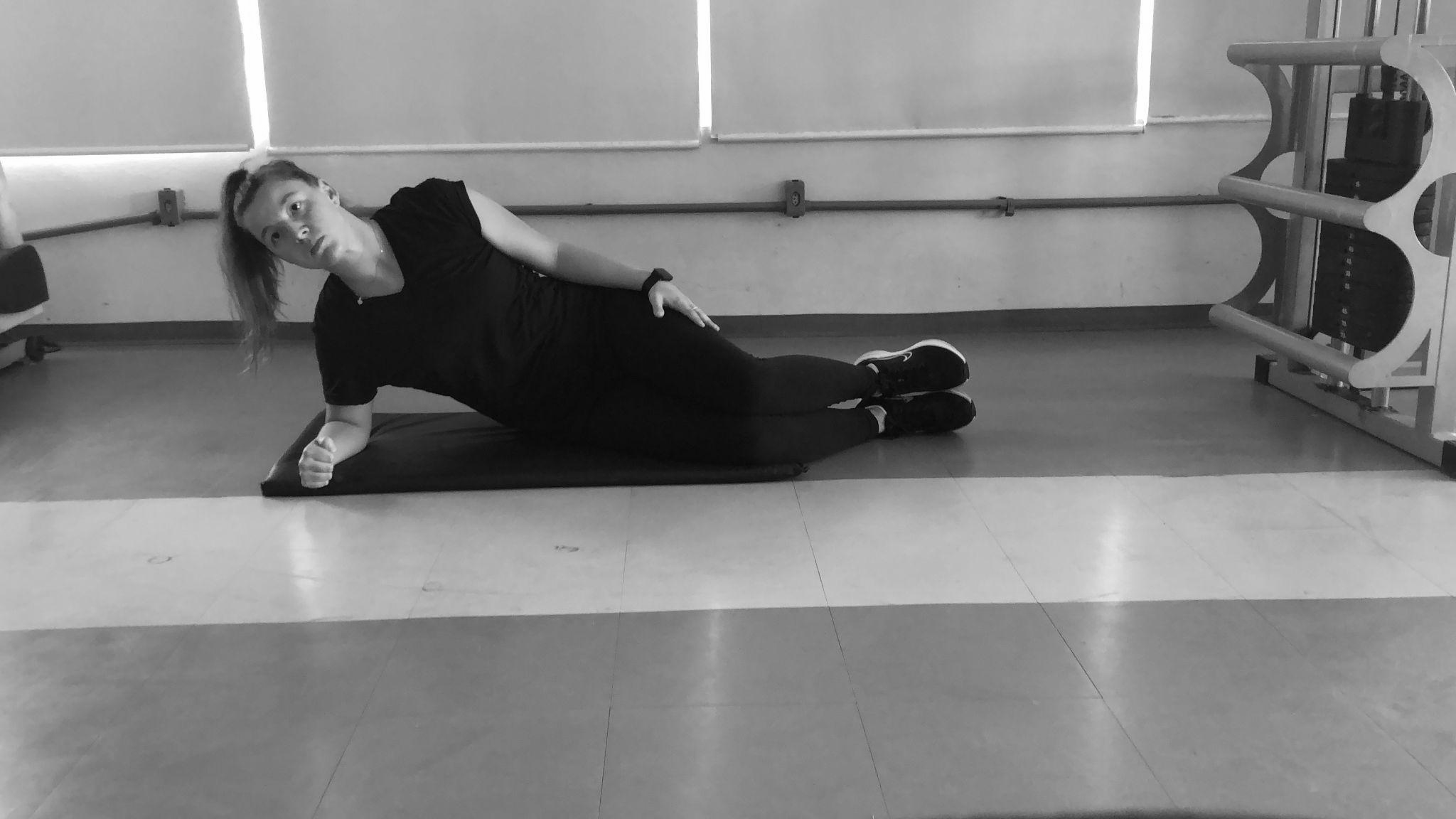** 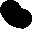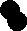 | **Final position:**  **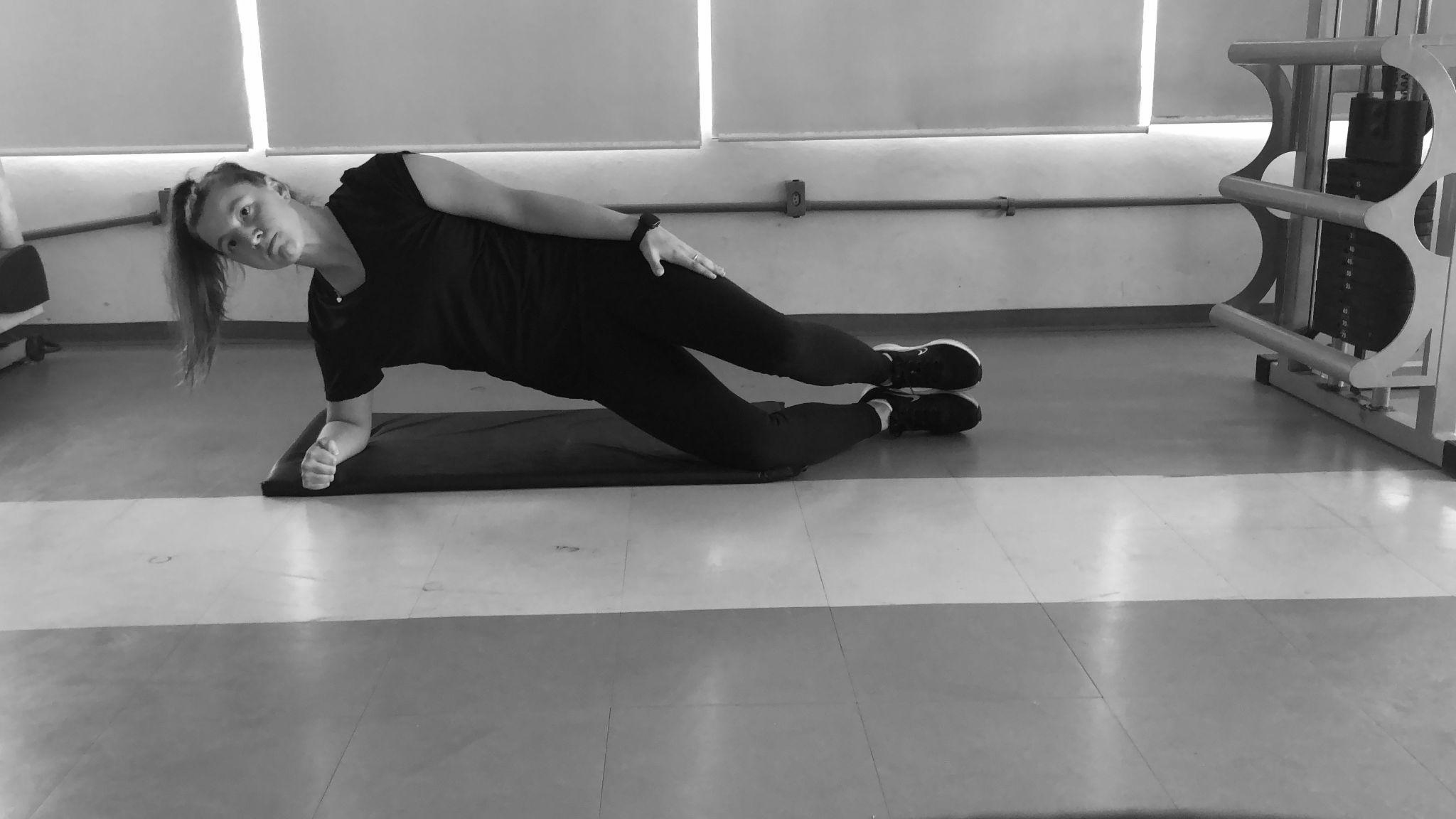** 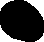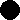 | | |
| **Superman (isometric)** | **Initial position:**  **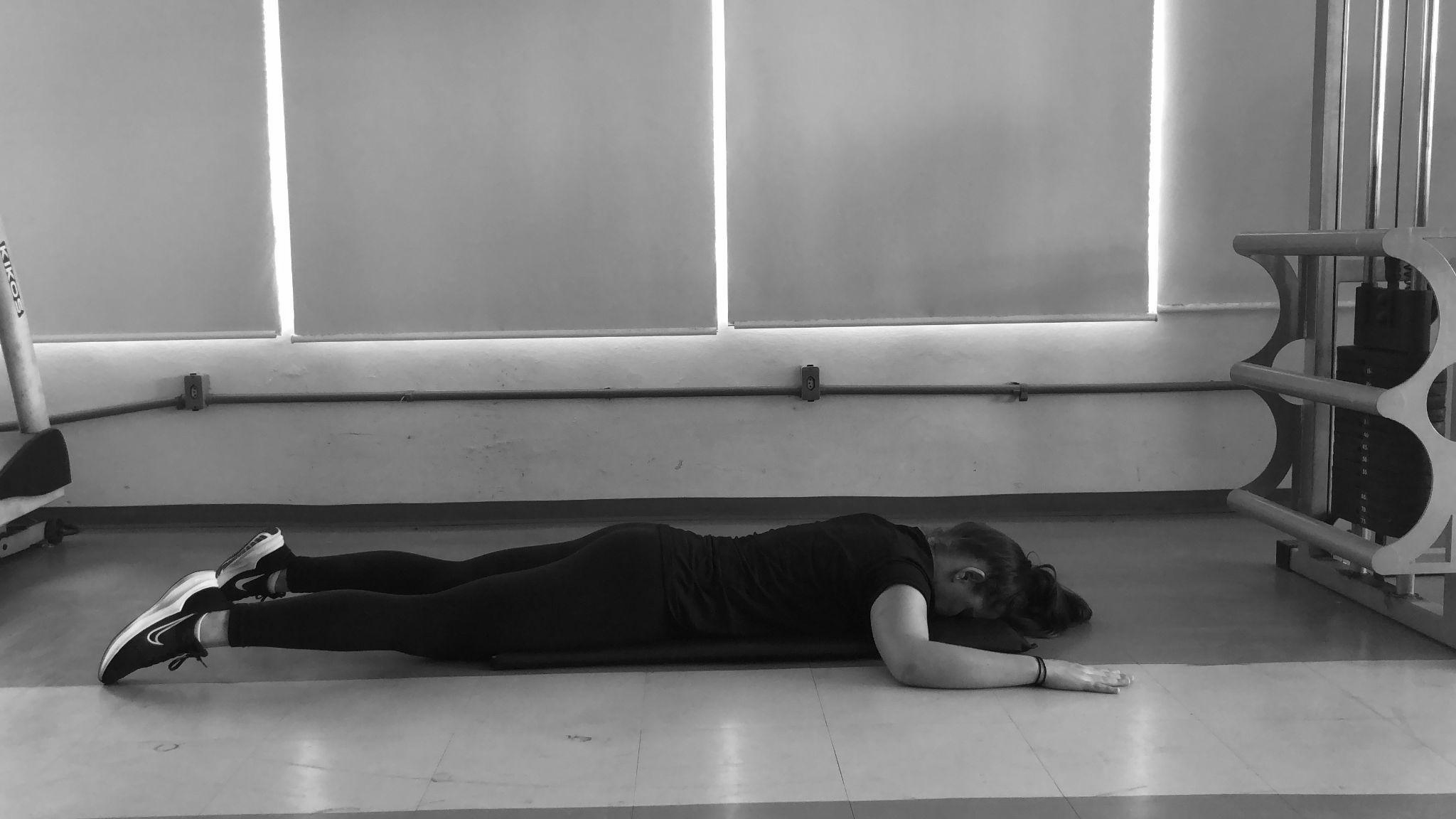** | **Final position:**  **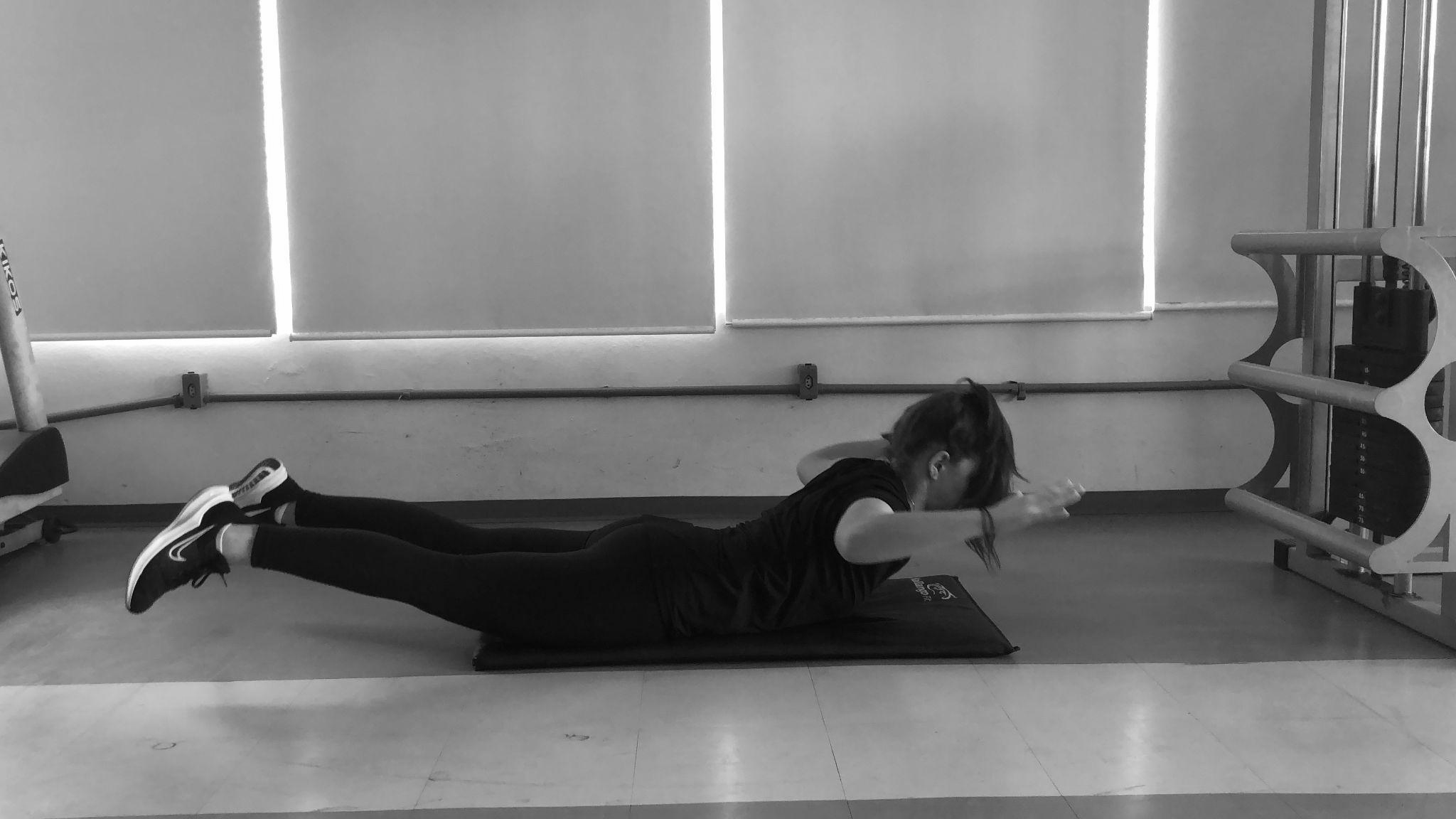** | | |
| ***Bird-dog*** | **Initial position:**  **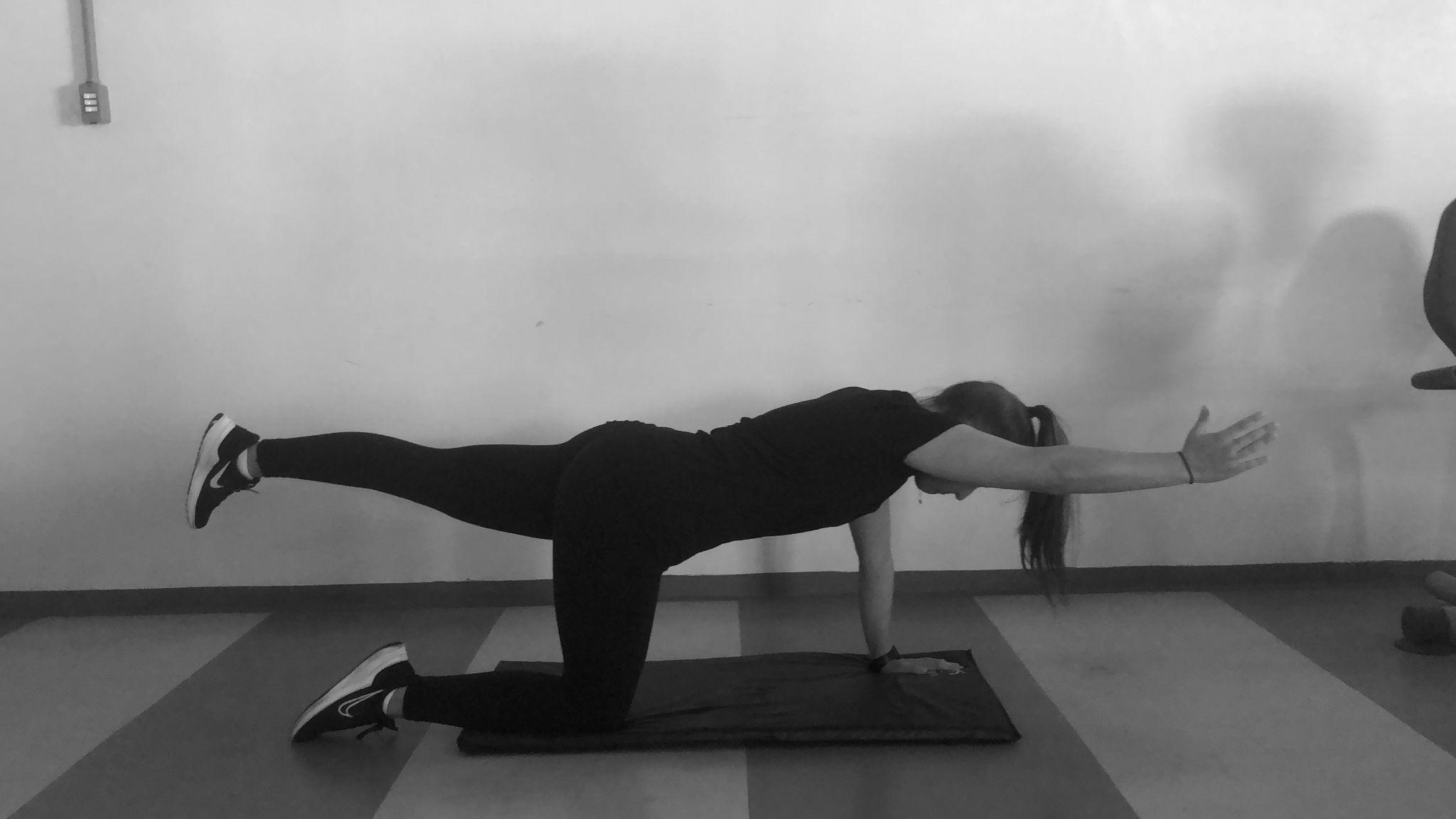** | **Final position:**  **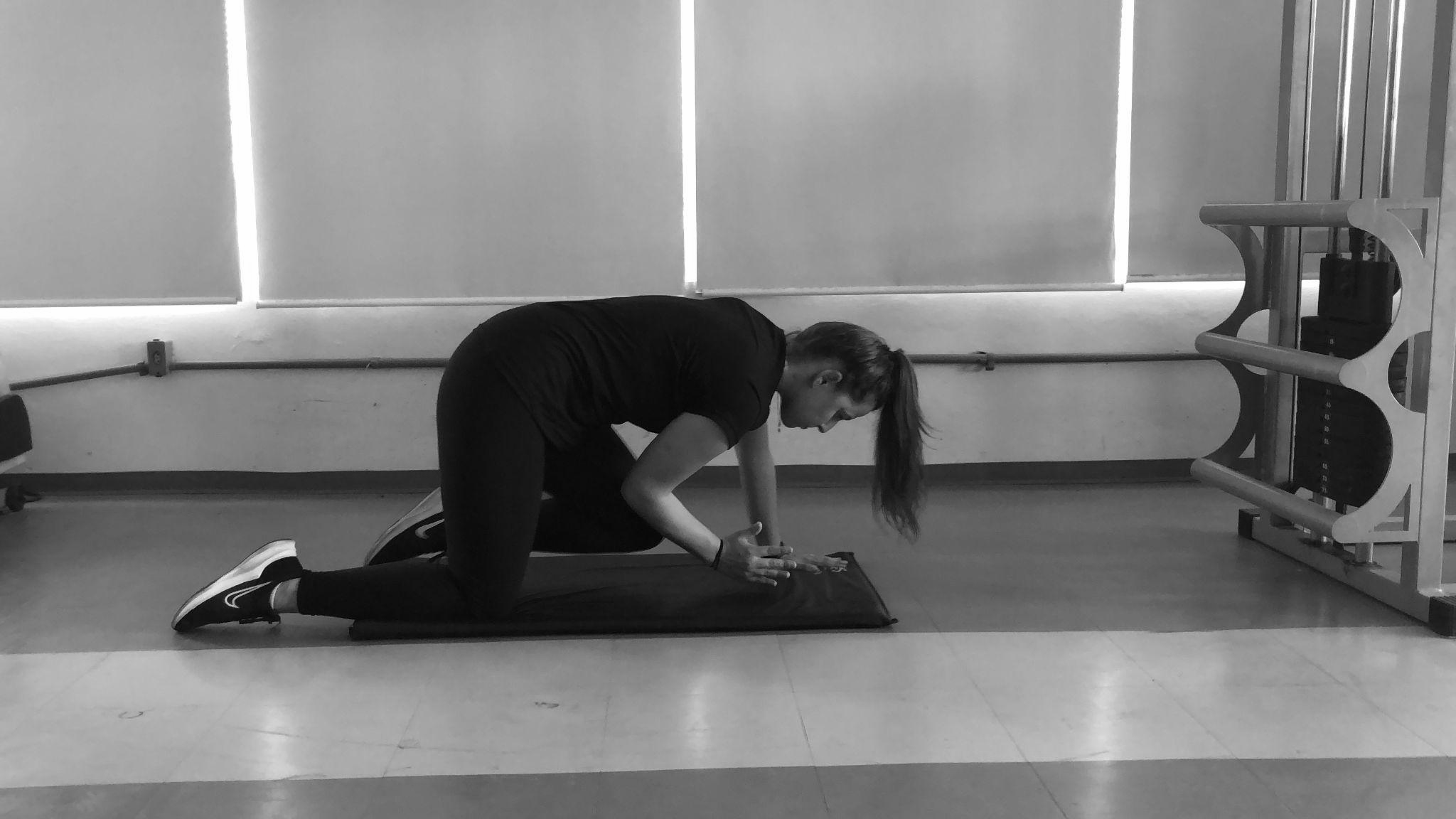** 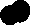 | | |
| **Isometric front plank with forearm support** | **Initial position:**  **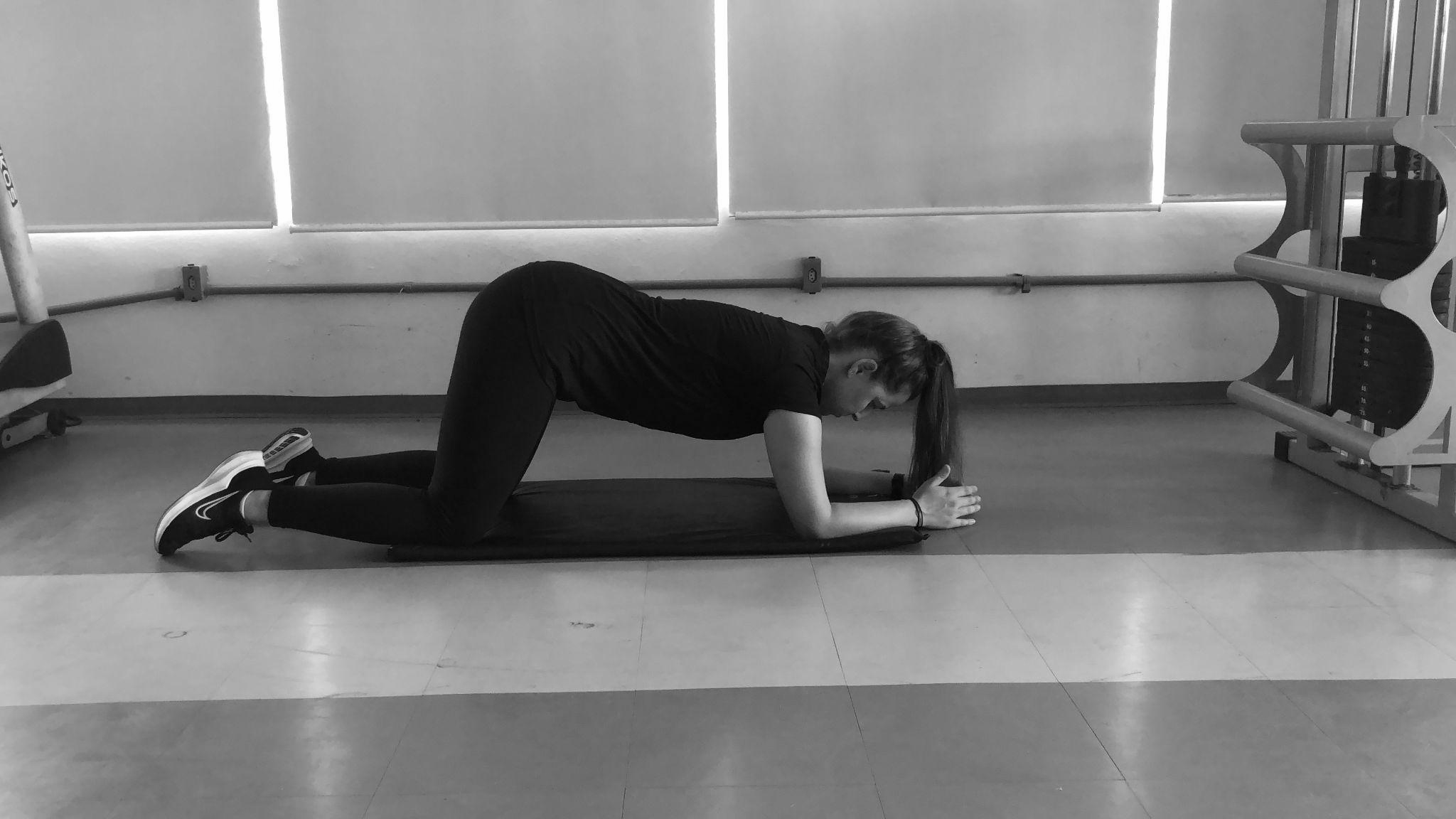** 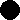 | **Final position:**  **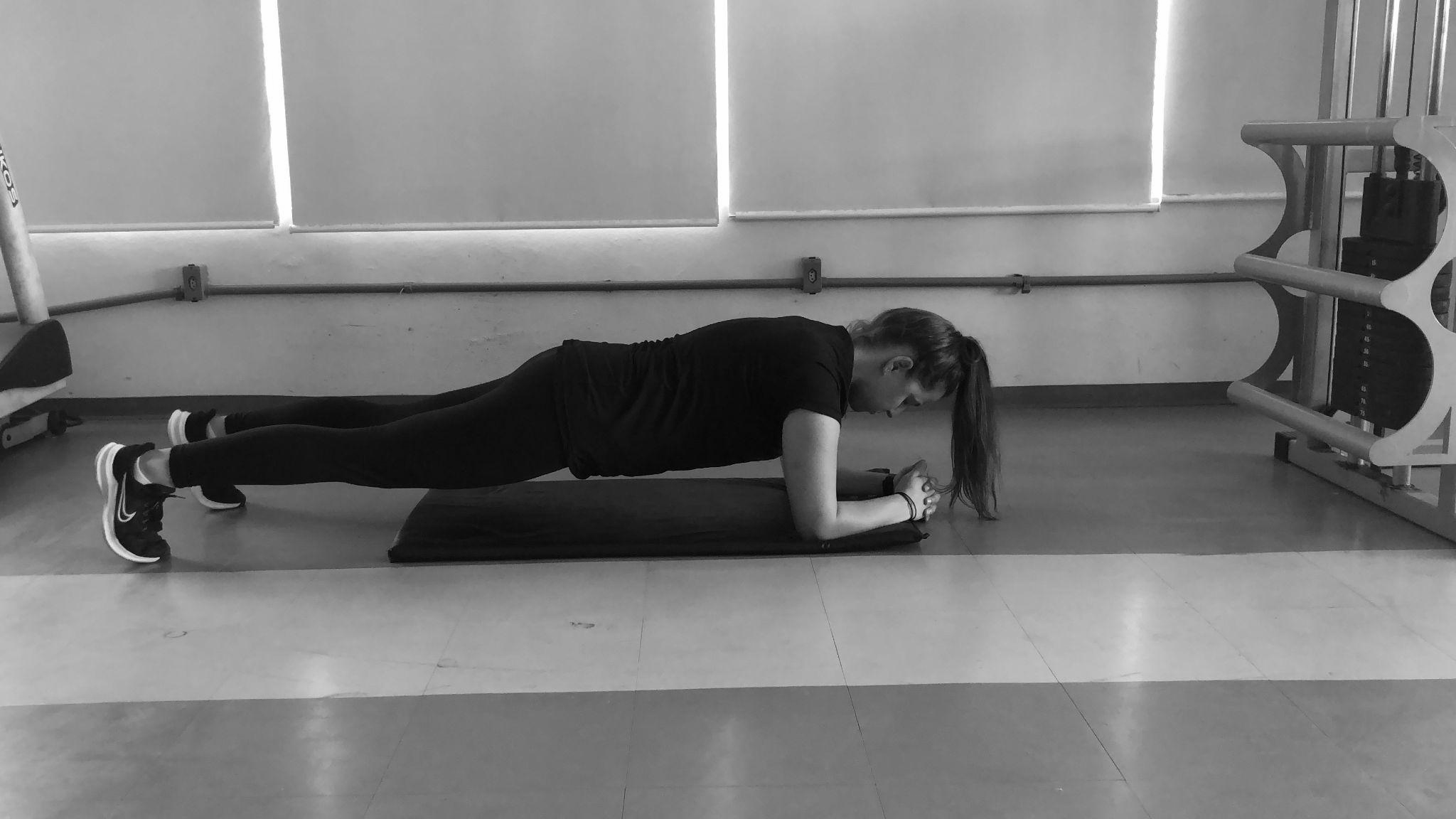** 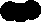 | | |
| **Main part (Intermediate stage)**  **Weeks 3-5** | | | | |
| **Single leg isometric front plank** | **Initial position:**  **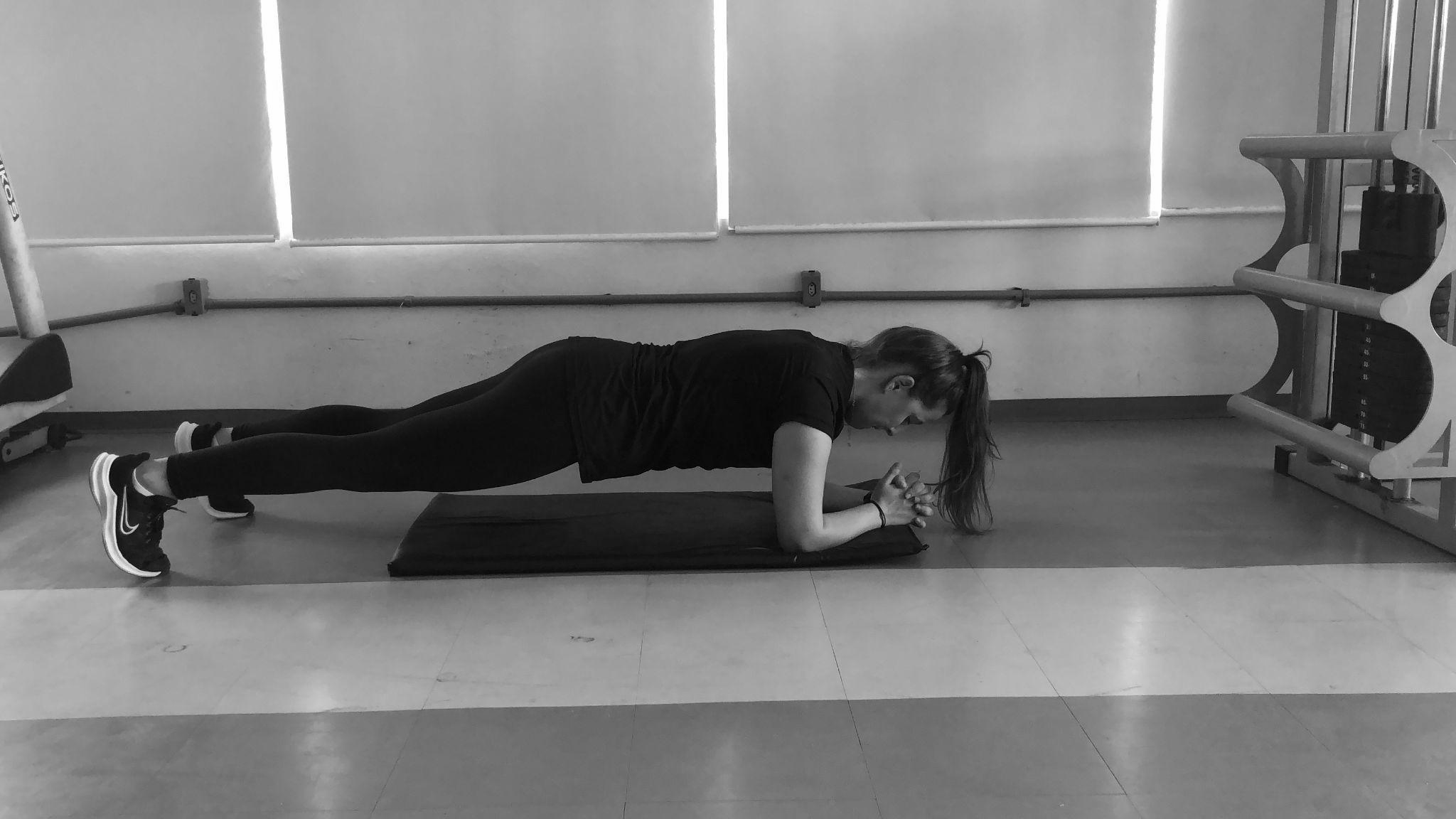** 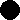 | **Final position:**  **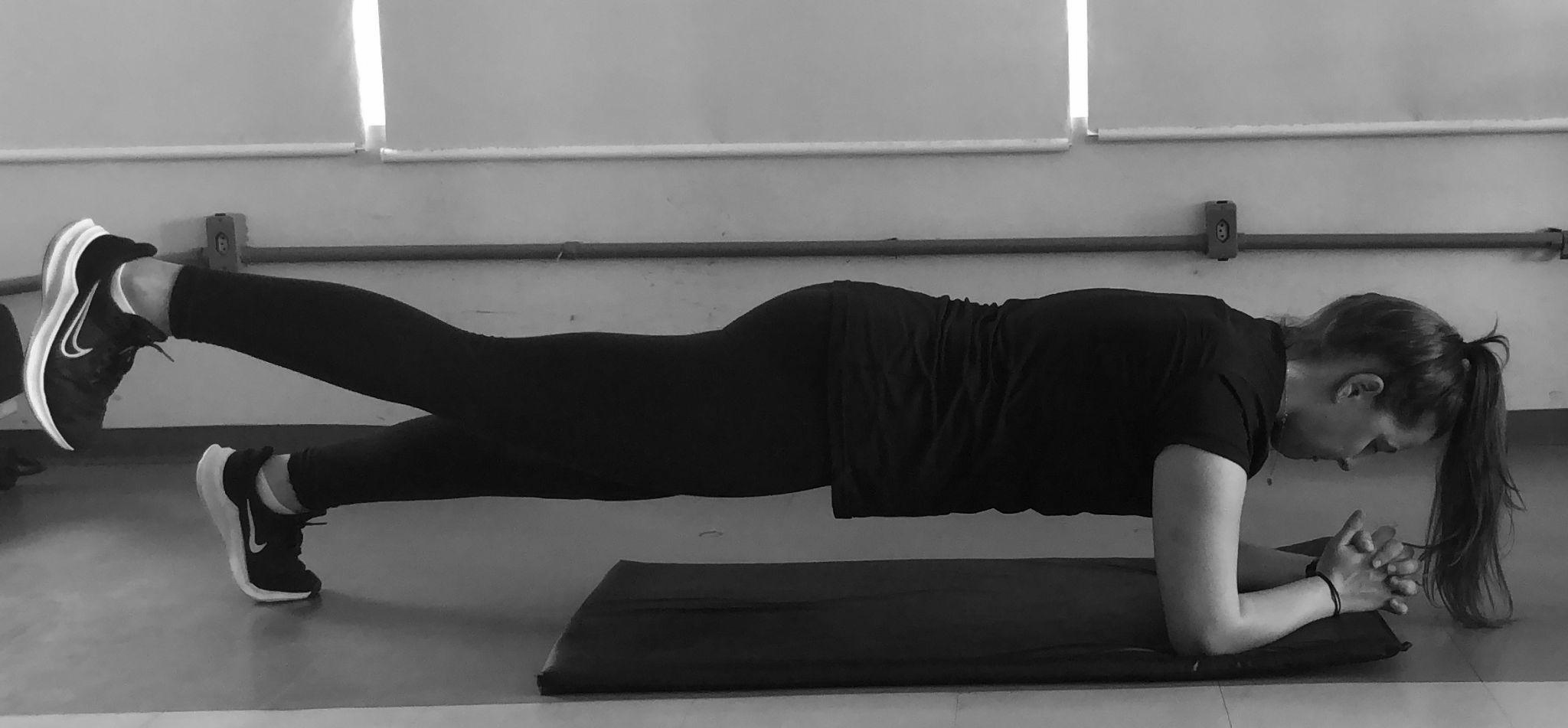** 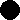 | | |
| **Single leg dynamic bridge** | **Initial position:**  **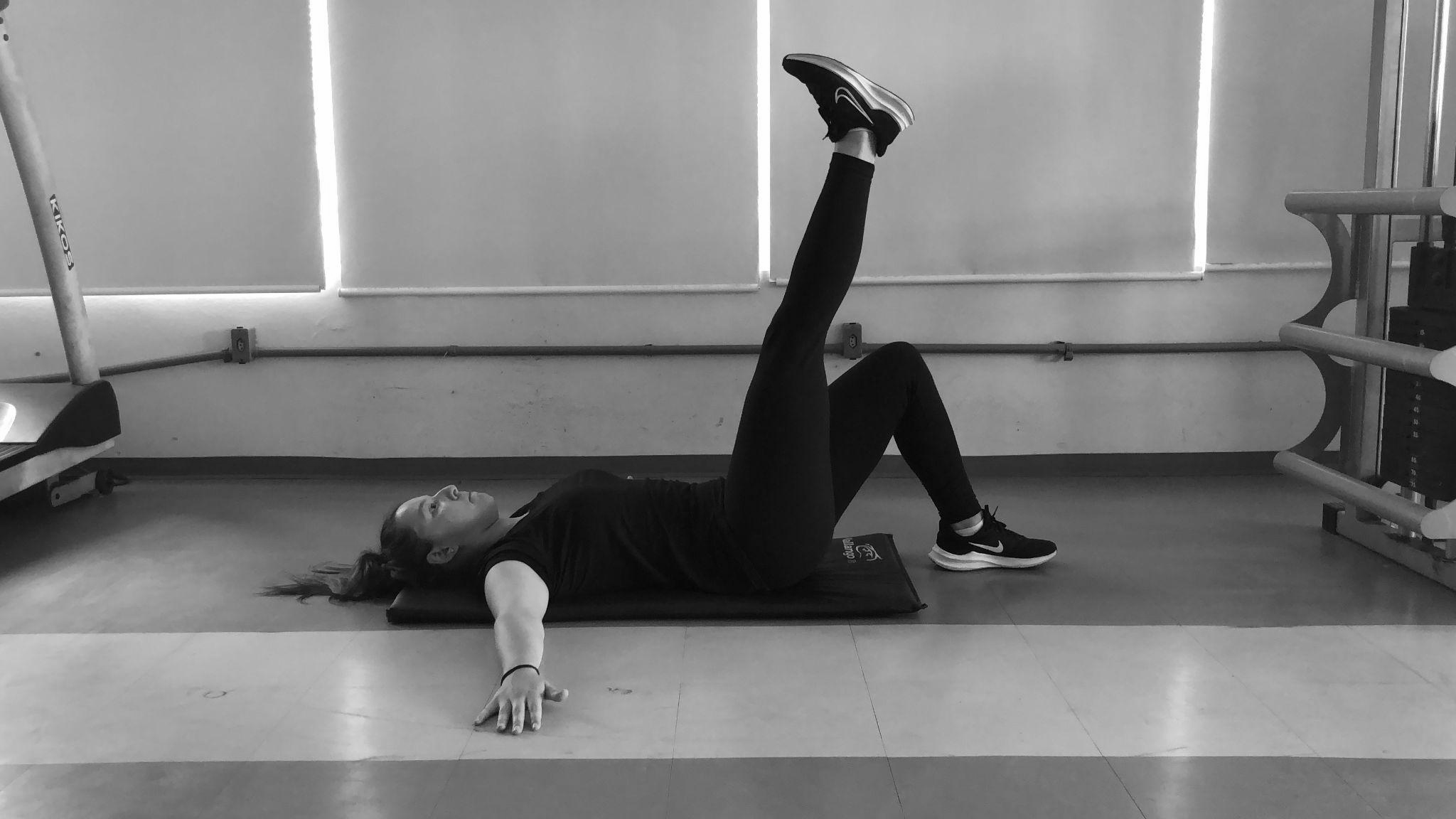** 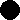 | **Final position:**  **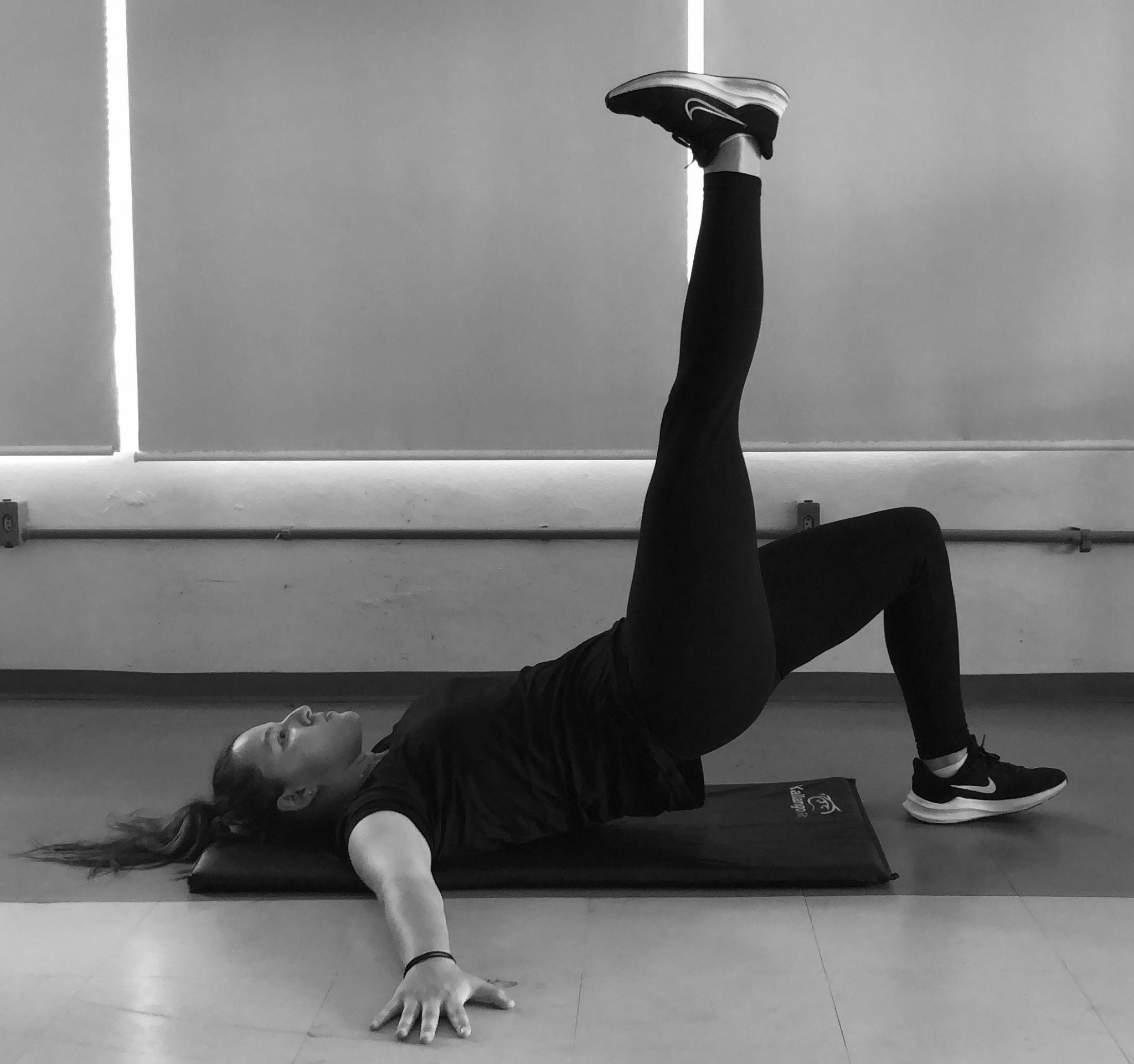** 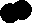 | | |
| **Isometric side plank with foot support** | **Initial position:**  **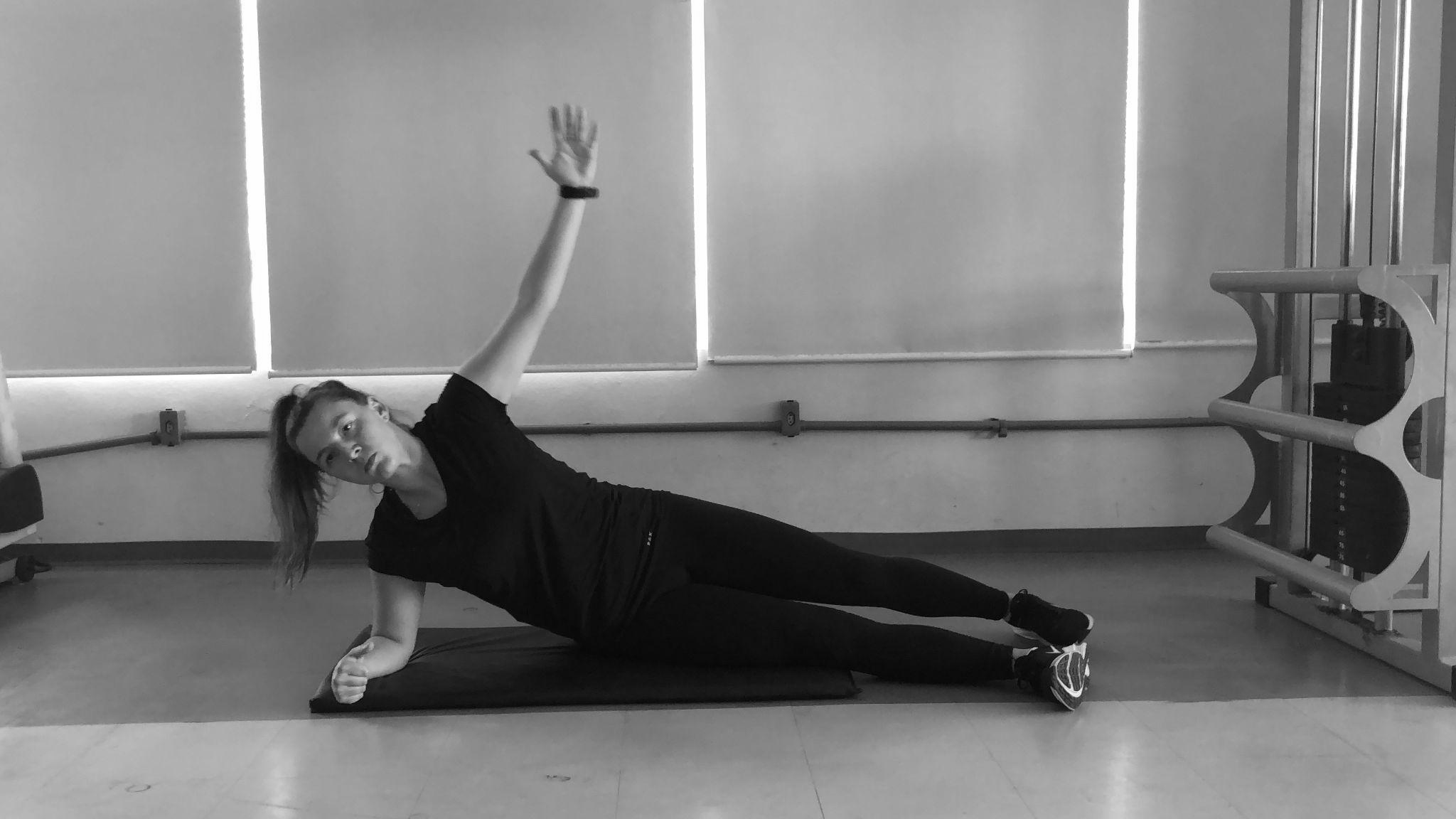** 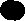 | **Final position:**  **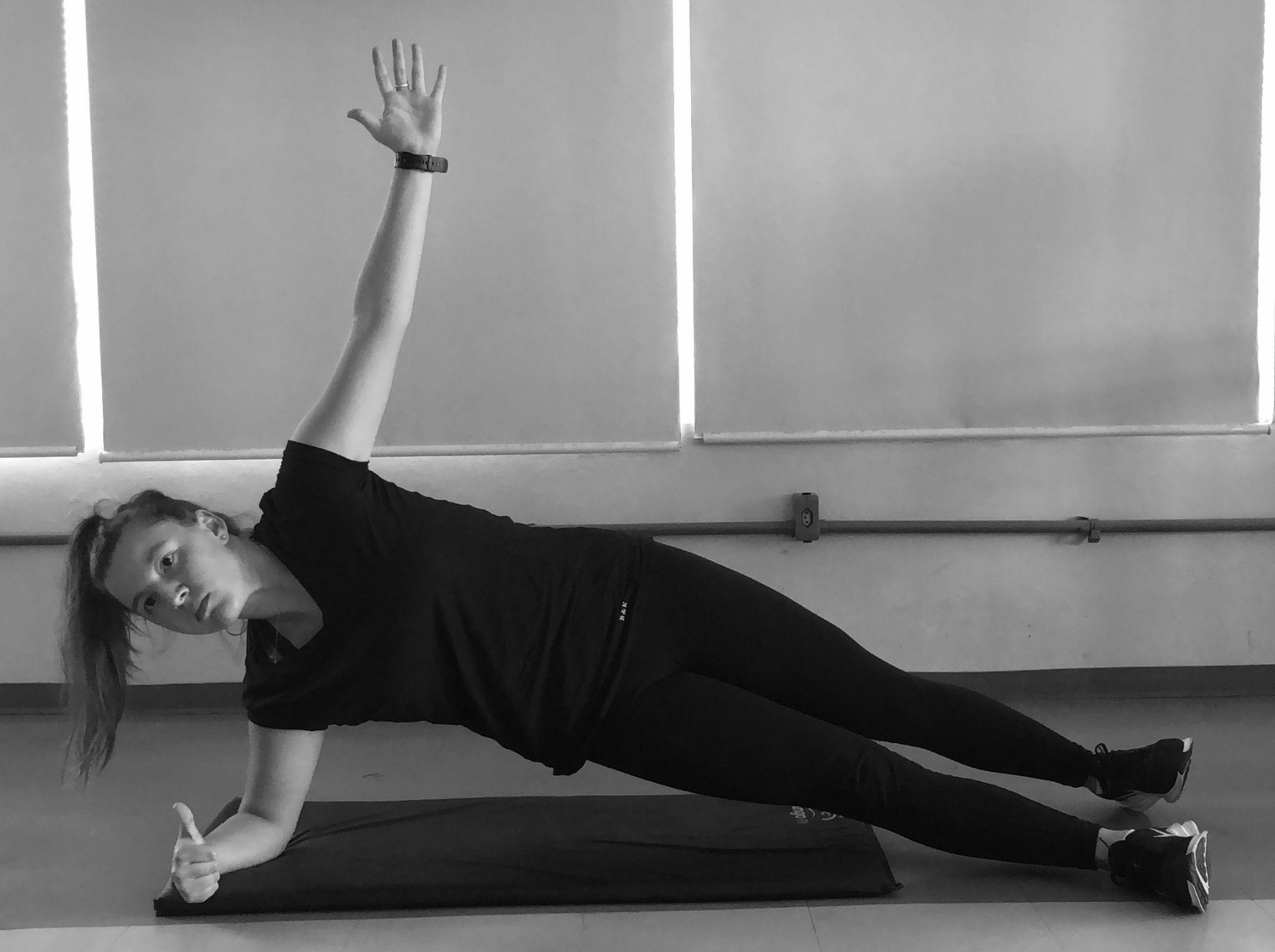** 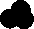 | | |
| **Superman with with upper limb movement** | **Initial position:**  **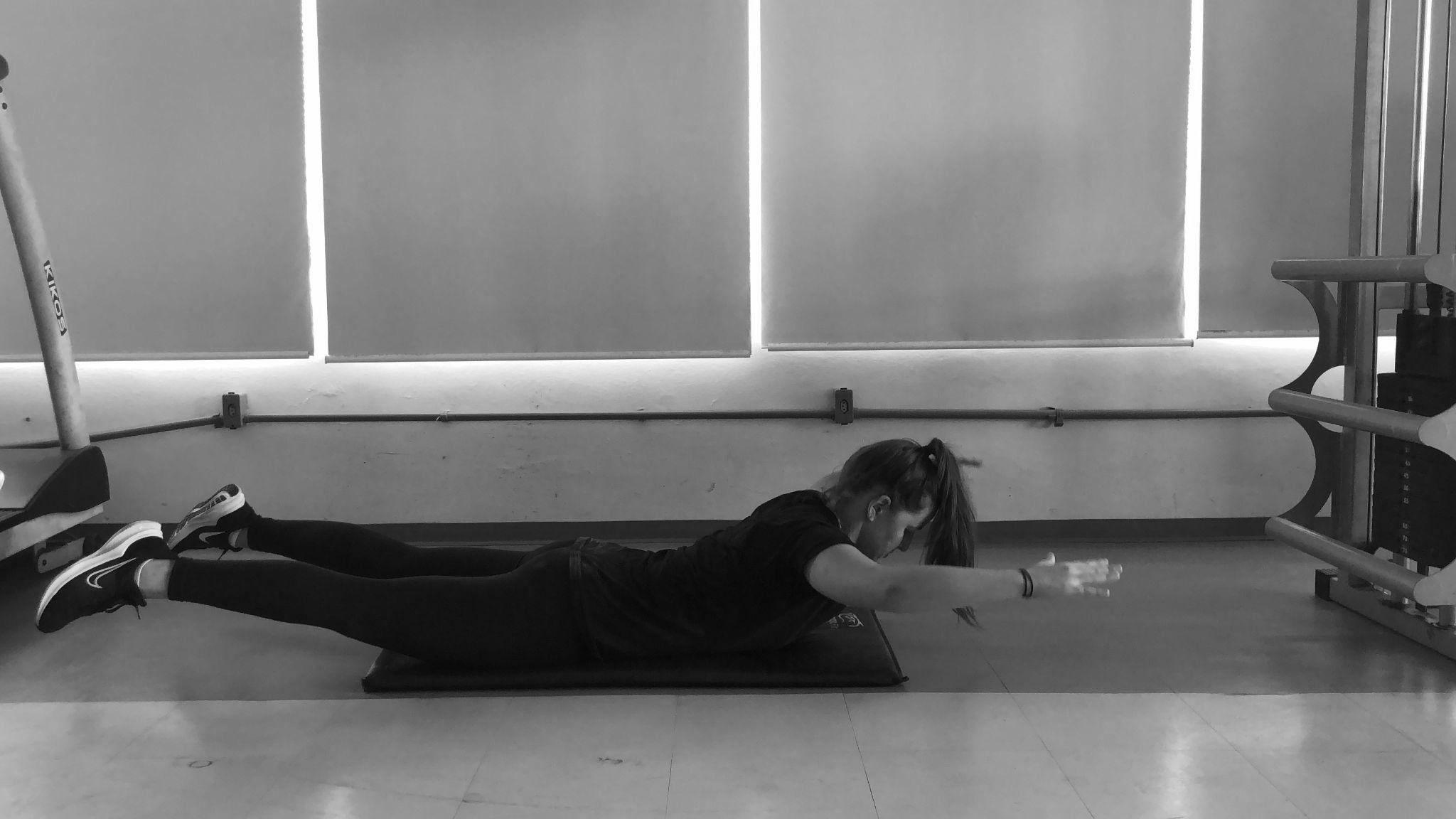** | **Trasition position:**  **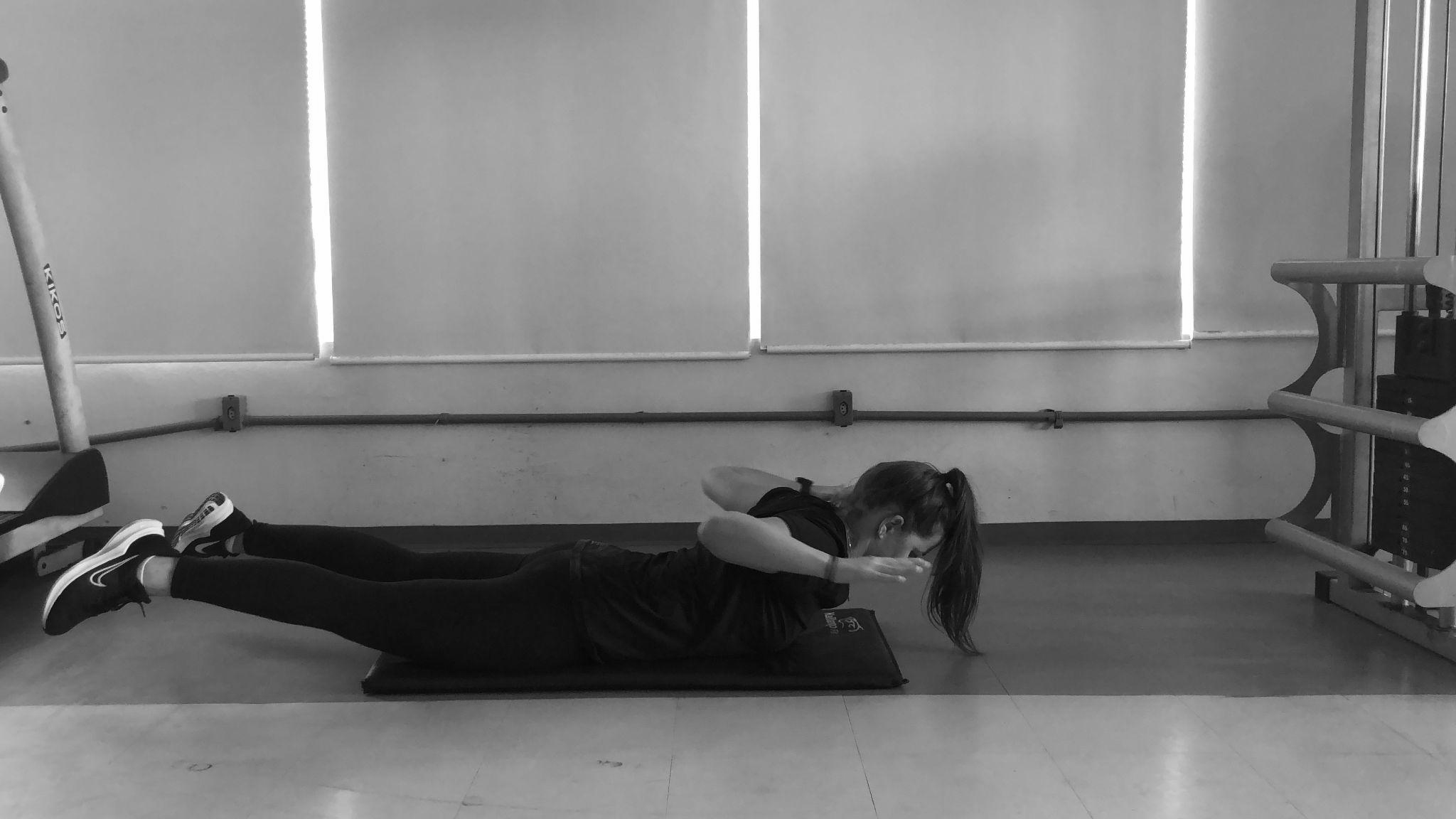** | **Final position:**  **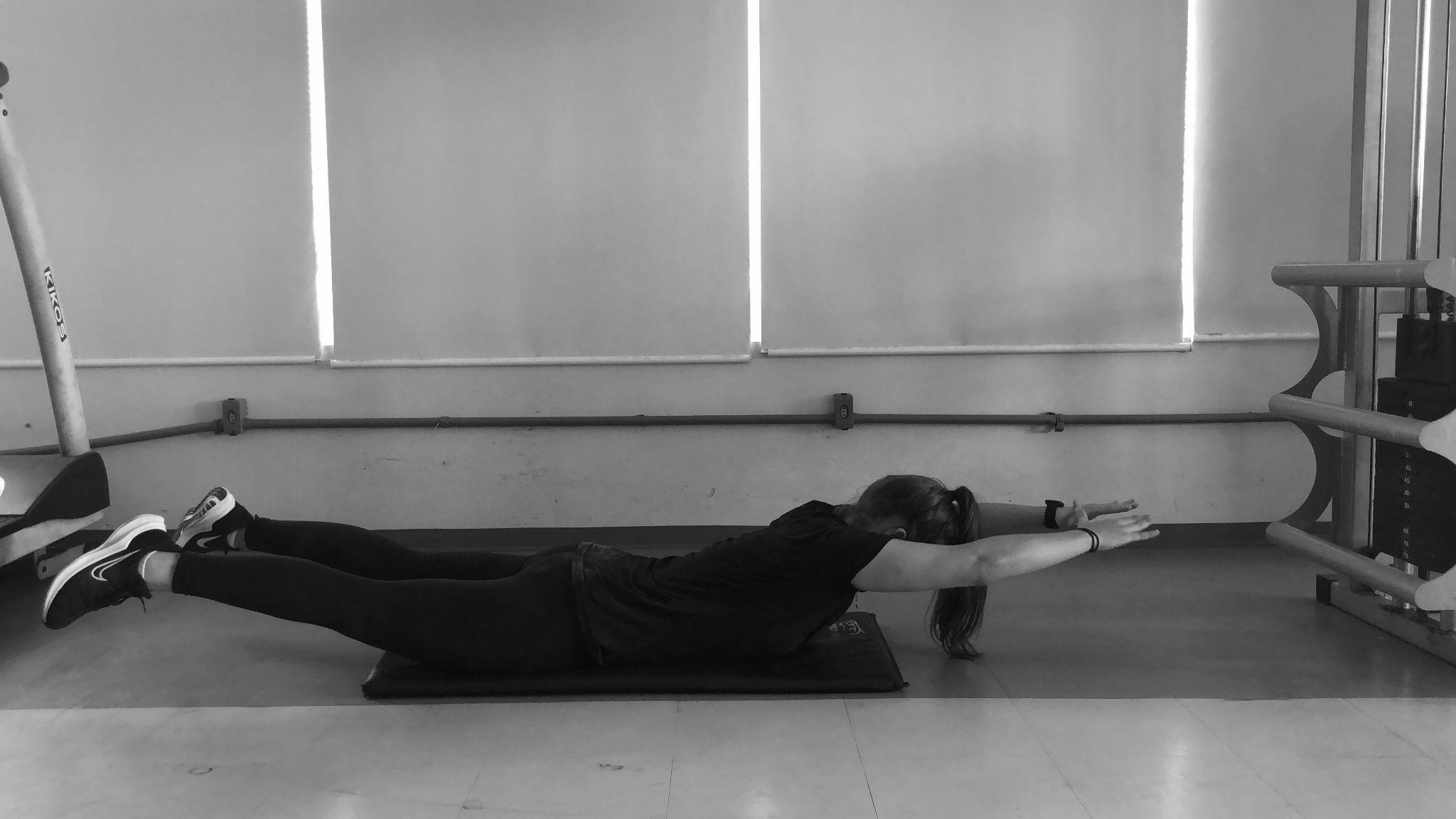** | |
| **Single leg dynamic bridge with heel support** | **Initial position:**  **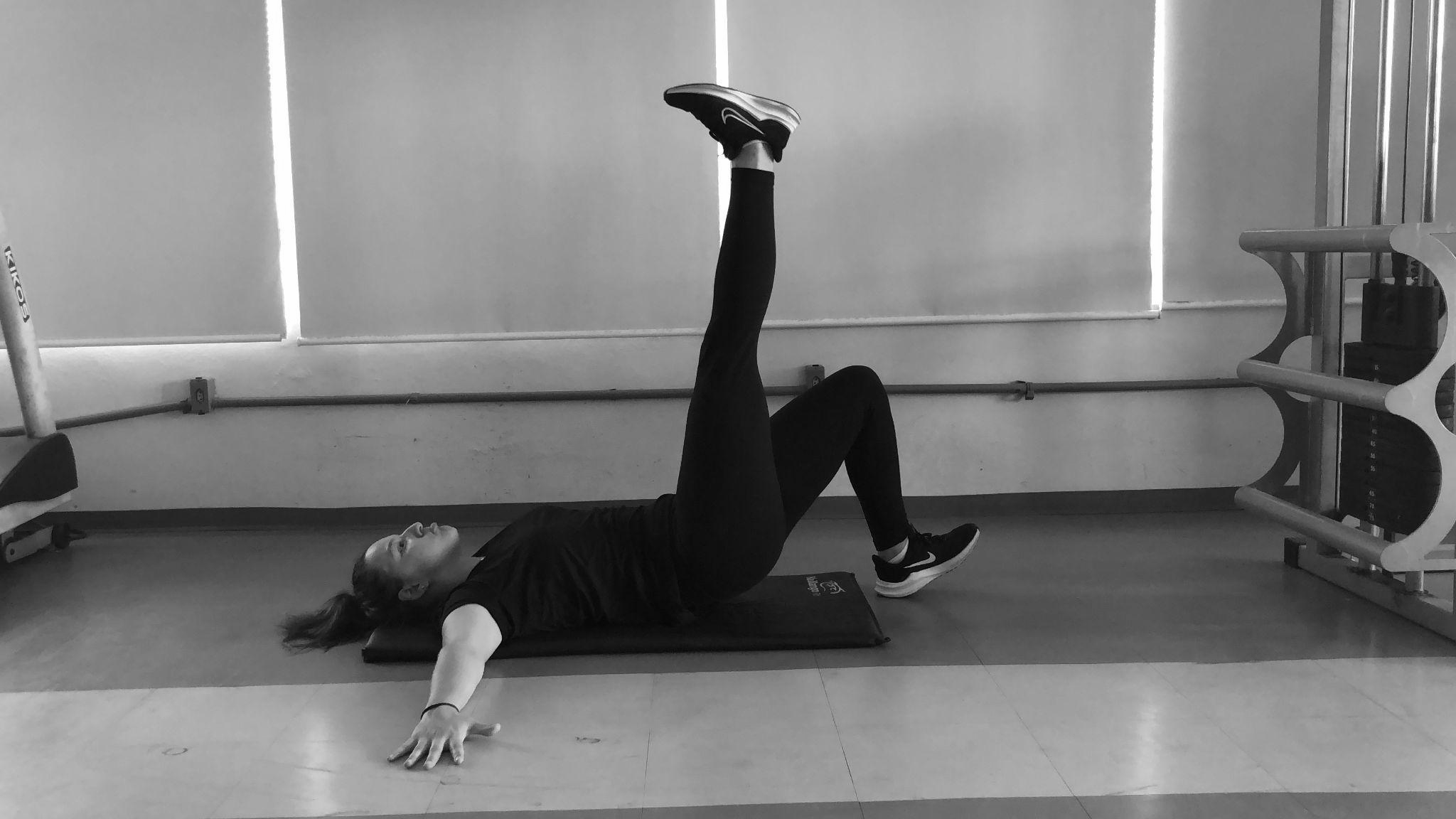** 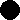 | **Final position:**  **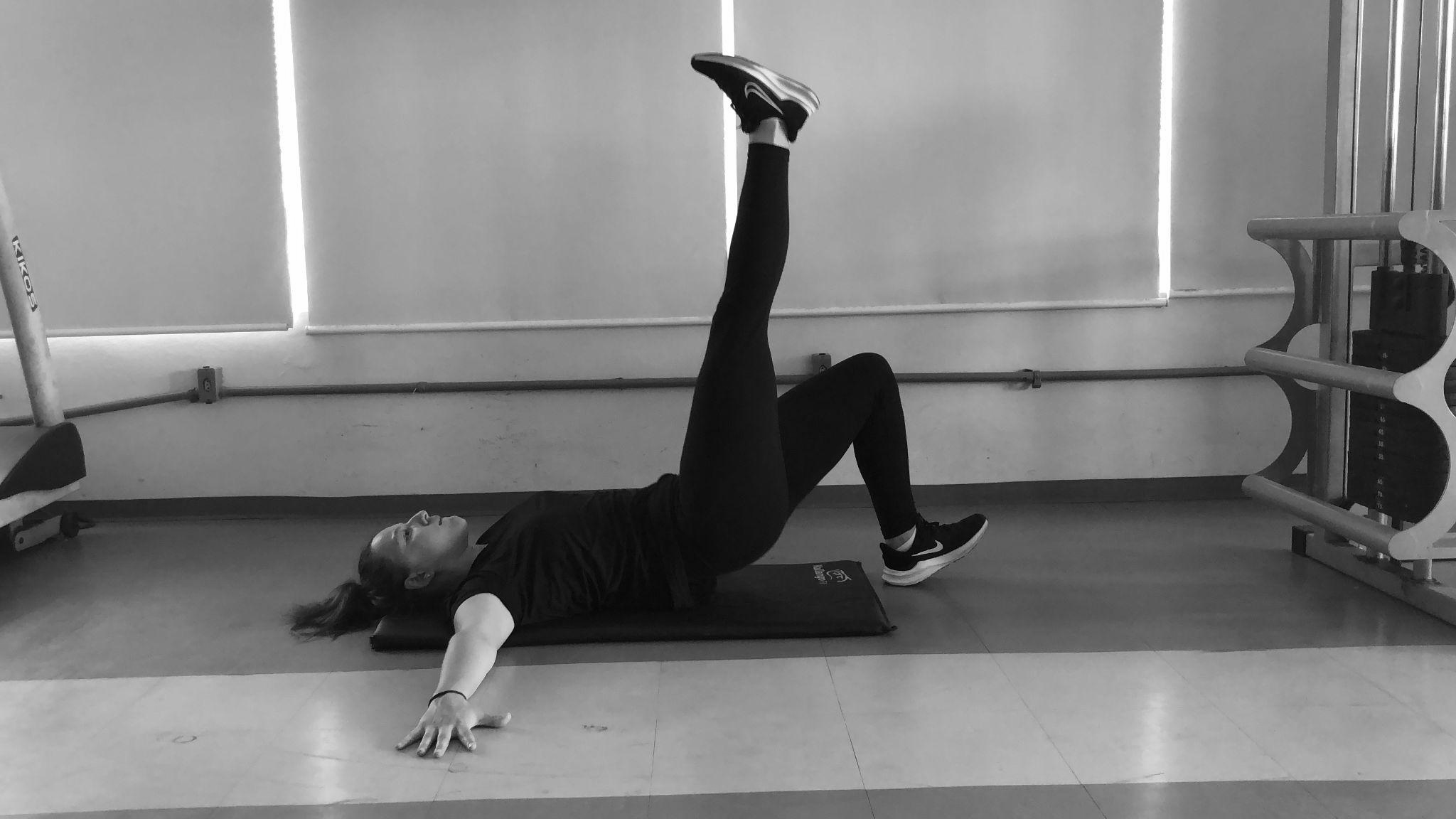** 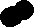 | | |
| **Main part (Advanced stage)**  **Weeks 6-8** | | | | |
| **Isometric front plank with diagonal support** | **Initial position:**  **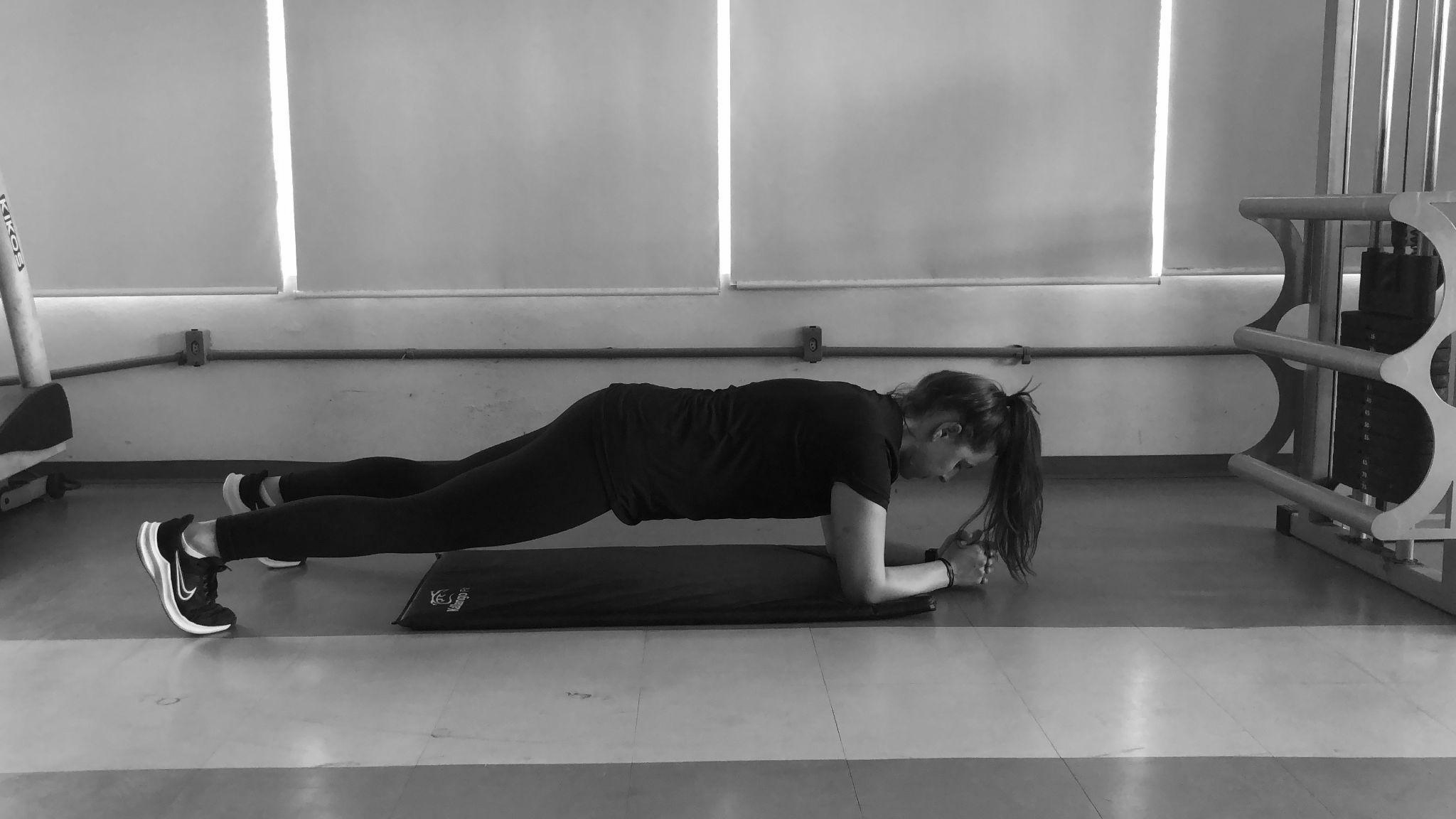** 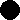 | **Final position:**  **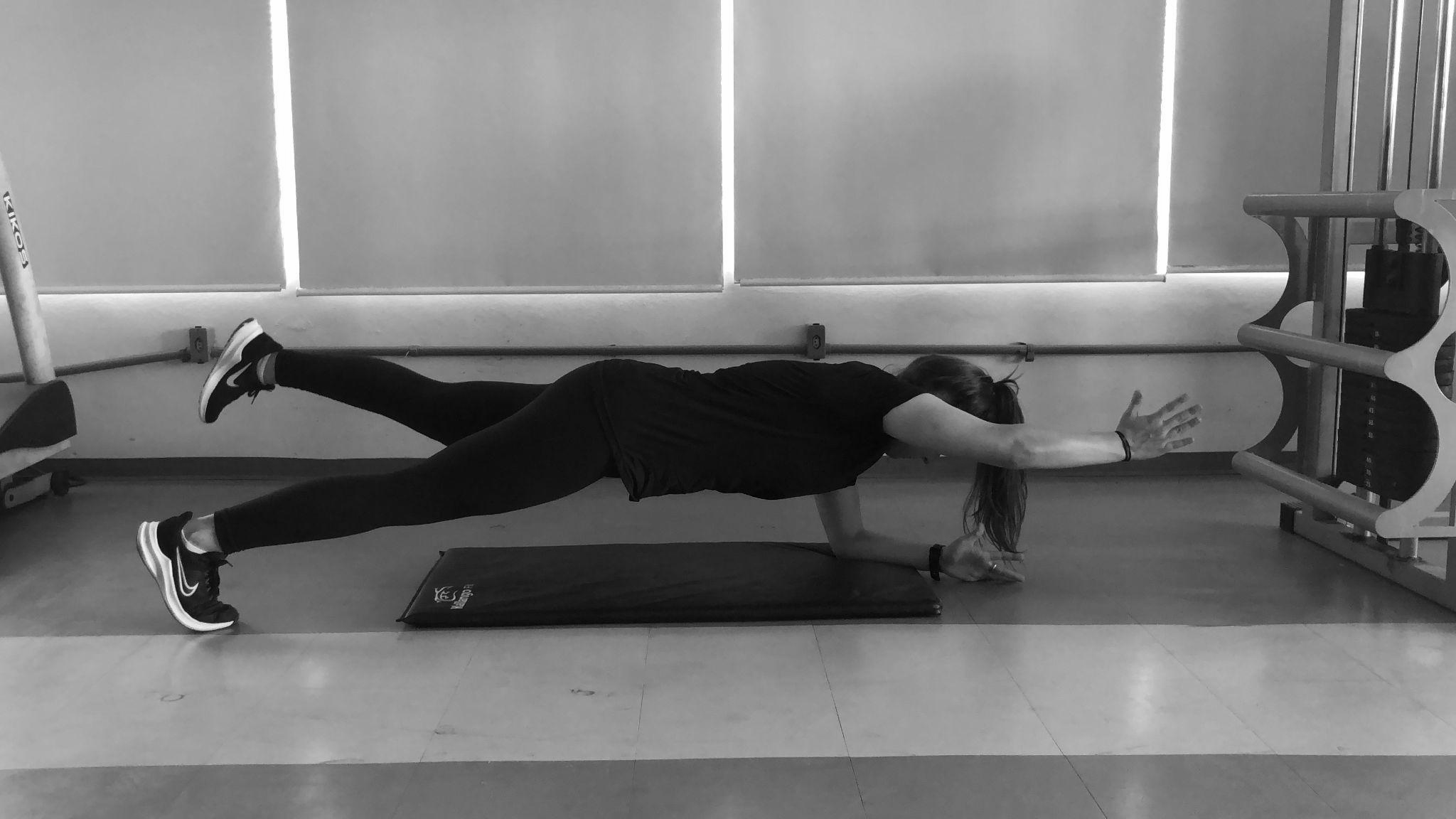** | | |
| **Side plank with one foot support** | **Initial position:**  **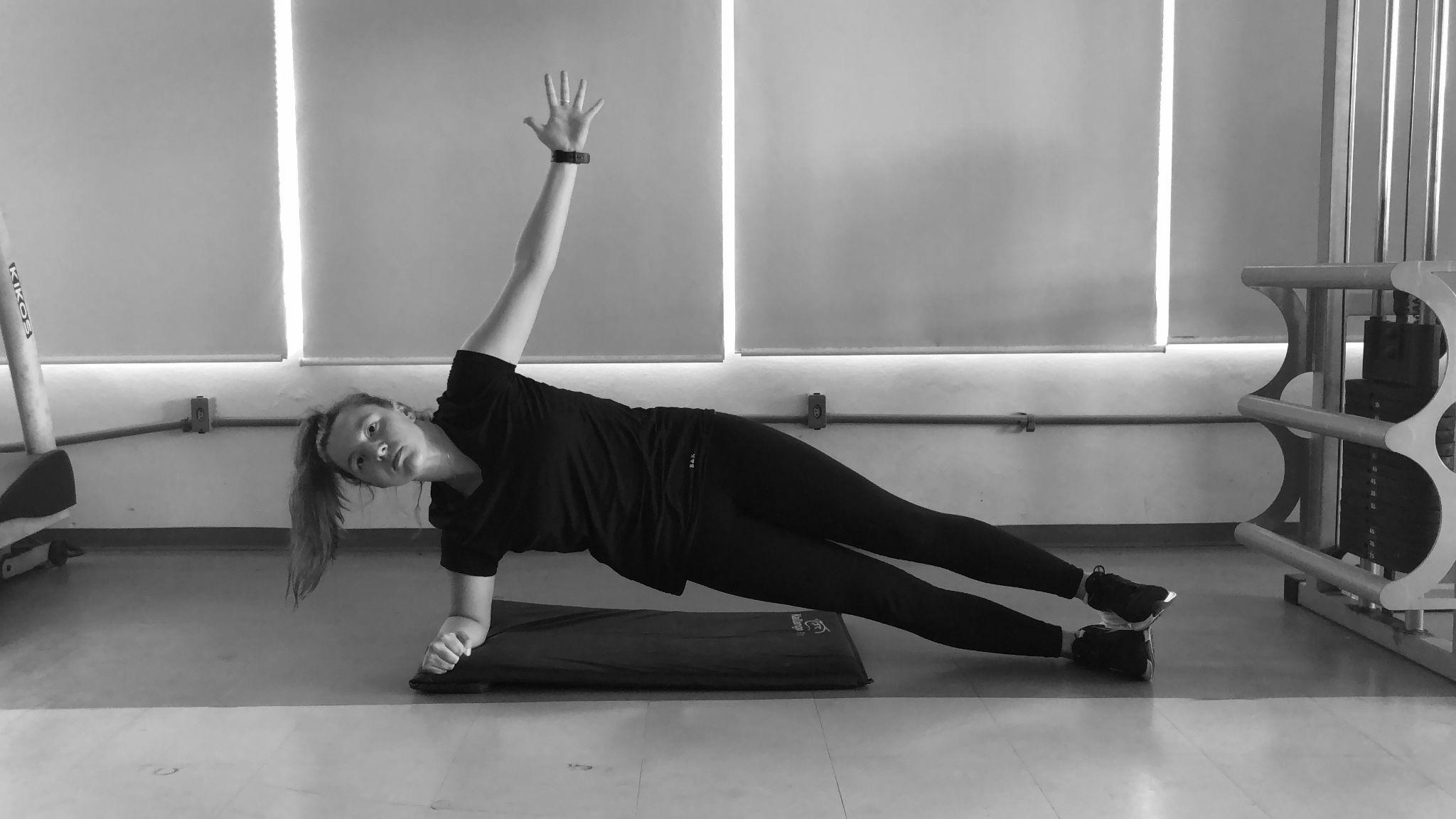** 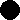 | **Final position:**  **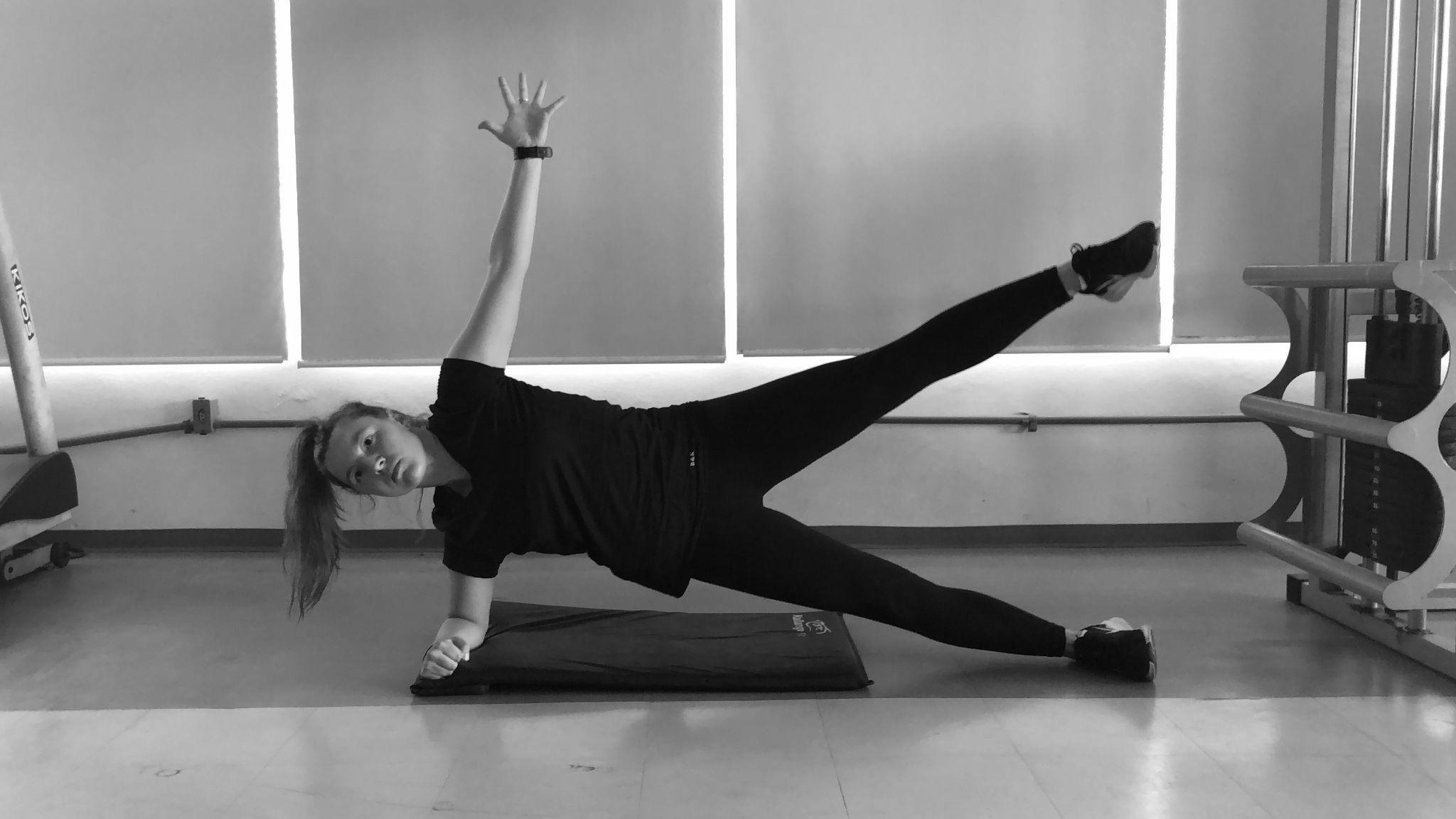** 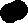 | | |
| **Superman (dynamic)** | **Initial position:**  **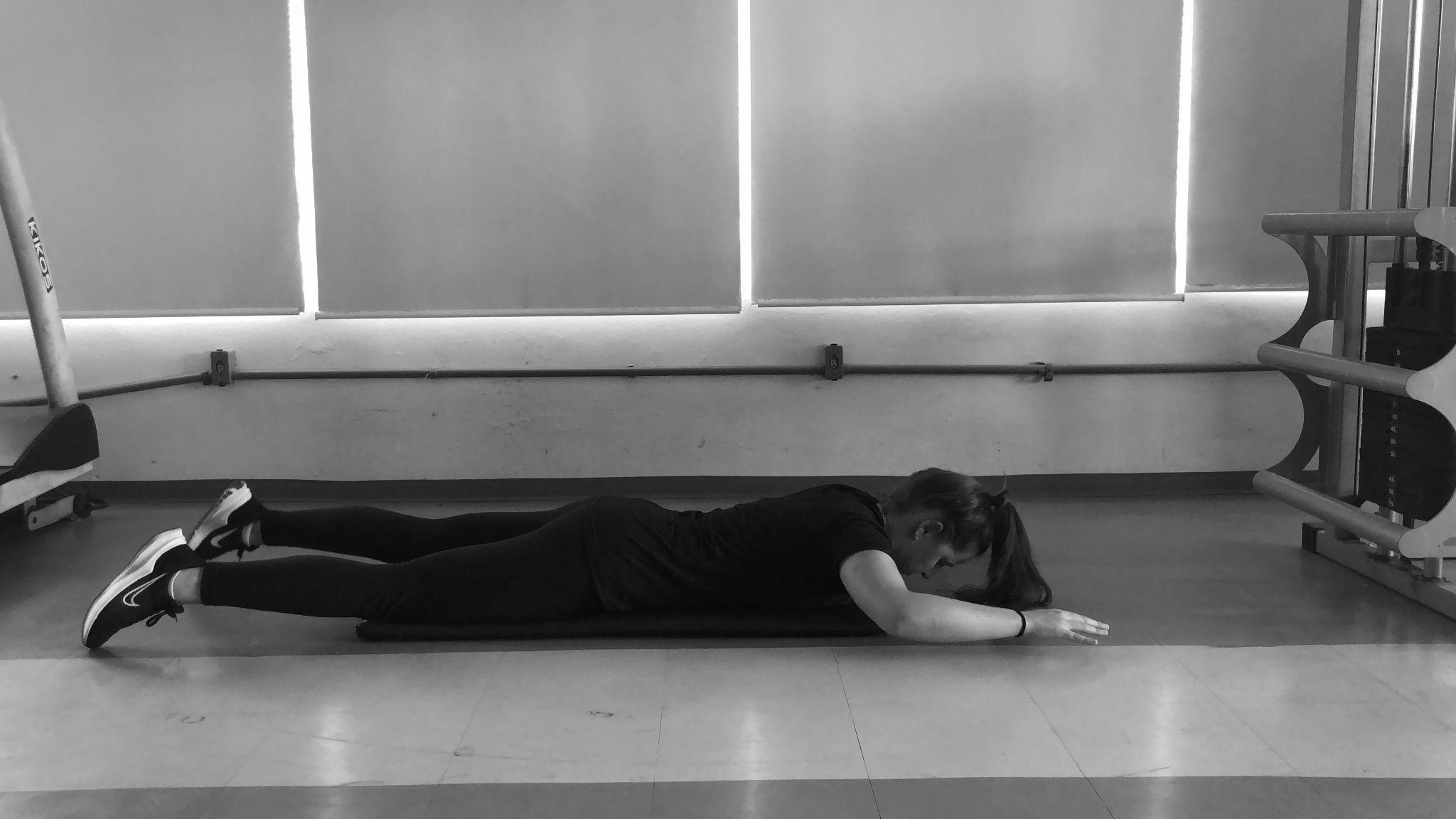** | **Final position:**  **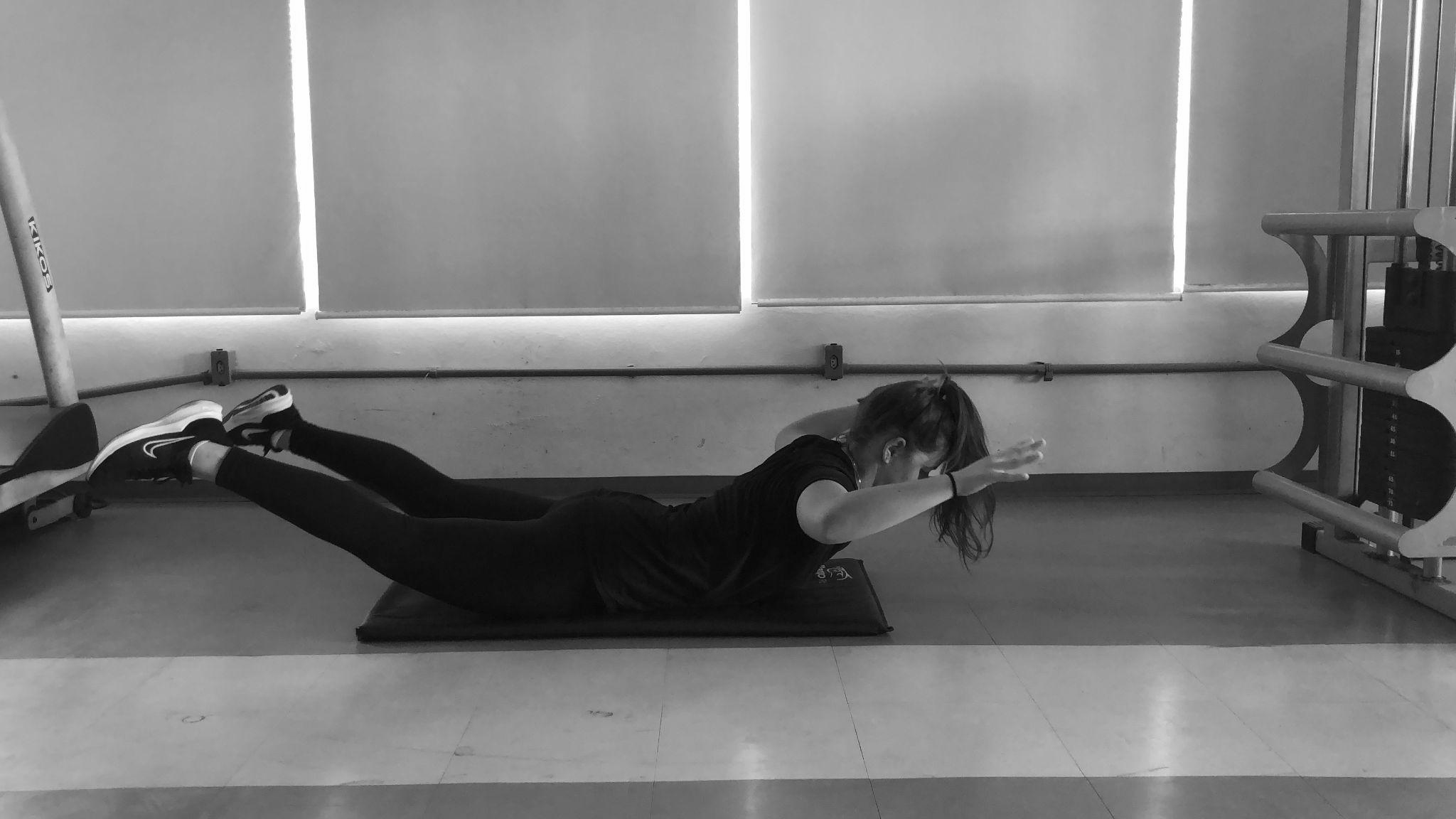** | | |
| **Bird-dog with elastic band resistance** | **Initial position:**  **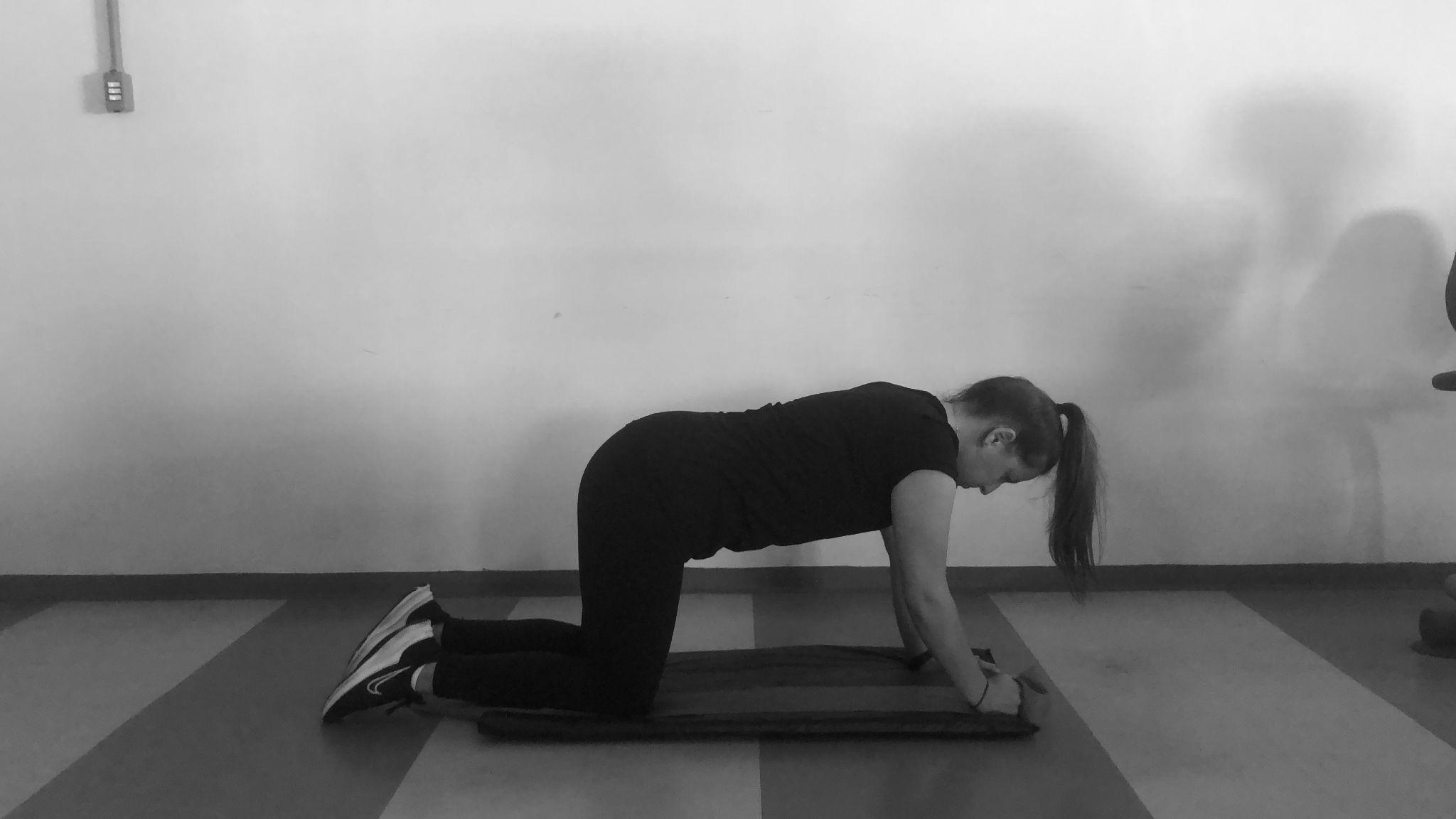** 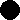 | **Final position:**  **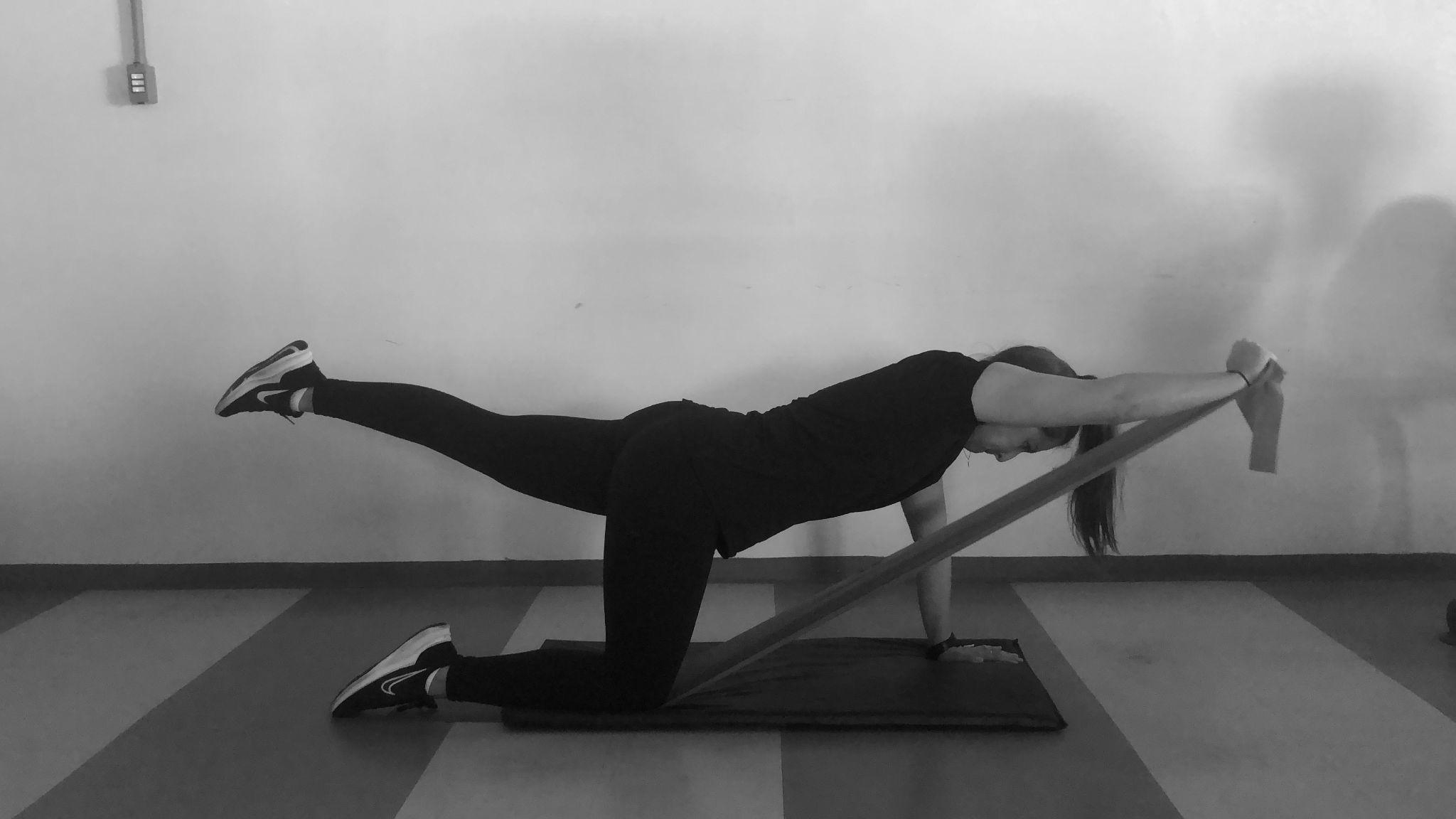** 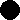 | | |
| **Dynamic trunk rotation with elastic band** | **Initial position:**  **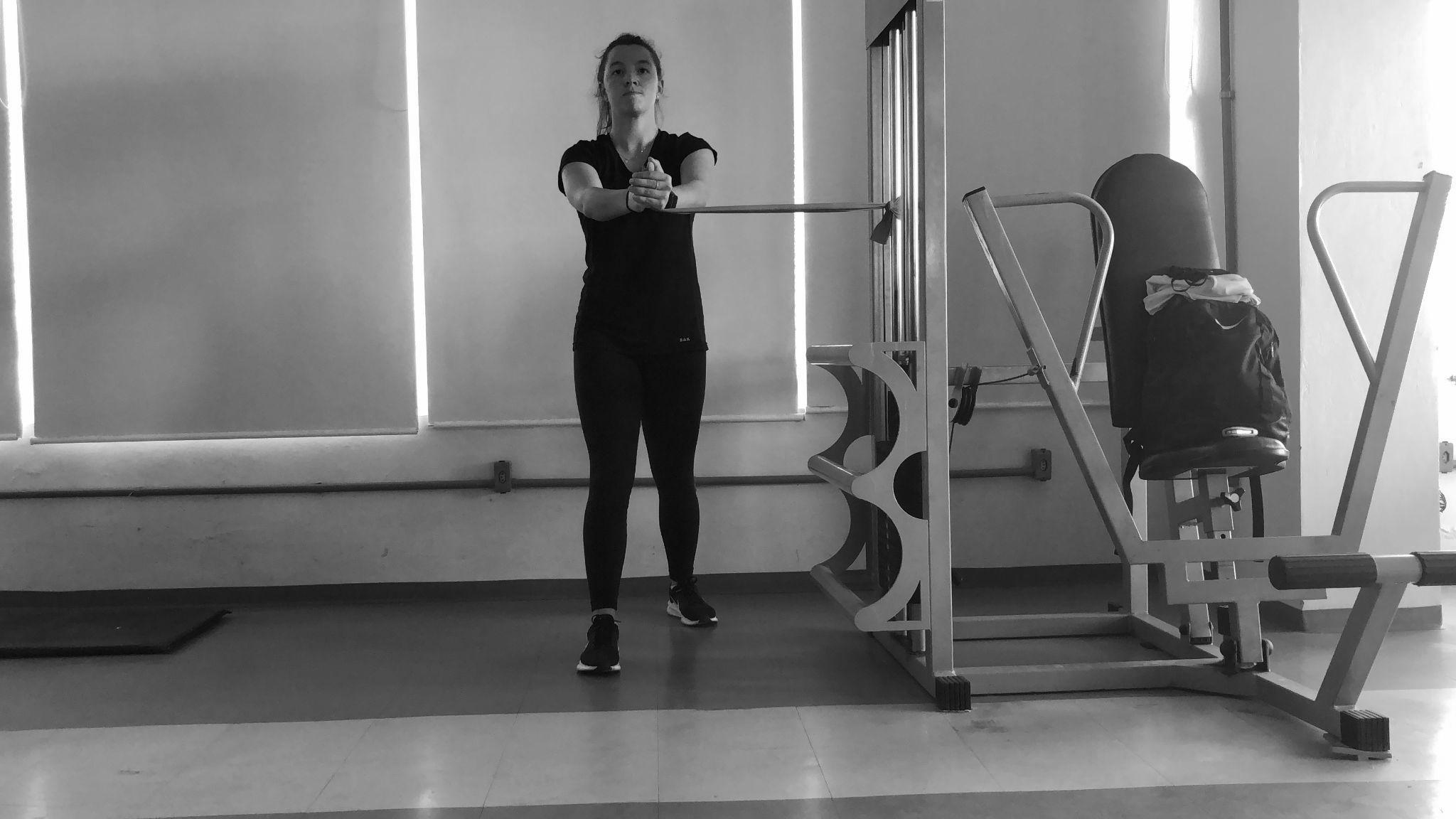** 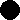 | **Final position:**  **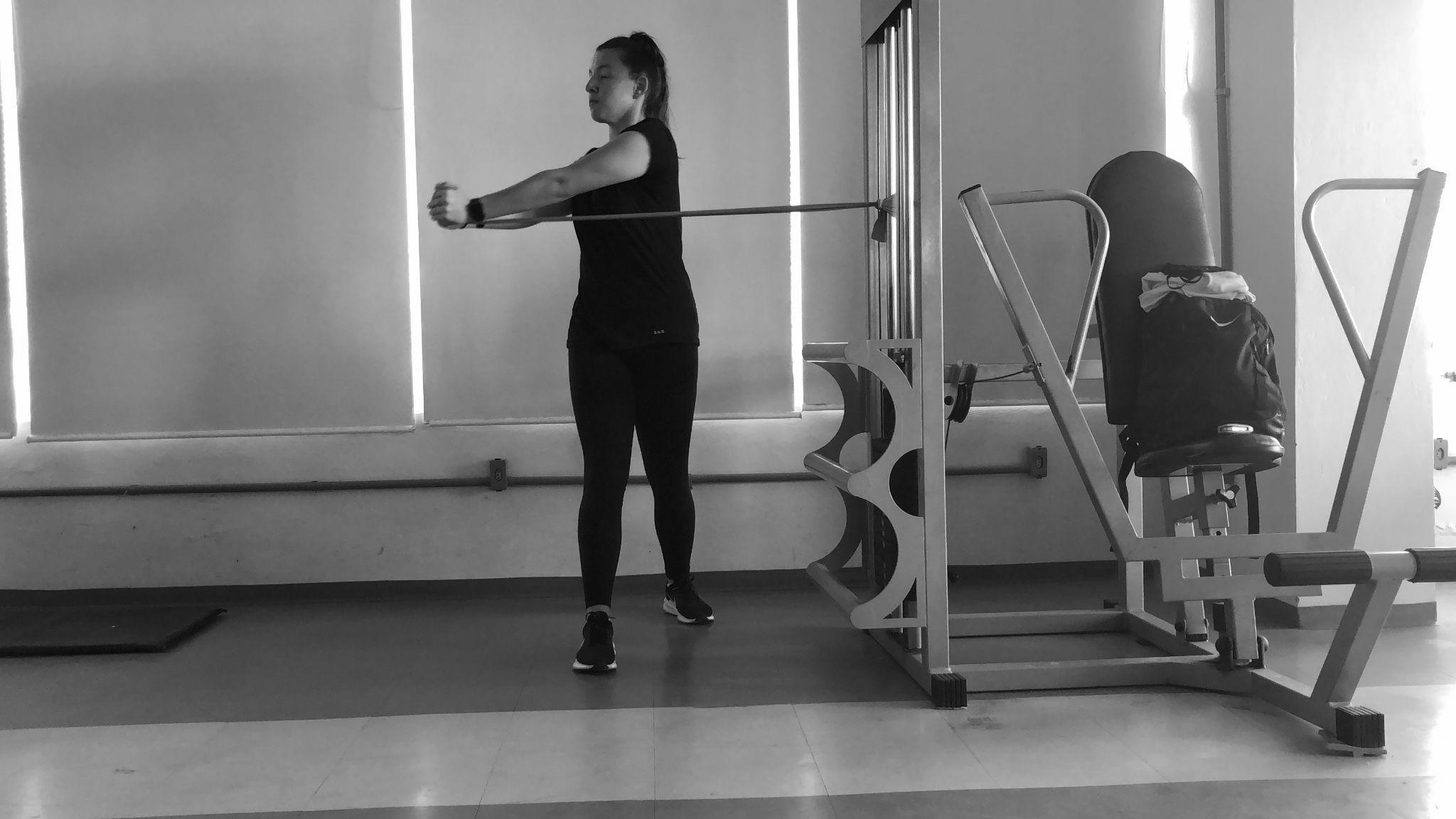** 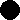 | | |
| **Calm down (stretching)** | | | | |
| **Sit on the heel** | **Initial position:**  **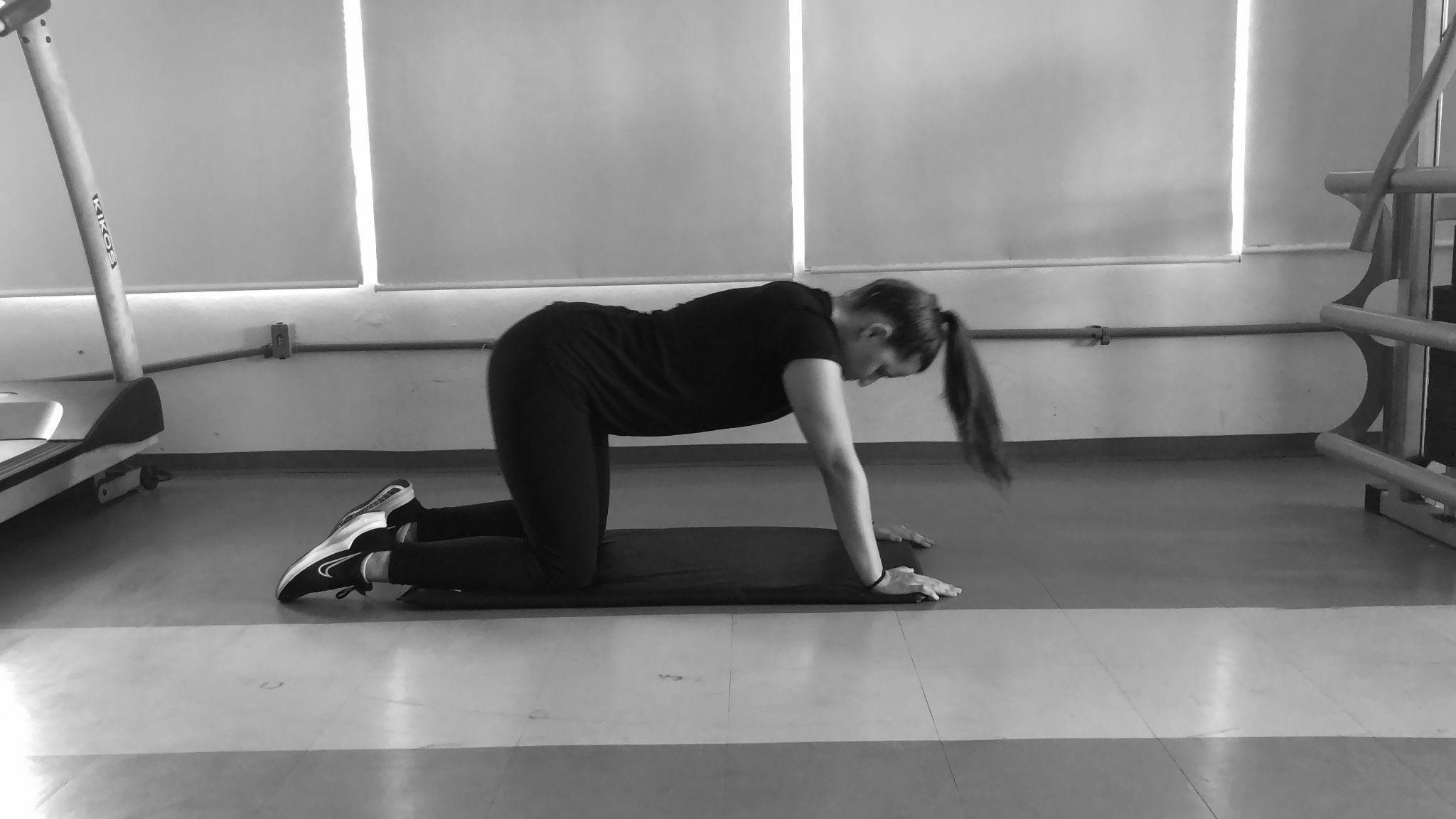** 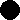 | **Final position:**  **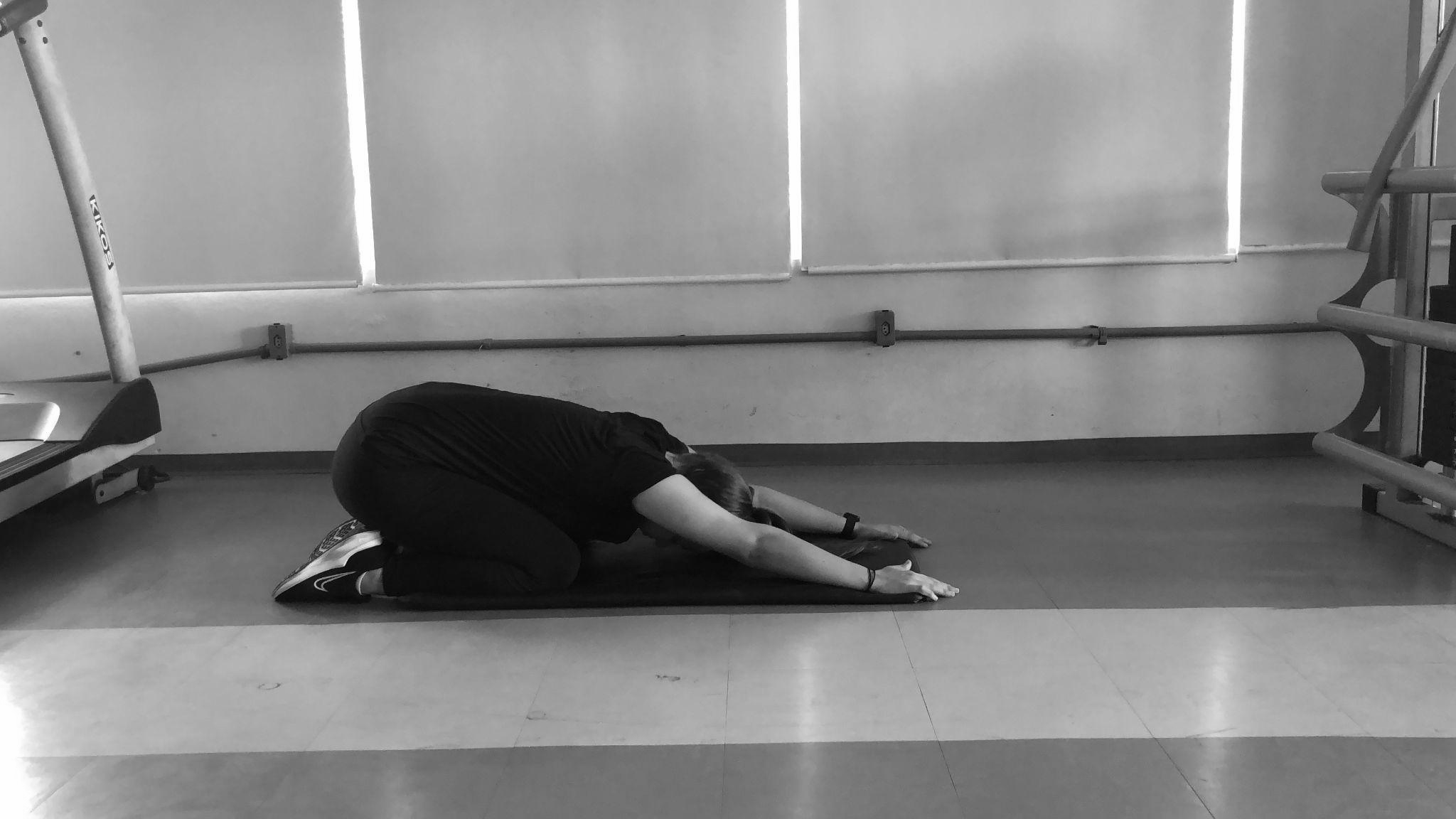** | | |
| **Knee flexors** | **Initial position:**  **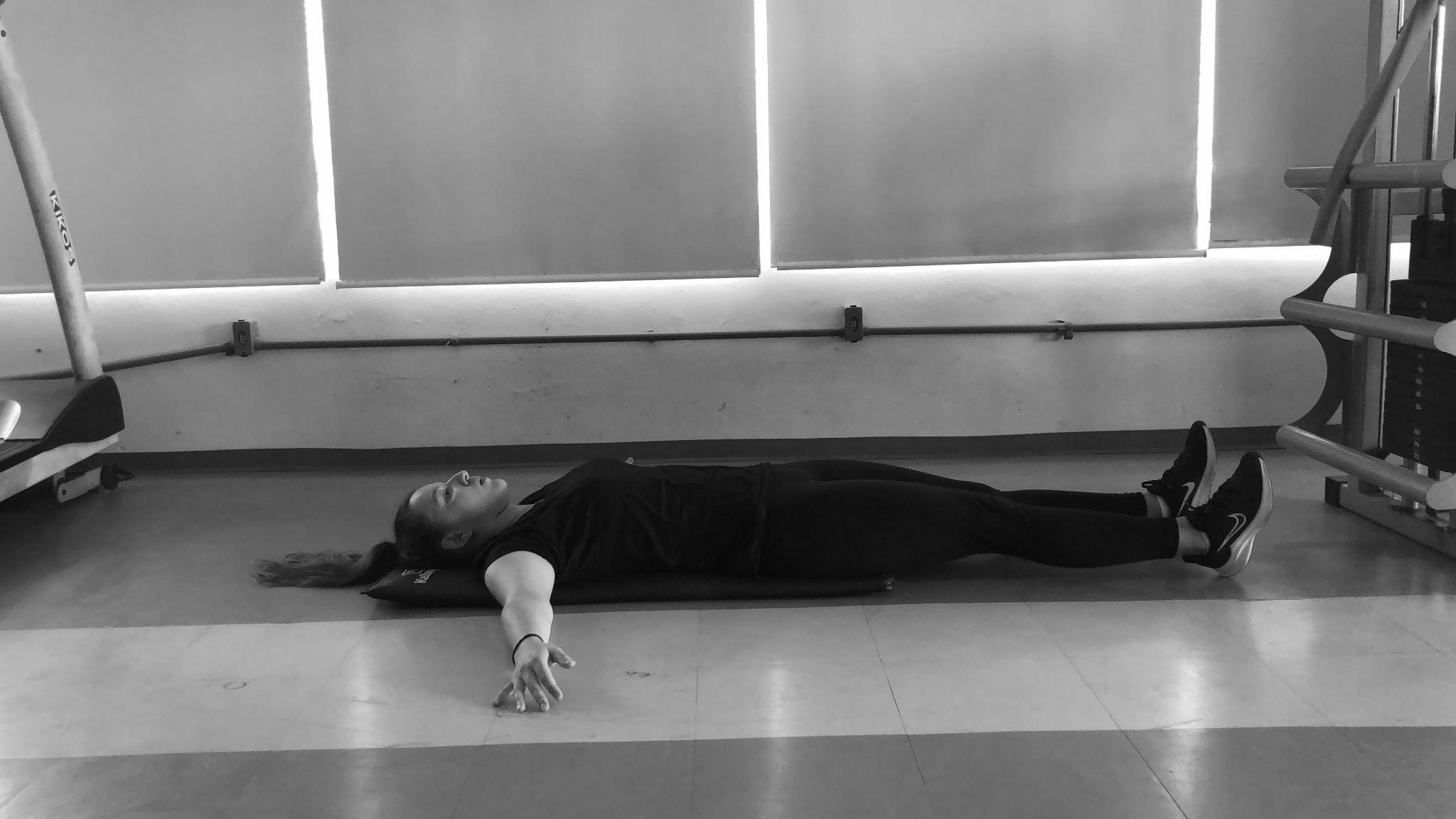** 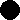 | **Final position:**  **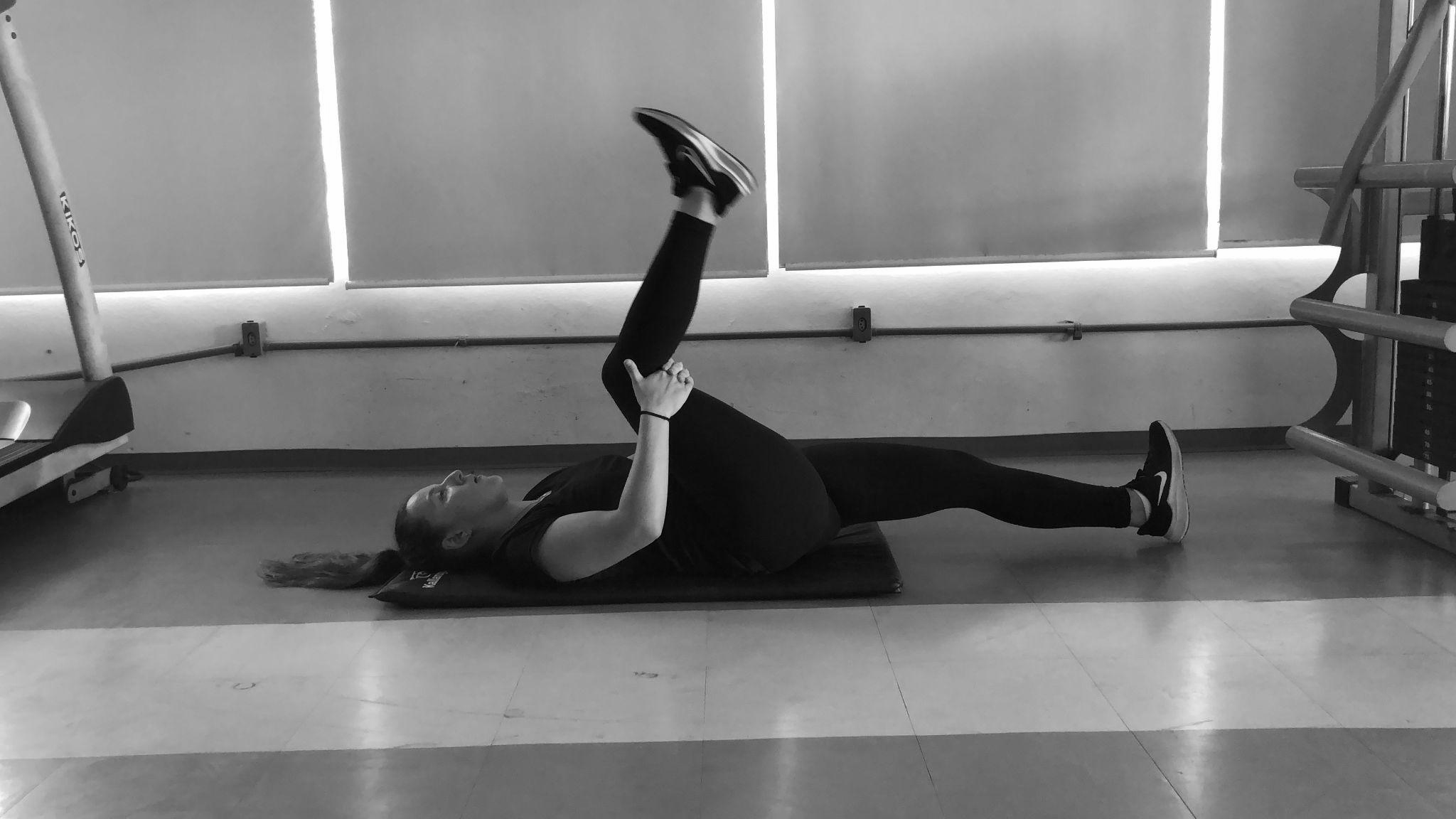** 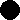 | | |
| **Glutes** | **Initial position:**  **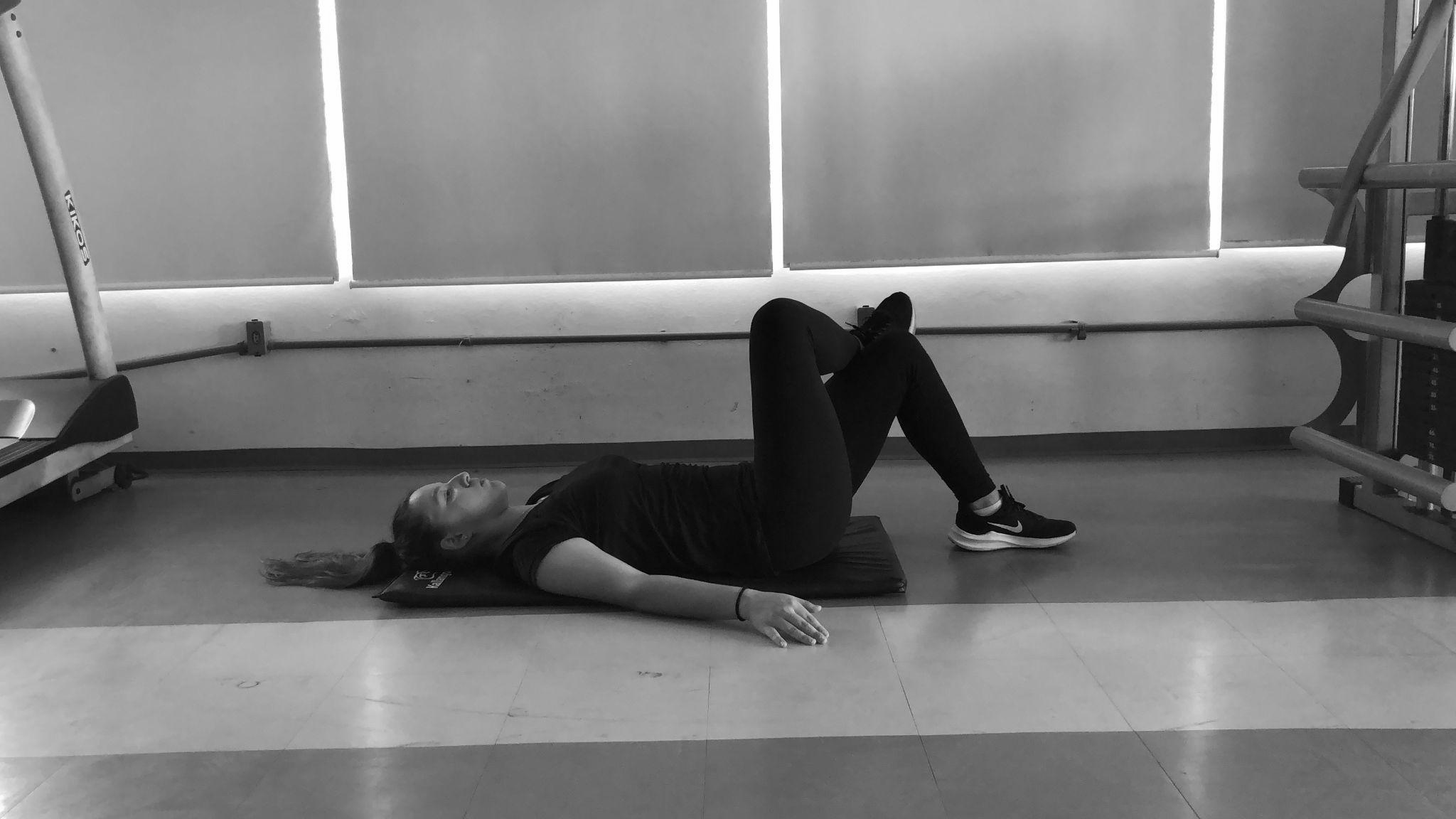** 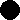 | **Final position:**  **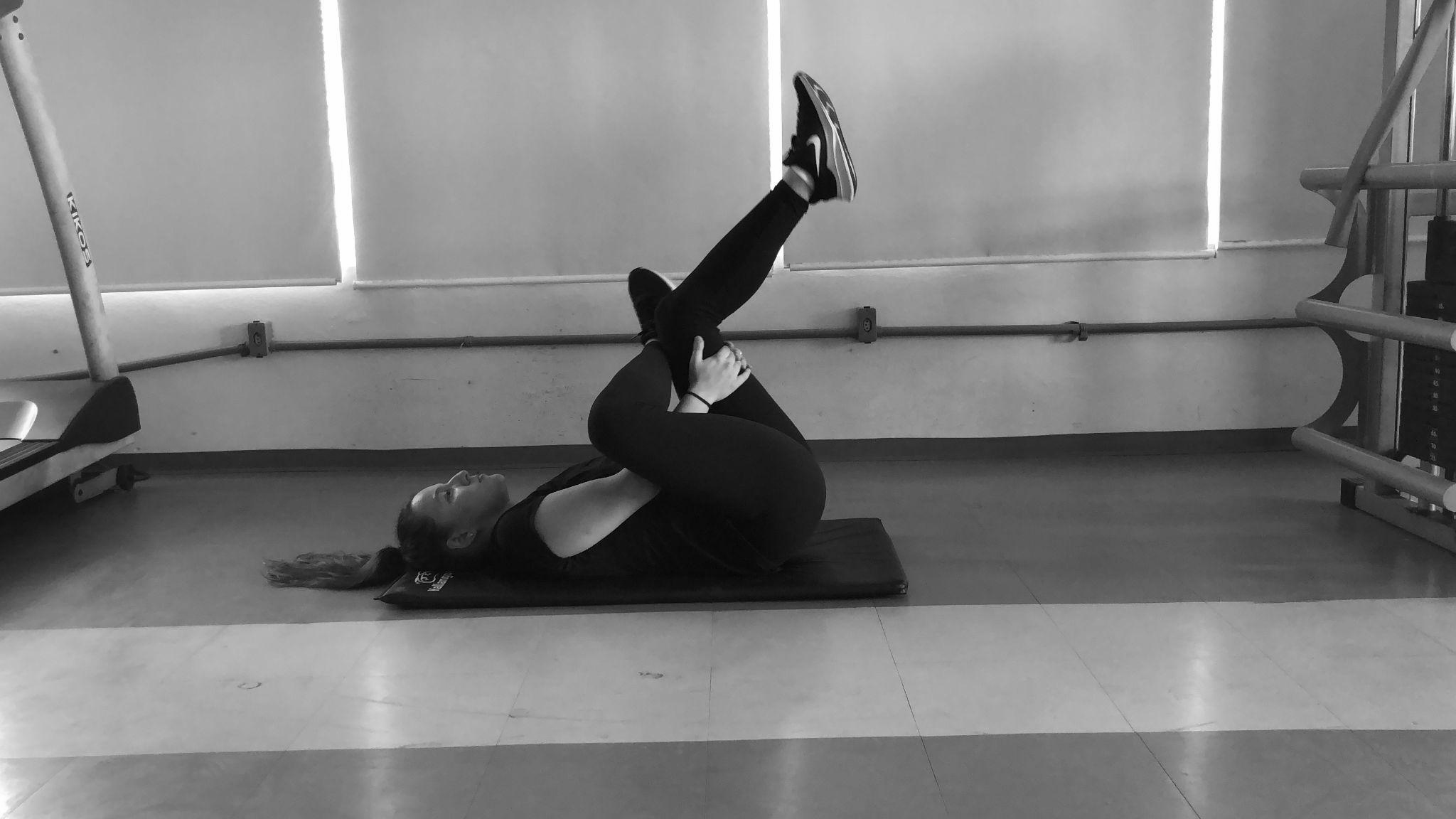** 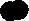 | | |
