# Supplementary material for: Effectiveness of m-health-based core strengthening exercise and health education for public safety workers with chronic non-specific low back pain: study protocol for a superiority randomized controlled trial (SAFEBACK)
Source: Trials. 2023 Dec 1;24:780. doi: 10.1186/s13063-023-07833-9 (PMC10693081; doi:10.1186/s13063-023-07833-9)
Supplement: Supplementary file 5 — Additional file 5. The back book. [file 13063_2023_7833_MOESM5_ESM.pdf]

THE BACK BOOK

The best and most up-to-date advice on how to

Deal with your backache yourself

Recover quickly and keep moving

Stay active and avoid disability

Help yourself to lead a normal life

Based on the latest medical evidence

© The Stationery Office Ltd
Applications for reproduction should be made to The Stationery Office Ltd,
St Crispins, Duke Street, Norwich NR3 1PD

The Publisher is not responsible for any statement made in this publication. Advice is offered by the authors for information only, and is not intended for use without independent substantiating investigation on the part of the potential users.

The help and advice from colleagues too numerous to mention is gratefully acknowledged.

To order copies of this or any other titles from The Stationery Office please call 0870 600 5522.

The Stationery Office
PO Box 29, Norwich
NR3 1GN

Single copies ISBN 0 11 322312 9 Price £1.25
Packs of 10 copies ISBN 0 11 702078 8 Price £12.50
Discounts apply for larger quantities

We can now send you updates on your specific areas of interest by email.
To register, visit www.thestationeryoffice.com

The New Approach to Back Pain

Back pain has always been very common and we have learned a great deal about it. There has been a revolution in thinking about back care, and we now approach it in a different way.

Most people can and do deal with back pain themselves most of the time. This booklet gives you the best and most up-to-date advice on how to deal with it, avoid disability and recover quickly. It is based on the latest research.

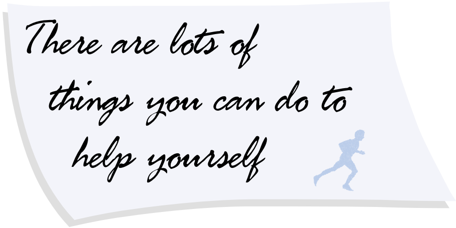

BACK FACTS

- Back pain or ache is usually not due to any serious disease.

- Most back pain settles quickly, at least enough to get on with your normal life.

> *Back pain need not cripple you unless you let it!*

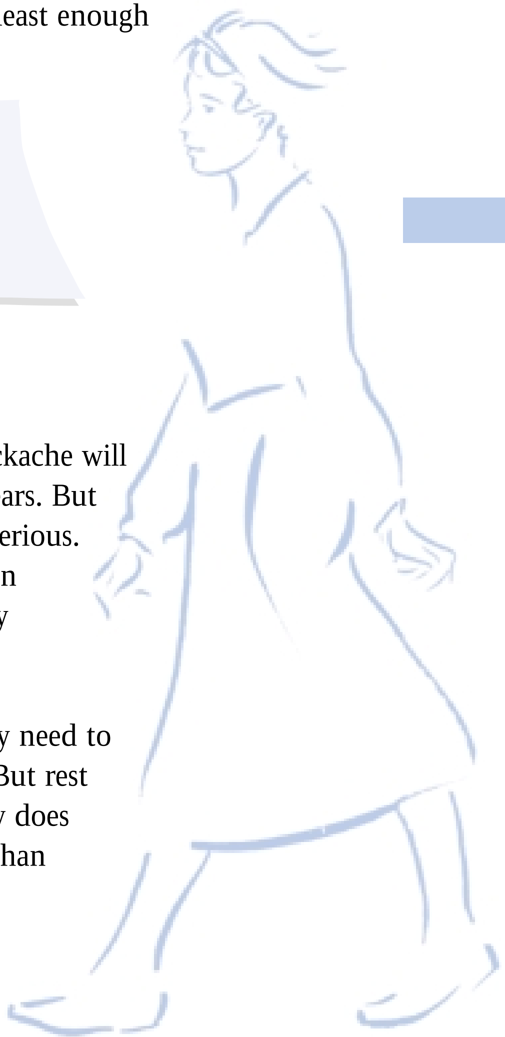

- About half the people who get backache will have it again within a couple of years. But that still does not mean that it is serious. Between attacks most people return to normal activities with few if any symptoms.

- It can be very painful and you may need to reduce some activities for a time. But rest for more than a day or two usually does not help and may do more harm than good. So keep moving.

- Your back is designed for movement. The sooner you get back to normal activity the sooner your back will feel better.

- The people who cope best are those who stay active and get on with their life despite the pain.

CAUSES OF BACK PAIN

Your spine is one of the strongest parts of your body. It is made of solid bony blocks joined by discs to give it strength and flexibility. It is reinforced by strong ligaments. It is surrounded by large and powerful muscles which protect it. It is surprisingly difficult to damage your spine.

People often have it wrong about back pain.

In fact:

> *It is surprisingly difficult to damage your spine*

- Most people with back pain or backache do not have any damage in their spine.

- Very few people with backache have a slipped disc or a trapped nerve. Even then a slipped disc usually gets better by itself.

- Most x-ray findings in your back are normal changes with age. That is not arthritis – it is normal, just like grey hair.

Back pain is usually not due to anything serious

In most people we cannot pinpoint the exact source of the trouble. It can be frustrating not to know exactly what is wrong. But in another way it is good news – you do not have any serious disease or any serious damage in your back.

Most back pain comes from the muscles, ligaments and joints in your back. They are simply not moving and working as they should. You can think of your back as ‘out of condition’. So what you need to do is get your back working properly again.

Stress can increase the amount of pain you feel. Tension can cause muscle spasm and the muscles themselves can become painful.

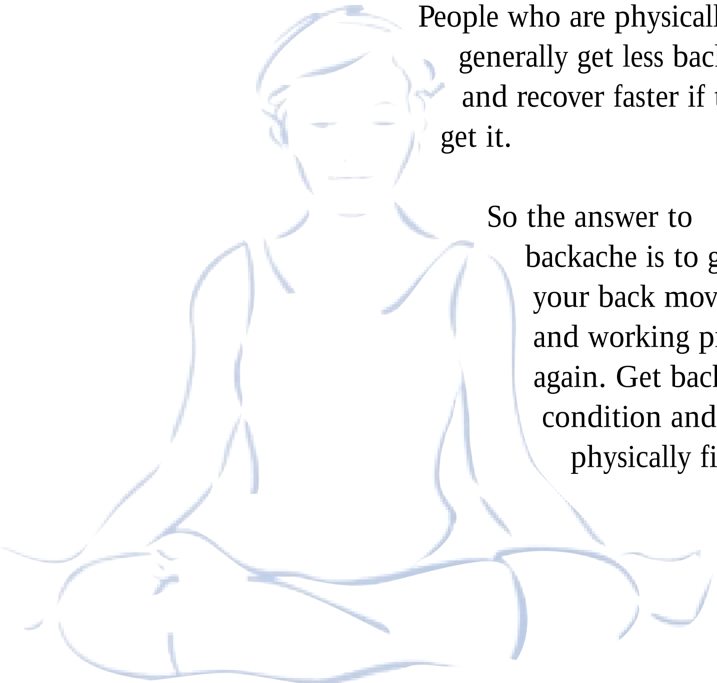

People who are physically fit generally get less back pain, and recover faster if they do get it.

So the answer to backache is to get your back moving and working properly again. Get back into condition and physically fit.

It's your back - get going!

REST OR ACTIVE EXERCISE ?

The old fashioned treatment for back pain was prolonged rest. But bed rest for more than a day or two is not good, because:

Bed rest is bad for backs

- Your bones get weaker.
- Your muscles get weaker.
- You get stiff.
- You lose physical fitness.
- You get depressed.
- The pain feels worse.
- It is harder and harder to get going again.

No wonder it didn't work! We no longer use bed rest to treat any other common condition. It is time to stop bed rest for backache. The message is clear: bed rest is bad for backs.

Of course, you might need to do a bit less when the pain is bad. You might be forced to have a day or two in bed at the start. But the most important thing is to get moving again as soon as you can.

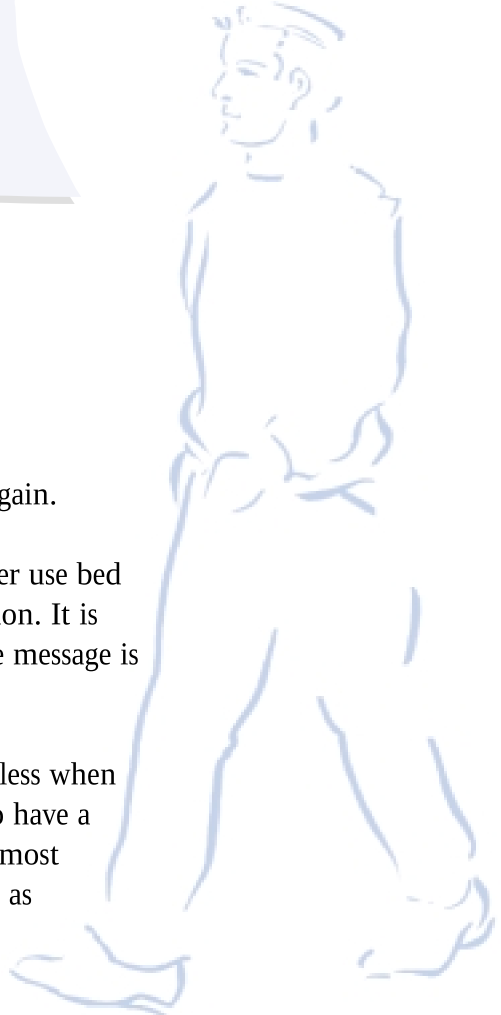

EXERCISE IS GOOD FOR YOU

Use it or lose it

Your body must stay active to stay healthy. It thrives on use.

Regular exercise:

- Gives you stronger bones.
- Develops fit active muscles.
- Keeps you supple.
- Makes you fit.
- Makes you feel good.
- Releases natural chemicals which reduce pain.

Even when your back is sore, you can make a start without putting too much stress on your back:

- Walking.
- Exercise bike.
- Swimming.

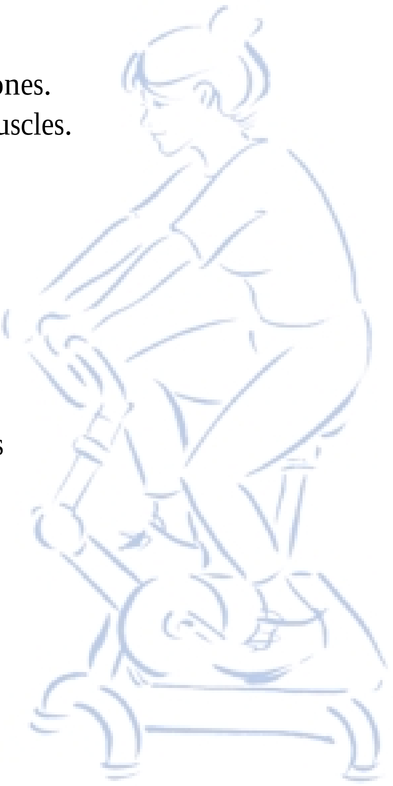

Walking, using an exercise bike, or swimming all use your muscles and get your joints moving. They make your heart and lungs work and are a start to physical fitness.

When you start to exercise you may need to build up gradually over a few days or weeks. You should then exercise regularly and keep it up – fitness takes time.

Different exercises suit different people. Find out what suits your back best. Rearrange your life to get some exercise every day. Try walking instead of going by car or bus. Some of the easiest activities to get back to are walking, swimming, cycling and smooth rhythmic exercises. The important thing is general exercise and physical fitness.

Athletes know that when they start training, their muscles can ache. That does not mean that they are doing any damage. The same applies to you and your back.

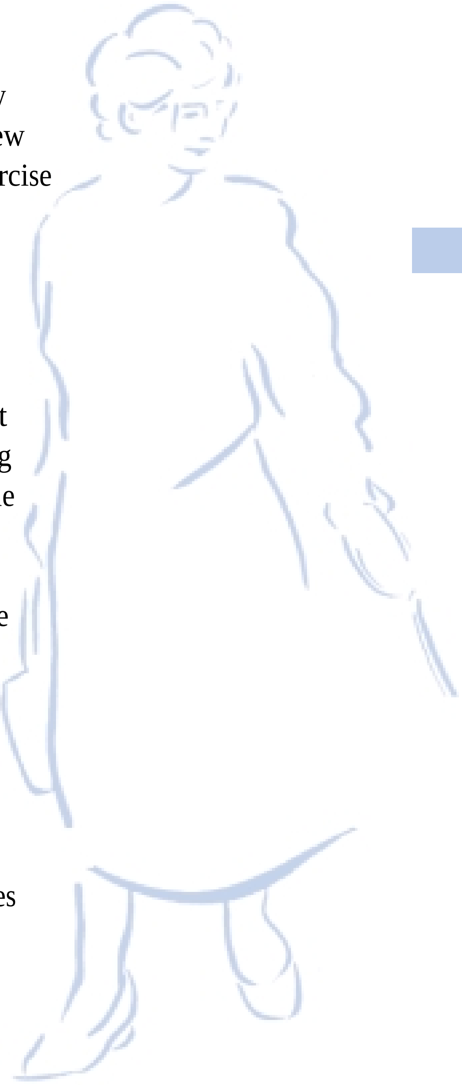

No-one pretends exercising is easy. Pain killers and other treatments can help to control the pain to let you get started. It often does hurt at first, but one thing is sure: the longer you put off exercise the harder and more painful it will be. There is no other way. You have a straight choice: rest, or work through your pain and recovery.

STAYING ACTIVE

Dealing with an acute attack

What you do depends on how bad your back feels.

Remember, your back isn't badly damaged.

You can usually:

- Use something to control the pain.
- Modify your activities.
- Stay active and at work.

You may have good days & bad days - that's normal

If your pain is more severe you may have to rest for a few days. You might need stronger pain killers from your doctor, and you might even have to lie down for a day or two. But only for a day or two; don't think of rest as treatment. Too much rest is bad for your back. The faster you get going the sooner your back will feel better.

You should build up your activities and your exercise tolerance over several days or a few weeks. But the faster you get back to normal activities and back to work the better, even if you still have some pain and some restrictions. If you have a heavy job, you may need some help from your work mates. Simple changes can make your job easier. Talk to your foreman or boss if you need to.

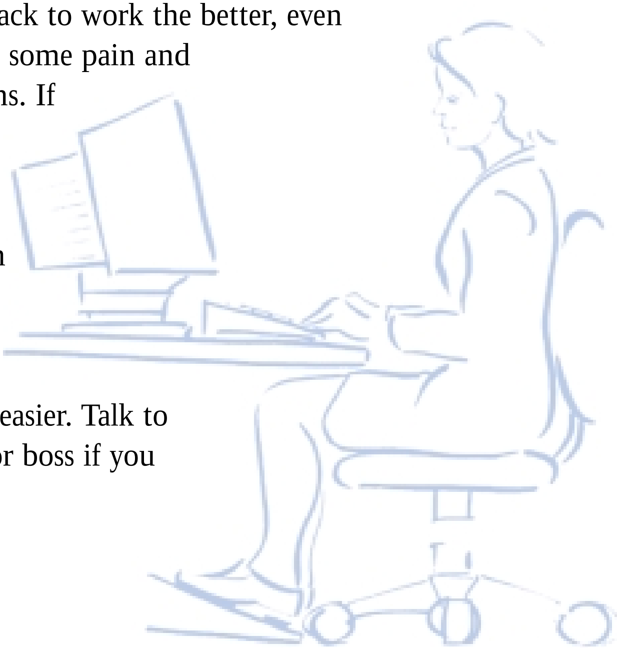

Control of pain

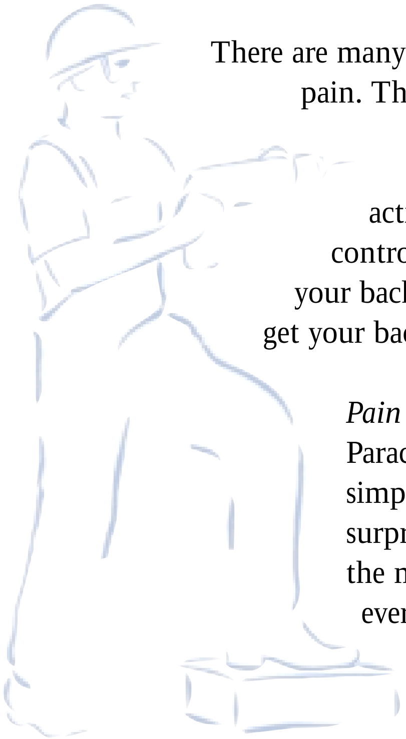

There are many treatments which can help back pain. They may not remove the pain completely, but they should control it enough for you to get active. These treatments help to control the pain, but they do not cure your back. It is up to you to get going and get your back working again.

Pain killers

Paracetamol or soluble Aspirin are the simplest and safest pain killers. It may surprise you, but they are still often the most effective. Take two tablets every 4-6 hours. Or you can use anti-inflammatory tablets like Ibuprofen.

You should usually take the pain killers for a day or two but you may need to take them for a few weeks. Take them regularly and do not wait till your pain is out of control. Do not take Aspirin or Ibuprofen if you have indigestion or an ulcer problem.

Heat or cold
In the first 48 hours you can try a cold pack on your back for 5-10 minutes at a time – a bag of frozen peas wrapped in a towel. Other people prefer heat – a hot water bottle, a bath or a shower.

Spinal manipulation
Most doctors now agree that manipulation can help. It is best within the first 6 weeks. Manipulation is carried out by osteopaths, chiropractors, some physiotherapists and a few doctors with special training. It is safe if it is done by a qualified professional.

Other treatments
Many other treatments are used and some people feel that they help. It is up to you to find out what helps you.

Stress and muscle tension
If stress is a problem you need to recognise it at an early stage and try to do something about it. It is not always possible to remove the cause of stress, but it is quite easy to learn to reduce its effects by breathing control, muscle relaxation and mental calming techniques.

HOW TO STAY ACTIVE

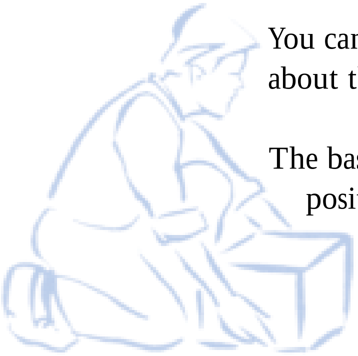

You can do most daily activities if you think about them first.

The basic idea is not to stay in one position or do any one thing for more than 20-30 minutes without a break. Then try to move a little further and faster every day.

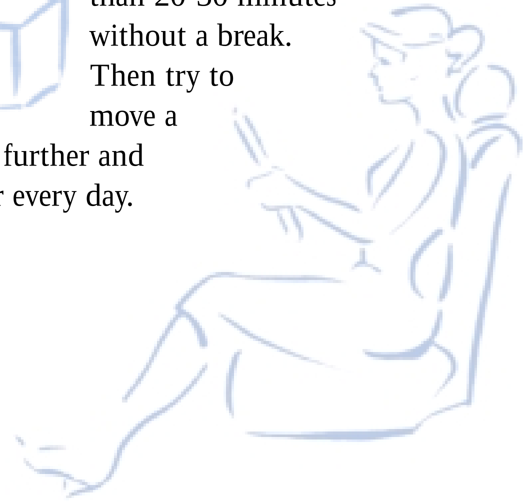

	☺ *Some ways to help your pain*	☹ *Some things may make your pain worse*
Lifting	Know your own strength: lift what you can handle.<br>Always lift and carry close to your body.<br>Bend your knees and make your legs do the work.<br>Don’t twist your back – turn with your feet.	Lifting without thinking
Sitting	Use an upright chair.<br>Try a folded towel in the small of your back.<br>Get up and stretch every 20-30 minutes.	A low, soft chair.<br>Lack of back support.<br>Sitting for a long time.
Standing	Try putting one foot on a low box or stool.<br>Have your working surface at a comfortable height.	Long periods in one position.
Driving	Adjust your seat from time to time.<br>Try a folded towel in the small of your back.	Long drives without a break.
Activity	20-30 minutes walking, cycling or swimming every day.<br>Gradually increase physical activity.	Sitting around all day.<br>Not exercising: being unfit.
Sleeping	Some people prefer a firm mattress –<br>or try boards beneath your mattress.	Staying in bed too long.
Relax	Learn how to reduce stress.<br>Use relaxation techniques.	Worry: being tense.

What do you find helps you?

1 ______________________________

2 ______________________________

3 ______________________________

4 ______________________________

5 ______________________________

What do you find makes your pain worse?

1 ______________________________

2 ______________________________

3 ______________________________

4 ______________________________

5 ______________________________

You can deal with most back pain yourself, but there are times when you should see your doctor.

What doctors can and can’t do

Doctors can diagnose and treat the few serious spinal diseases. But they have no quick fix for simple back pain. You must be realistic about what you can expect from your doctor and therapist.

- They can reassure you that you do not have any serious disease.
- They can try various treatments to help control your pain.
- They can advise you on how you can best deal with the pain and get on with your life.

It is natural to worry that back pain might be due to something serious. Usually it isn’t. But you may still feel the need to check. That is one of the most important things that your doctor can do for you.

Doctors can support and help you but it's your back and it is up to you to get it going!

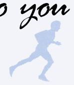

Warning signs

If you have severe pain which gets worse over several weeks instead of better, or if you are unwell with back pain, you should see your doctor.

Here are a few symptoms which are all very rare but if you do have back pain and suddenly develop any of these symptoms you should see a doctor straight away.

- Difficulty passing or controlling urine.
- Numbness around your back passage or genitals.
- Numbness, pins and needles or weakness in both legs.
- Unsteadiness on your feet.

Don't let that list worry you too much.

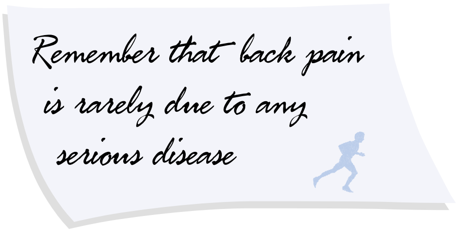

Backache is not a serious disease and it should not cripple you unless you let it. We have tried to show you the best way to deal with it. The important thing now is for you to get on with your life. How your backache affects you depends on how you react to the pain and what you do about it yourself.

There is no instant answer. You will have your ups and downs for a while – that is normal. But this is what you can do for yourself.

There are two types of sufferer

One who avoids activity,
and one who copes.

- ☹ The *avoider* gets frightened by the pain and worries about the future.
- The *avoider* is afraid that hurting always means further damage – it doesn't.
- The *avoider* rests a lot, and waits for the pain to get better.

- ☺ The *coper* knows that the pain will get better and does not fear the future.
- The *coper* carries on as normally as possible.
- The *coper* deals with the pain by being positive, staying active or staying at work.

Who suffers most?

☹ *Avoiders* suffer the most. They have pain for longer, they have more time off work and they can become disabled.

☺ *Copers* suffer less at the time and they are healthier in the long run.

So how do I become a *Coper* and prevent unnecessary suffering?

Follow these guidelines – you really can help yourself.

☺ Live life as normally as possible. This is much better than staying in bed.

- Keep up daily activities – they will not cause damage. Just avoid really heavy things.

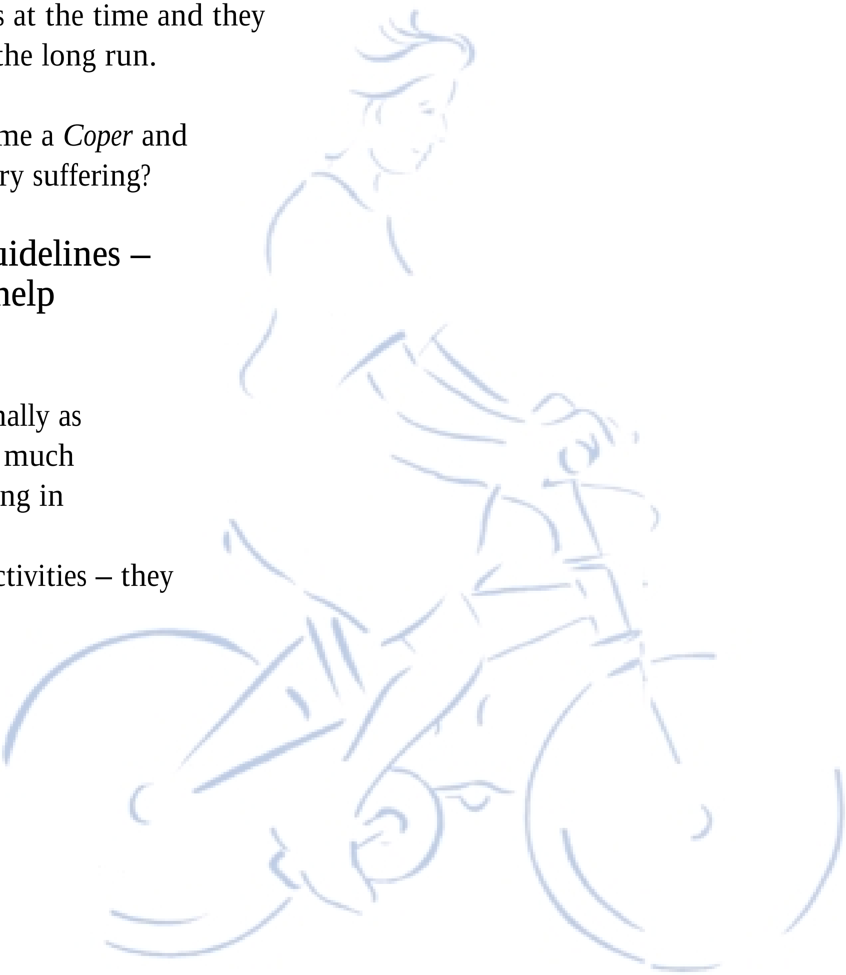

- Try to stay fit – walking, cycling or swimming will exercise your back and should make you feel better. And continue even after your back feels better.
- Start gradually and do a little more each day so you can see the progress you are making.
- Either stay at work or go back to work as soon as possible. If necessary, ask if you can get lighter duties for a week or two.
- Be patient. It is normal to get aches or twinges for a time.

☺ Don't just rely on pain killers. Stay positive and take control of the pain yourself.

- Don't stay at home or give up doing things you enjoy.
- Don't worry. It does not mean you are going to become an invalid.
- Don't listen to other people's horror stories – they're usually nonsense.
- Don't get gloomy on the down days.

Be positive and stay active, you will get better quicker and have less trouble later.

Remember:

- Back pain is common but it is rarely due to any serious disease.
- Even when it is very painful that usually does not mean there is any serious damage to your back. Hurt does not mean harm.
- Mostly it gets better with little or no medical treatment.
- Bed rest for more than a day or two is usually bad for your back.
- Staying active will help you get better faster and prevent more back trouble.

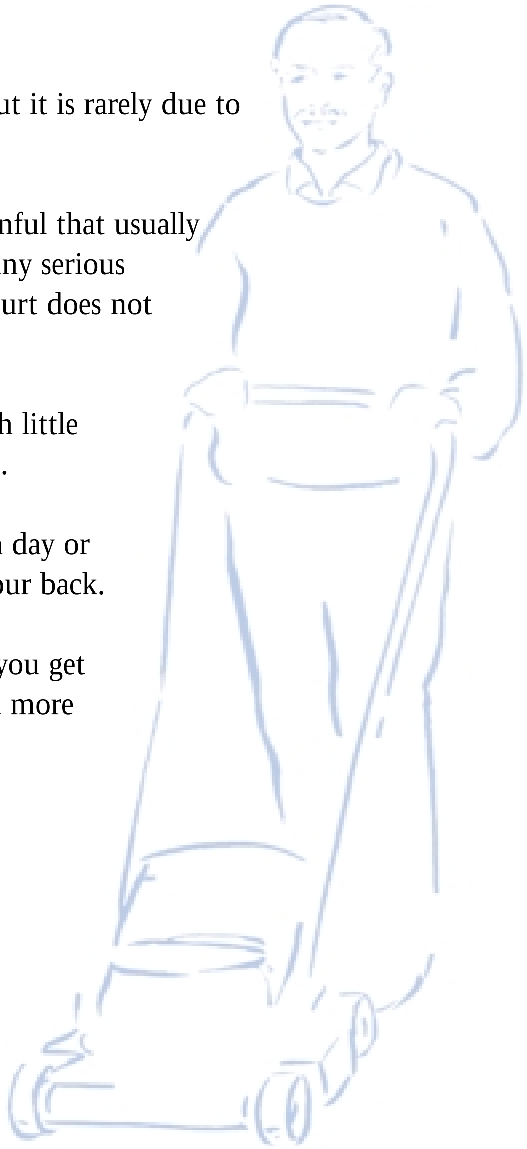

- The sooner you get going, the sooner you will get better.
- Regular exercise and staying fit helps your general health and your back.
- You have to run your own life and do the things you want to do. Don't let your back take over.

That's the message from the latest research - you really can help yourself

Printed in the United Kingdom by The Stationery Office
TJ2178 C1110 08/00

There is a great demand for effective information for back pain patients, and *The Back Book* has been produced as a direct response to that demand. Designed as a guide for patients, the advice it contains is based on the latest medical evidence.

It is intended that *The Back Book* will be given to patients by their doctor or therapist to help them in the early management of their back pain. It is linked with the Royal College of General Practitioners *Clinical Guidelines for the Management of Acute Low Back Pain.*

To be sure that it contains all the latest thinking on back pain, this book has been written by a multi disciplinary team of researchers:
General Practice, *Prof Martin Roland*, Manchester.
Orthopaedics, *Prof Gordon Waddell*, Glasgow.
Physiotherapy, *Dr Jennifer Klaber Moffett*, York.
Osteopathy, *Dr Kim Burton*, Huddersfield.
Psychology, *Dr Chris Main*, Salford.
Rehabilitation *Dr Ted Cantrell*, Southampton.

The Stationery Office

ISBN 0-11-322312-9

9 780113 223121

£1.25
