# Supplementary material for: Effectiveness of m-health-based core strengthening exercise and health education for public safety workers with chronic non-specific low back pain: study protocol for a superiority randomized controlled trial (SAFEBACK)
Source: Trials. 2023 Dec 1;24:780. doi: 10.1186/s13063-023-07833-9 (PMC10693081; doi:10.1186/s13063-023-07833-9)
Supplement: Supplementary file 6 — Additional file 6. Amendment’s chronology. [file 13063_2023_7833_MOESM6_ESM.docx]

**Additional file 6 -** Amendment’s chronology

Protocol amendment number: 01

Authors: Eduardo F. Marins

Issue date: 10 February 2023

Primary reasons for amendment: We will not measure the followings secondary outcomes: occupational performance and muscular activation of internal oblique muscle.
